# Visualization of porosity and pore size gradients in electrospun scaffolds using laser metrology

**DOI:** 10.1371/journal.pone.0282903

**Published:** 2023-03-09

**Authors:** Yi-xiao Liu, Francisco J. Chaparro, Ziting Tian, Yizhen Jia, John Gosser, Jeremy Gaumer, Liam Ross, Hooman Tafreshi, John J. Lannutti

**Affiliations:** 1 Department of Materials Science and Engineering, The Ohio State University, Columbus, OH, United States of America; 2 Nanoscience Instruments, Phoenix, AZ, United States of America; 3 Tosoh SMD, Inc., Grove City, OH, United States of America; 4 Columbus Academy, Gahanna, OH, United States of America; 5 Department of Mechanical and Aerospace Engineering, North Carolina State University, Raleigh, NC, United States of America; 6 Department of Biomedical Engineering, The Ohio State University, Columbus, OH, United States of America; 7 Center for Chronic Brain Injury Program, The Ohio State University, Columbus, OH, United States of America; State University of New York at Binghamton: Binghamton University, UNITED STATES

## Abstract

We applied a recently developed method, laser metrology, to characterize the influence of collector rotation on porosity gradients of electrospun polycaprolactone (PCL) widely investigated for use in tissue engineering. The prior- and post-sintering dimensions of PCL scaffolds were compared to derive quantitative, spatially-resolved porosity ‘maps’ from net shrinkage. Deposited on a rotating mandrel (200 RPM), the central region of deposition reaches the highest porosity, ~92%, surrounded by approximately symmetrical decreases to ~89% at the edges. At 1100 RPM, a uniform porosity of ~88–89% is observed. At 2000 RPM, the lowest porosity, ~87%, is found in the middle of the deposition, rebounding to ~89% at the edges. Using a statistical model of random fiber network, we demonstrated that these relatively small changes in porosity values produce disproportionately large variations in pore size. The model predicts an exponential dependence of pore size on porosity when the scaffold is highly porous (e.g., >80%) and, accordingly, the observed porosity variation is associated with dramatic changes in pore size and ability to accommodate cell infiltration. Within the thickest regions most likely to ‘bottleneck’ cell infiltration, pore size decreases from ~37 to 23 μm (38%) when rotational speeds increased from 200 to 2000 RPM. This trend is corroborated by electron microscopy. While faster rotational speeds ultimately overcome axial alignment induced by cylindrical electric fields associated with the collector geometry, it does so at the cost of eliminating larger pores favoring cell infiltration. This puts the bio-mechanical advantages associated with collector rotation-induced alignment at odds with biological goals. A more significant decrease in pore size from ~54 to ~19 μm (65%), well below the minimum associated with cellular infiltration, is observed from enhanced collector biases. Finally, similar predictions show that sacrificial fiber approaches are inefficient in achieving cell-permissive pore sizes.

## Introduction

Scaffold fabrication via electrospinning offers diverse choices of natural and synthetic polymers, mild processing conditions, small energy and equipment footprints, and most importantly, the ability to directly replicate the microstructure of natural extracellular matrices. However, electrospinning often results in values of porosity preventing easy cellular infiltration [1, 2]. Common remedies include the use of salt-based porogens [1–4] or sacrificial fibers [1, 2, 5, 6] that should create the space needed for cell ingress and tissue development. More recently, complex gradients are being engineered into state-of-the-art scaffolds [7–12] to more closely mimic biological structures or to enable high-throughput screening. Despite these advances, characterization methods that provide comprehensive, spatially-resolved quantification of porosity are still lacking.

Determining the porosity of electrospun scaffolds is not as straightforward as measuring its fiber diameter [13]. Conventional electron microscopy lacks resolution over depth to correctly segment individual pores from the continuous 3D void [14]. Various tomography technologies [15–17] typically have very limited field-of-view when characterizing nanofibers and often require expensive instruments or facilities. Methods based on fluid displacement such as porosimetry [18] risk sample distortion and cannot resolve spatial gradients.

In light of this, we recently created a new technique allowing quantitative analysis–for the first time–of porosity within electrospun scaffolds versus both position on collector surface and associated fabrication conditions [19–21]. It was observed that porosity was markedly sensitive to even small changes in electrospinning conditions. Even within the same deposition, porosity across the surface could range from 0 to ~88% [20]. These values were found to be sensitive to many factors often controlled for during production (e.g., electrical bias) and others much less actively controlled (humidity, (multi-)needle placement, localized field strength).

In this paper, we use this new method to examine the effects of rotational speed, a commonly deployed process variable in the electrospinning of tubular constructs. At sufficiently high speeds, this gives rise to aligned fibers with mechanical properties sufficient for direct replacement of blood vessels [22–24]. Alignment is also believed to guide cell migration and differentiation [25, 26]. Exploring the interaction between these rotations and porosity should create new insights into electrospun scaffold designs. Three rotational speeds, 200, 1100, and 2000 RPM (0.099, 0.547, and 0.995 m/s linear speeds, respectively) were employed in this study. Fabricated scaffolds were densified via heat treatments, and their dimensional changes were characterized by laser metrology to calculate porosity. Shifts in both distribution and average values of porosity are observed as a function of these speeds. In this context, we re-examined the Sampson model [27] of random fiber network(s) and adapt it to show that these porosity variations can quickly diminish pore size and potential for cell ingression. We also reviewed the effect of electrical bias by applying the same model to results of our previous study [19]. Interestingly, periodic occurrence of cell-impermeable fiber bundles is also observed at 200 RPM.

## Materials and methods

### Scaffold preparation and electron microscopy

Scaffolds were prepared following previously-established procedure [19] detailed in the S1 File. Briefly, 5 wt% polycaprolactone (PCL) in hexafluoroisopropanol (HFP) solution was electrospun from a +10 kV charged needle onto ~0.95 cm diameter, -1 kV biased cylindrical rods at 6 mL/h for 22 minutes. Collector rotation at 200, 1100, and 2000 RPM was employed to investigate its influence on porosity and microstructure. Electrospinning was performed inside a Fluidnatek® LE-100 unit (Bioinicia-Fluidnatek) where environmental conditions were tightly controlled.

Fiber diameter and orientation was measured from scanning electron microscopy (SEM) images with Fibermetric (v2.3.4.0) and OrientationJ [28] (v2.0.5), respectively. Pore size was automatically measured with Fiji/ImageJ [29] (2.0.0-rc-69/1.52p) after binarization using DiameterJ [14]. Details of these measurements can be found in the S1 File.

### Laser metrology

The principles and design of our metrology system have been reported previously [19–21, 30]. Briefly, mandrel collectors are scanned by a laser micrometer (TLAser122s, Laserlinc) before and after electrospinning to obtain accurate dimension profiles of the tubular deposits. These depositions are then completely densified via a heat treatment of 65°C, 3 hours. A third scan was performed to profile the densified dimensions. The porosity is calculated from vertical shrinkage:

$$p_{\Phi ,Z}=\left[1-\frac{h_{\Phi ,Z}(2R_{\Phi ,Z}+h_{\Phi ,Z})}{H_{\Phi ,Z}(2R_{\Phi ,Z}+H_{\Phi ,Z})}\right]\times 100\%$$ (1)

*p*_*Φ*,*Z*_ describes the porosity distribution versus both azimuthal (the circumferential direction, Φ = 0, 5, 10…, 355°) and axial (the longitudinal direction, Z = 0, 1, 2…, 200 mm) coordinates. The same applies to the collector radius (*R*_*Φ*,*Z*_), as-deposited (*H*_*Φ*,*Z*_) and densified (*h*_*Φ*,*Z*_) thickness profiles. Regions outside 20<Z<180 mm containing markers and engravings that facilitates identification and 3D reconstruction were excluded from data processing and illustration. Any porosity values where *H*_*Φ*,*Z*_, *h*_*Φ*,*Z*_, or |*H*_*Φ*,*Z*_ − *h*_*Φ*,*Z*_| is less than or equal to twice of the accuracy of the laser micrometer (±2.5 μm) were also discarded.

To quantify the surface roughness presented in as-spun depositions, *Ra* is first calculated along the circumferential/azimuthal direction:

$${Ra}_{Z}=\frac{1}{72}\sum_{\Phi }\left|H_{\Phi ,Z}-{\bar{H}}_{Z}\right|$$ (2)

where ${\bar{H}}_{Z}$ is the mean thickness across all azimuths at a particular *Z*. The mean of all *Ra*_*Z*_ is then reported as the average *Ra* of a deposition.

Pore size is further estimated from the relation proposed for two-dimensional random fiber networks [27]:

$${\bar{d}}_{\Phi ,Z}=\frac{2}{\ln\left({1/p}_{\Phi ,Z}\right)}d_{f}$$ (3)

where *d*_*f*_ is the fiber diameter and ${\bar{d}}_{\Phi ,Z}$ is the mean pore diameter.

### Statistical analysis

Electrospinning deposition was repeated 5 times (4 for laser profiling, 1 for SEM) for each rotational speed. One deposition from the 1100 RPM group displayed distinctly different characters (S2 Fig in S1 File) from the others and was excluded from this study. This may be due to the extra polishing the specific mandrel underwent to make it fit into experiment apparatus. The resulting variation in contact resistances may have led to anomaly in deposition characteristics. Validation of this hypothesis is being explored but out of scope of the current study.

All statistical analysis was performed using R (v4.0.3). Considering data heteroscedasticity (e.g., ~20× difference in standard deviation of peak thickness between 200 and 2000 RPMs, Table 1), we chose Welch’s ANOVA to determine statistical significance with Games-Howell test for multiple comparison. For fiber diameters and pore sizes, Kruskal-Wallis test with Dunn’s *post hoc* test (Benjamini-Hochberg corrected) was performed to accommodate data non-normality. p<0.01 was defined as statistically significant while p<0.05 was regarded as mildly significant.

**Table 1:** Values of thickness, Ra, spread, porosity average, and fiber diameter.

RPM	Deposit Peak Thickness (μm)*†	Ra (μm)*	Spread (mm)*§	Average Porosity (%)*	Fiber Diameter–Center (μm)**	Fiber Diameter–Edge (μm)**
200	838.5±101.7^a^	7.5±1.8^a^	104.4±4.4^a^	90.2±0.2^A^	1.63[1.21–2.03]^a^	1.39[1.07–1.73]^C^
1100	558.9±14.2^Ab^	1.2±0.3^b^	105.1±3.3^a^	88.9±0.2^Ba^	1.56[1.20–1.94]^b^	1.51[1.05–1.86]^B^
2000	463.4±4.7^Bc^	0.9±0.1^b^	106.1±0.6^a^	87.6±0.0^Bb^	1.59[1.20–1.98]	1.65[1.28–2.01]^A^
p-value	0.001	0.011	0.709	<0.001	0.013	<0.001

Values in the same column having different capital (A, B, C) or lower-case letters (a, b, c) in superscripts are statistically different at the 0.01 or 0.05 significance levels, respectively.

*Mean ± standard deviation of 4 (200 and 2000 RPM) or 3 (1100 RPM, see S1 File) individual spins. Statistical differences analyzed via Welch’s ANOVA followed by Games-Howell post hoc test.

** the median [interquartile range] of 2500+ measurements. Statistical difference analyzed via Kruskal-Wallis test followed by Dunn’s test with Benjamini-Hochberg correction for pairwise comparison.

† The peak thickness value of 200 RPM deposition is inflated by the presence of surface protrusions.

§ Defined as the span along the axial direction (Z) where more than 100 μm of deposits are observed.

## Results

**Fig 1:**
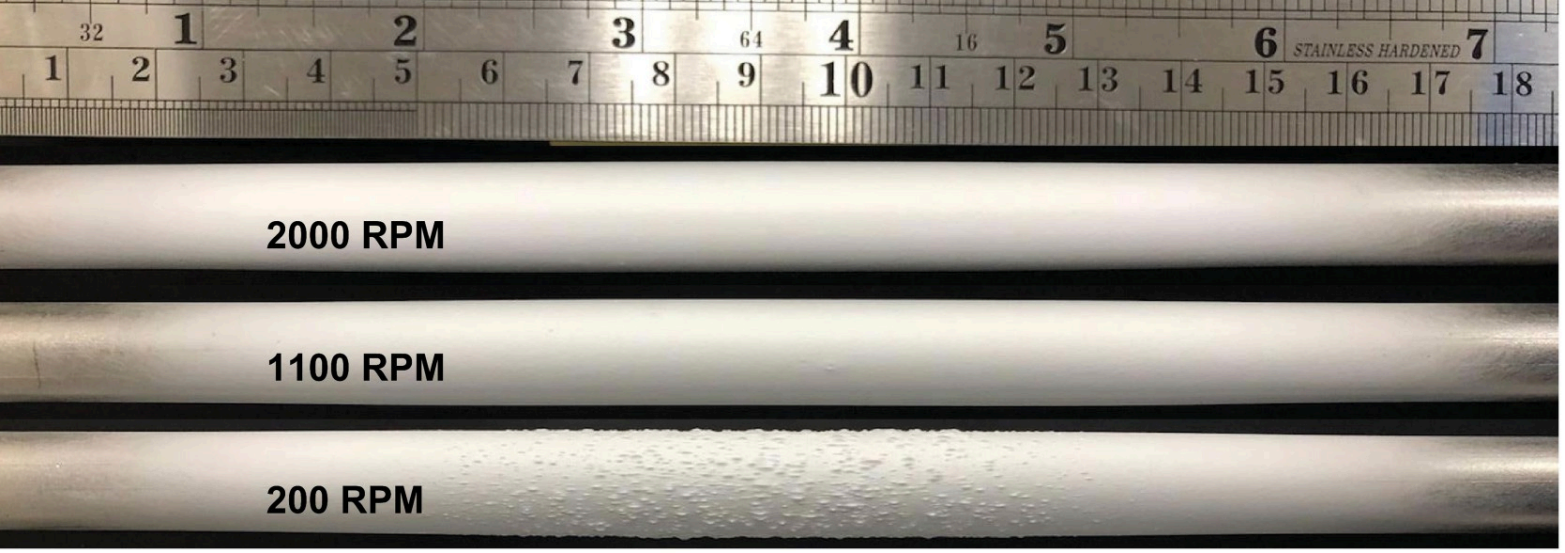
Optical image of example depositions produced by electrospinning onto the 200, 1100, and 2000 RPM rods. All depositions span approximately the same length of the mandrel.

**Fig 2:**
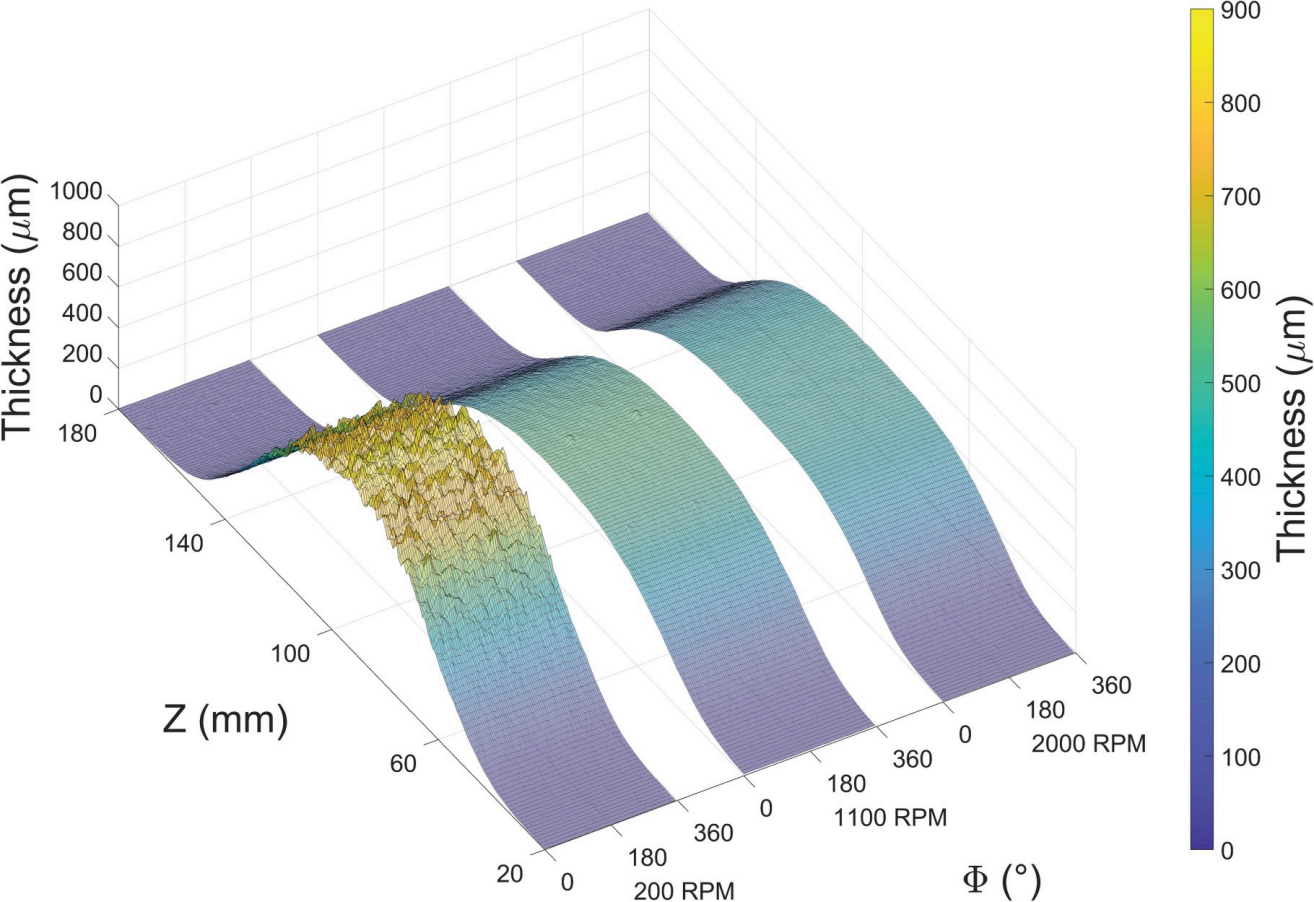
Thickness maps of example depositions onto 200, 1100, and 2000 RPM mandrels plotted against both the azimuthal (Φ, circumferential) and axial (Z, longitudinal) directions.

Following deposition, the 3 different RPMs produced distinct visual characters (Fig 1). Although they span essentially the same length along the mandrel, surface ‘bumps’ are visually prevalent in the center of 200 RPM deposition while 1100 and 2000 RPM depositions generally appear smooth. Both features are also evident in the 3D reconstruction (Fig 2) based on laser metrology, where as-deposited thickness is plotted against both azimuthal and axial directions. The 200 RPM thickness appears gaussian with significant roughness in the central region (~70<Z<120 mm). In contrast, the 1100 and 2000 RPM depositions share a smooth, flattened top in the central region. Outside these regions, all depositions appear quite similar. The numerical values of these characteristics are summarized in Table 1.

**Fig 3:**
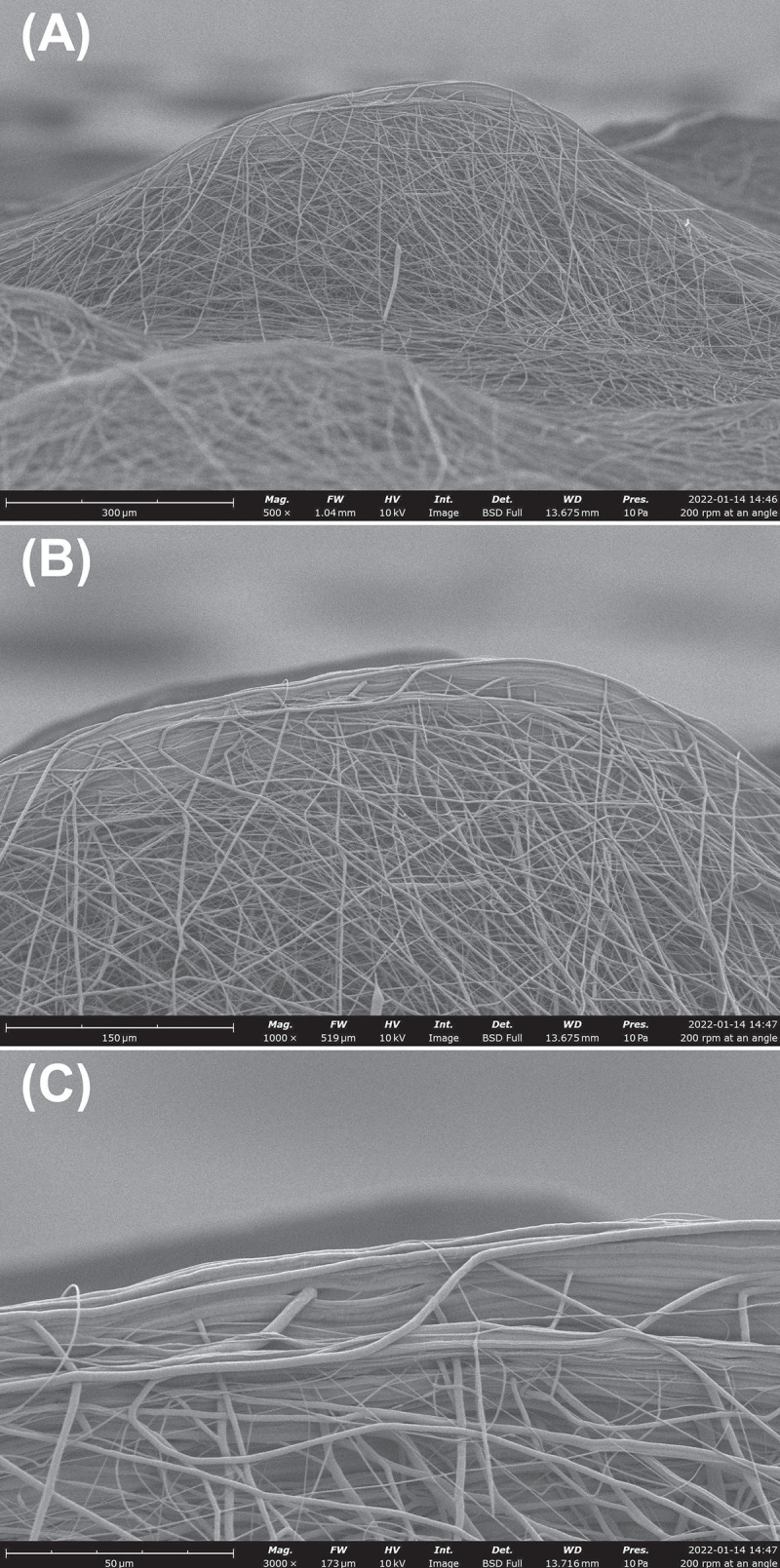
SEM images of surface protrusions found following 200 RPM depositions. (A) Low magnification showing the preponderance of these protrusions and their frequency. (B, C) Higher resolution images revealing the aligned fiber arches found at the ‘peak’ of these features. Images are taken at a ~90° tilt.

Fig 3 depicts the surprisingly pronounced vertical component of surface protrusions present in 200 RPM depositions: arches containing exquisitely aligned fibers are stacked on top of one another. We were unable to locate similar features at higher RPMs. The periodic occurrence (200 RPM) or absence (1100 and 2000 RPMs) of these features aligns with the roughness (or lack thereof) shown in Figs 1 and 2 and *Ra* values reported in Table 1.

**Fig 4:**
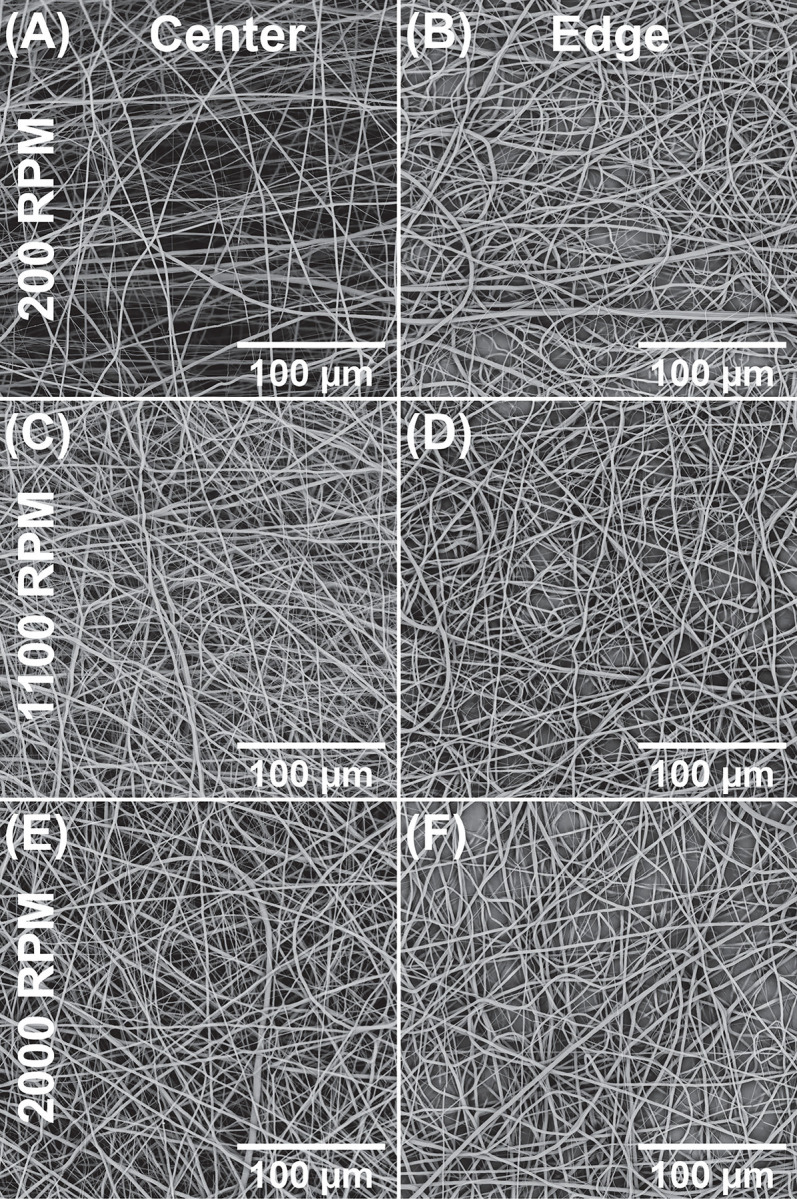
SEM images of the central and edge regions following 200, 1100, and 2000 RPM depositions.

Outside of these protrusions, all three RPMs display relatively normal arrangements of smooth, highly cylindrical fibers typical of the PCL-HFP system. Fig 4 illustrates representative SEM images following 200 (A-B), 1100 (C-D), and 2000 (E-F) RPM depositions taken at the center (A, C, E) or near the edge (B, D, F) of these depositions. In particular, the center of the 200 RPM deposition is visually more porous. Less obvious is that a significant portion of fibers are ‘curly’ near edges indicative of buckling [31].

**Fig 5:**
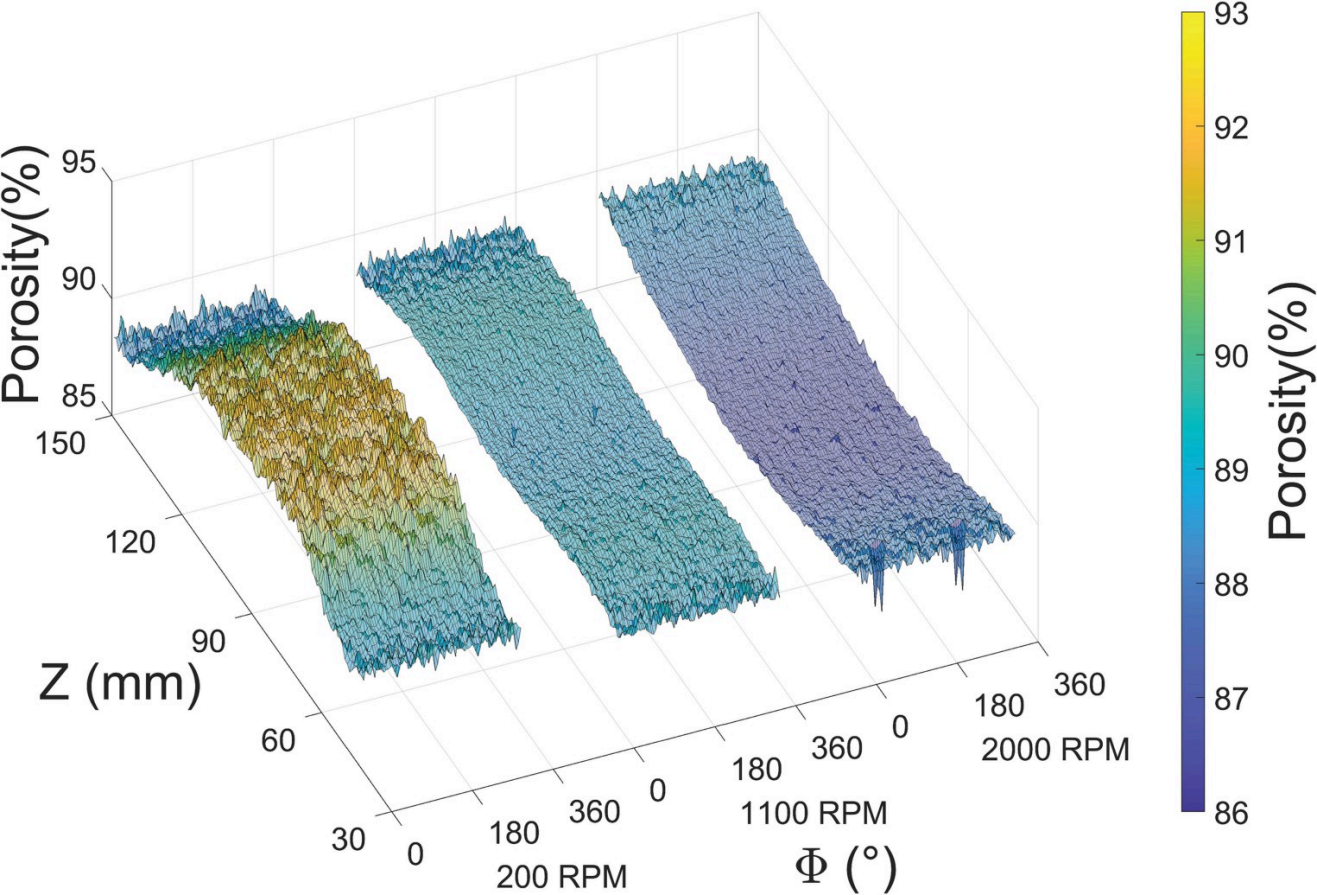
Porosity maps of example depositions on mandrels rotated at 200, 1100 and 2000 RPM plotted against both the azimuthal (Φ, circumferential) and axial (Z, longitudinal) directions.

To obtain quantitative information regarding the porosity variations observed under SEM, we sintered these depositions to eliminate internal pores and calculated porosity based on the net shrinkage [19–21] with results shown in Fig 5. The central region of the 200 RPM deposition registers the highest porosity–~92%–observed in this study and is bounded by approximately symmetric decreases to ~89% at both edges. The 1100 RPM deposition displays an almost uniform porosity throughout at ~88–89%. For 2000 RPM, the lowest porosity of ~87% occurred in the middle and rebounds to ~89% toward the edges. Additionally, “local fluctuations” in porosity are also notably higher in the central region (70<Z<120 mm) of 200 RPM deposition where “fiber arches” (Fig 3) are prevalent.

**Fig 6:**
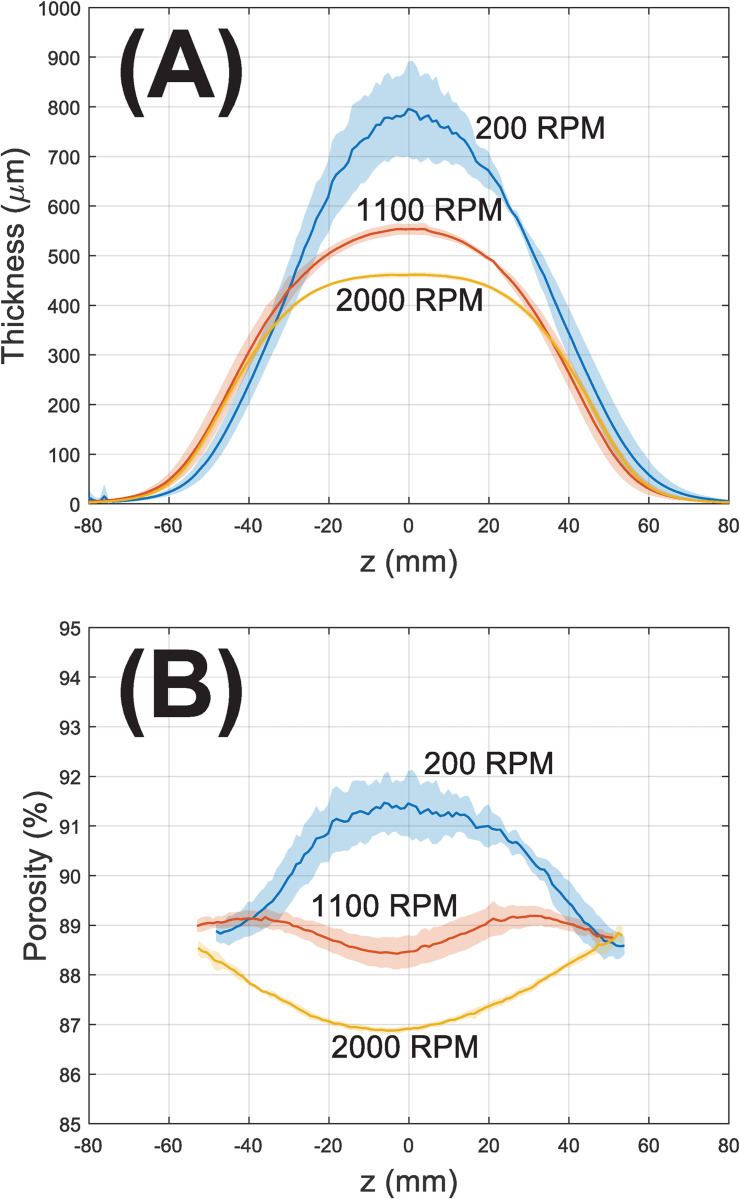
2D representation summarizing the (A) thickness and (B) porosity gradients along the axial direction (z) following depositions at 200, 1100, and 2000 RPM. The solid line represents the mean, and the shaded area represents the range of ± one standard deviations across different depositions (200 and 2000 RPM: n = 4; 1100 RPM: n = 3. See S1 File for details). To emphasize symmetry, the axial coordinates are centered with the as-deposited peak as the origin (z = 0). Thickness and porosity profiles of each individual depositions are available in S2 Fig in S1 File.

While small variations in thickness and porosity are present along the azimuthal direction, especially in the middle of 200 RPM depositions, both values are in large part solely dependent on axial coordinates. As such, only one deposition from each condition is chosen for 3D illustration, and the axial trend derived from all depositions are plotted in 2D (Fig 6). The solid lines represent arithmetic mean across 4 (or 3 for 1100 RPM, see S1 File) individual depositions while the shaded area denotes the standard deviation reflecting rod-to-rod variabilities.

As Fig 6A makes clear, the 200 RPM thickness follows an approximately gaussian distribution showing increased variability around the peak (~800 μm). At 1100 RPM, thickness deviates from gaussian with some flattening around the peak (~550 μm). At 2000 RPM, the flattening is more significant and reduces peak thickness to ~460 μm. Moving away from the peak, these profiles become almost identical on both sides.

Similarly, the porosity profiles (Fig 6B) display sensitivity to RPM mainly within the same central region (~-40<z<40 mm). The 200 RPM porosity profile is *concave-down* where the highest value (~92%) is co-located with the deposition peak (z = 0) and bounded by decreases to ~89% at both edges. At 1100 RPM, porosity remains almost constant (~88–89%) along all axial positions. The porosity profile of 2000 RPM is *concave-up* and the deposition peak (z = 0) has the lowest porosity (~87%) that rebounds to ~89% at both edges. Notably, the porosity difference between different RPMs is far more pronounced near deposition peaks (z = 0) than the averages (Table 1) suggest.

**Fig 7:**
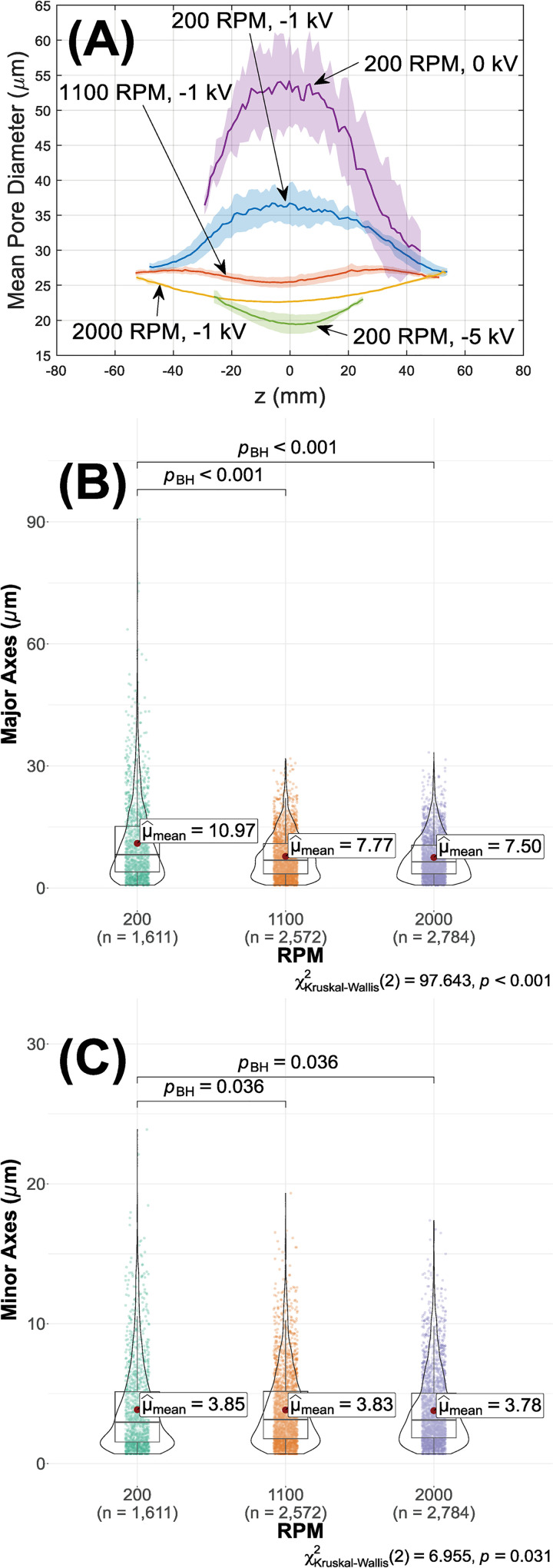
(A) Distribution of pore sizes versus axial positions (z) predicted by Eq (3) with laser metrology-based porosity and fiber diameters. Variations in fiber diameters against positions are neglected, and only values measured in the centers are used. As these variations are small (Table 1) and pore size only scales linearly with fiber diameter in Eq (3), this does not fundamentally affect the results. The solid line represents the mean, and the shaded area represents the range of ± one standard deviations across different depositions. To emphasize symmetry, the axial coordinates are centered with the as-deposited peak as the origin (z = 0). Data for different collector biases (-5 and 0 kV) are derived from our prior work [19]. (B) Major and (C) minor axes of pores measured from deposition center under SEM following 200, 1100, and 2000 RPM depositions.

Employing Eq (3), variation in pore sizes against axial position predicted with fiber diameter and porosity data are depicted in Fig 7A. Pore sizes are ~26 μm on either end regardless of RPM, but clearly follow the ranked order 200 (~37 μm)>1100 (~25 μm)>2000 (~23 μm) RPM near deposition peaks. The same data for different collector biases derived from our prior work [19] is also included for comparison. The attractive bias clearly has a more pronounced influence: pore size dropped from ~54 (0 kV) to ~19 (-5 kV) μm in the middle of depositions. The distributions of major and minor pore axes directly measured under SEM are summarized in Fig 7B and 7C, respectively. Notably, 200 RPM produces significantly longer ‘tails’ of large pores, especially for the major axes.

**Fig 8:**
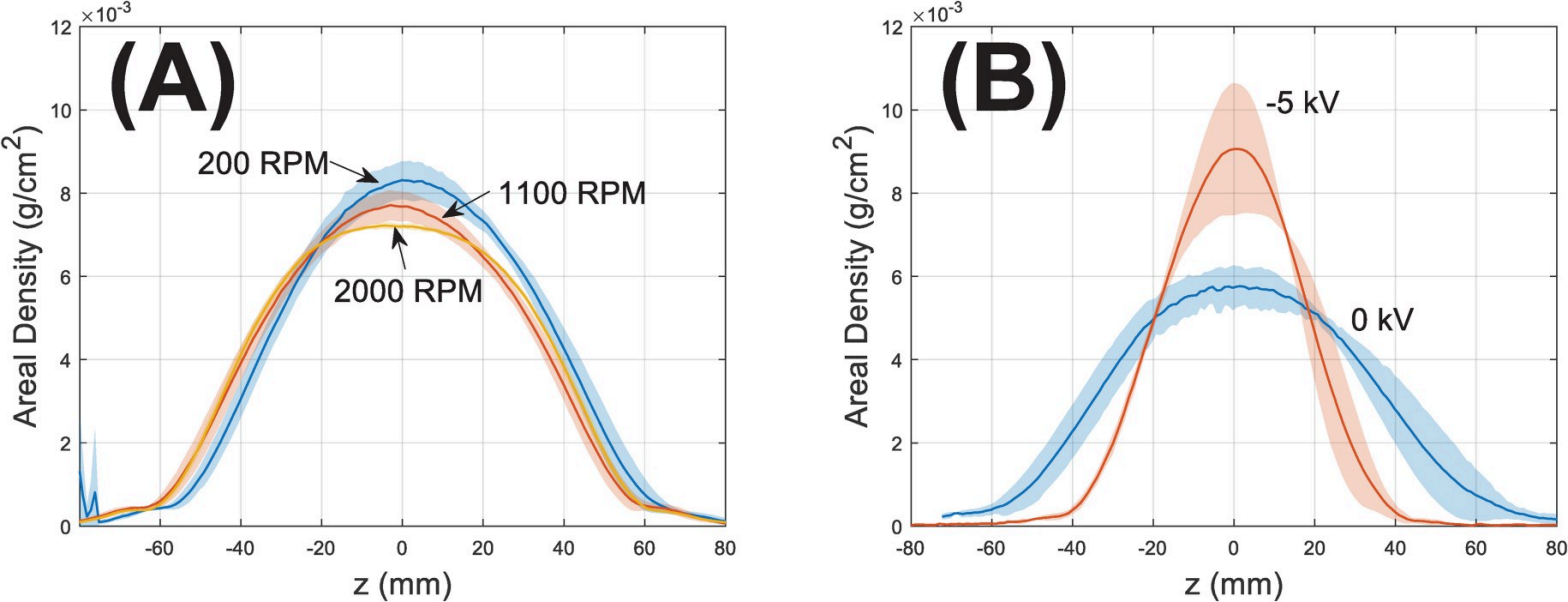
Effects of (A) RPM and (B) collector bias (data derived from our prior work [19]) on the mass distribution of depositions along axial (z) direction. Values are calculated from densified thickness and bulk PCL density of 1.145 g/cm3. The solid line represents the mean, and the shaded area represents the range of ± one standard deviations across different depositions. To emphasize symmetry, the axial coordinates are centered with the as-deposited peak as the origin (z = 0).

Fig 8A illustrates the axial variation in areal polymer density calculated from densified thickness and bulk PCL density. All three RPMs produce almost identical mass distributions despite minor variations near deposition peaks (z = 0). In contrast, enhancing collector biases [19] clearly shows a focusing effect (Fig 8B).

**Fig 9:**
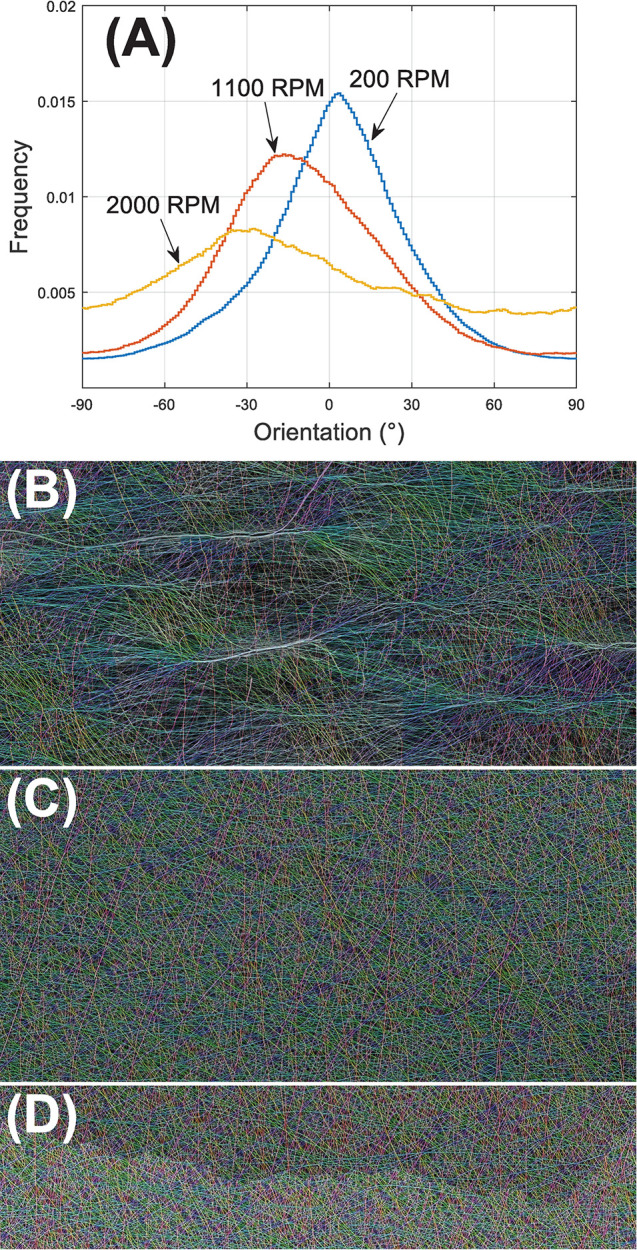
(A) Distribution of fiber orientations across axial (0°) to azimuthal (±90°) directions at different RPMs. Orientation-based colorization of SEM images taken from the center of (B) 200, (C) 1100, and (D) 2000 RPM depositions. To achieve better statistical representation, each image is stitched from 50 individual SEM images and covers a net area >2,000,000 μm2. In all three images (B-D), the direction of mandrel rotation (azimuthal, ±90°) is in the vertical direction. Original (un-colorized) images and colormap are available in S4 and S5 Figs in S1 File.

Finally, the observed fiber orientation–arguably the biggest driving force supporting fast collector rotation–is reported. The evolution of orientation distribution across axial (0°) to azimuthal (±90°) directions versus RPM is plotted in Fig 9A. At 200 RPM, the predominant orientation is along the axial direction and orthogonal to the direction of rotation. As the RPM increases, variance of orientation increases substantially, while the mean shifts away from 0°. Little preferential orientation is observed at 2000 RPM. To better visualize this, orientation-based color-mapping is shown in Fig 9B (200 RPM), 9C (1100 RPM), and 9D (2000 RPM).

## Discussion

Control of pore size is critical to functional tissue engineering. If too small, cells cannot migrate inwards regardless of whatever favorable mechanical or biochemical properties the scaffold possesses [1, 2]. Conversely, the integration/degradation characteristics of these scaffolds are also controlled by the available porosity and pore sizes [32–35]. Thus, considerable efforts have been directed toward establishing the desirable modifications of scaffold porosity [34, 35]. Toward this end, electrospinning stands out from various scaffold fabrication technologies as numerous process variables [1, 2, 19, 20, 36–38] as well as post-modifications [1–5, 39, 40] are available for porosity tuning. While the relationship between the ‘ideal’ pore size of any scaffold and biological integration is controversial and application-specific [32–35], we can work with the results of this investigation and adapt established statistical models to predict pore sizes based on the observed porosity data. Comparing these sizes to what is currently believed to be useful then serves as a benchmark regarding the results of this study and others [19, 20].

In terms of thickness, both the visual (Fig 1) and laser data (Figs 2 and 6A) lead to similar conclusions. Considerable roughness is observed at 200 RPM while visually eliminated at higher RPMs. Increasing RPM decreased thickness almost by half and flattened the central region, implying porosity alterations.

Notably, the spread of depositions remains constant (~105 mm, Table 1) regardless of RPM. In contrast, in our prior work [19] a -5 kV collector bias results in an ~37% decrease in the deposition span (61.5±5.7 mm at 100 μm thickness) versus 0 kV (98.1±4.8 mm). Further, the mass distribution (Fig 8) is almost identical versus RPM while -5 kV created a much more focused deposition than ground (0 kV). This aligns with the fact that applied bias exerts influence over the entire electrospun jet via electric fields while mechanical action of collector rotation is mainly local. It has been observed in simulations [19, 41] that applied bias modifies the size and shape of the bending cone [42, 43], which corresponds to a narrower deposition. It is also conceivable that enhanced bias may also indirectly control spread via affecting charge decay following deposition. However, considering their relative strength the modification by residual charges to externally applied fields should be minor and this may be only a secondary factor in determining spread.

That the thickness drops off substantially at the edges of depositions is in accordance with our prior observations [19, 20]. SEM (Fig 4) reveals distinct qualitative differences between fibers in the center and those at the very edge. In all three RPMs, the presence of ‘curly’ fibers at edges indicates buckling instability [31] at jet impingement on the hard metal surface. Buckling is reduced in the central regions where the collector is covered by a soft layer of prior depositions. Visually, the porosity of 200 RPM central deposition is higher than any other RPM/location, in line with the sintering-based data (Fig 6B).

We also note that, at a practical level associated with mesoscopic tissue integration, the visually apparent roughness observed following 200 RPM depositions is resolved into periodic micro-arches of vertically stacked fibers (Fig 3). Similar microstructures have also been reported in our previous publications [19, 44] and we believe them to be reflections of certain (quasi-)periodic characteristics of electrospun jets. Simulations show comparable patterns in deposition densities due to the interaction between residual charge dynamics and the incoming jet. However, this aspect is not the focus of the current study. Nevertheless, in principle these features should enable far better mechanical stress transfer across the scaffold-host interface versus the relatively flat, low *Ra* depositions offered by 1100 or 2000 RPM. The availability of such a ‘forest’ of protrusions increases the prospects of rapid bio-mechanical integration of the construct surface by hard or soft tissues. The fact that these surface features also contain relatively small areas of highly aligned, cell impermeable microstructures may be outweighed by the ability of the remaining surface to promote larger scale integration.

Extensive characterization of fiber diameter (n>2,500) revealed statistically significant, yet numerically small (<0.26 μm) variations versus RPM and location. As the fastest linear speed (0.995 m/s at 2000 RPM) deployed here is only a fraction of typical terminal velocities occurred in electrospinning (~4 m/s [43, 45]), we do not expect mechanical drawing to fundamentally change fiber diameter. This suggests that the variations in fiber diameter present in this study will have far less impact on performance than porosity.

In contrast, porosity gradients (Figs 5 and 6B) exhibit marked sensitivity to rotational speed. Central porosity decreases from ~92% (200 RPM) to ~88% (1100 RPM) and ~87% (2000 RPM) while edge porosities are invariantly ~89%. The enhanced sensitivity at the center is a natural consequence of higher deposition rates (Fig 8A) which translate into relative abundance of residual solvent and electric charges in these regions. At 200 RPM residual charges provided enough inter-fiber repulsion to form higher central porosities than their surroundings, whilst at higher RPMs this was eventually overcome by mechanical compactions. The lack of residual solvents toward the edges makes relatively ‘dry’ depositions less susceptible to these forces characterized by unvarying porosities. A considerable rod-to-rod variability in porosity is observed at 200 RPM, smaller but detectable variance can be distinguished in the 1100 RPM data. The 2000 RPM depositions provide the lowest porosity but also the best rod-to-rod consistency. The *concave-up* character of porosity gradients in 1100 and 2000 RPM depositions suggests that–contrary to what might be expected–cell infiltration will be *more* efficient toward the edges and *less* efficient in the center.

Due to the limitation of our particular setup, we cannot reliably measure porosity in areas <~120 μm thick that was excluded from visualization. This threshold approximately coincides with the value used to determine deposition spread (Table 1). Regardless, in typical electrospinning fabrication removal of thin tapered edges [40] is widely practiced and porosity in these regions is likely of limited relevance.

Characterizations of fiber orientations (Fig 9) show that the increase in RPM resulted in a modest shift from axial toward azimuthal alignment. The influence of the cylindrical electric field and how it orients fibers in the axial direction has been previously described [19, 41]. Due to the small collector diameter (~0.95 cm) the maximum linear rotational speed achievable in this study (0.995 m/s at 2000 RPM) cannot match those previously used (~4–15 m/s [22, 25, 26]) to get far more effective azimuthal alignment. Even at 2000 RPM, only roughly random orientation is observed. However, clearly porosity is very sensitive to RPM (Fig 6B) despite limited shifts in orientation. We expect that higher rotational speeds would ultimately establish preferred orientation along the azimuthal direction but at the cost of an even greater suppression of porosity.

While there have been substantial driving forces toward nanofiber scaffolds due to their microstructural similarity to natural extracellular matrices, it should be noted that generally pore size also decreases in these scaffolds [36–38]. To illustrate that the single-digit variation of porosity (Fig 6B) corresponds to substantial changes in pore size and ergo the propensity toward cellular infiltration, we substitute porosity values into a statistical model to predict gradients of mean pore diameter along the axial direction (Fig 7A). The model [27] considers isotropic near-planar network of fibers following a Poisson line process whose probability depends on the mean frequency. The linear dependance of pore size $\bar{d}$ on fiber diameter *d*_*f*_ suggested by the model (Eq (3)) has also been reported in experimental studies [46]. On the other hand, according to the same equation $\bar{d}$ grows exponentially as porosity *p*→100%, therefore variation in *p* clearly dominates $\bar{d}$ in the current study.

Overall, the conversion of porosities to pore sizes versus conditions was quite revealing. At 200 RPM, the difference in pore size between 0 (ground) vs -1 kV on the mandrel is substantial (~54 vs ~37 μm). While the fact that enhanced bias modifies porosity is well-established [19, 20], it is still somewhat surprising that the difference of a single kilovolt would have such a significant effect, suggesting that tight levels of control might be achieved at fractions of a kilovolt. Further increase reverse bias to -5 kV reduces pore size to ~19 μm. Varying rotational speeds from 200 to 2000 RPM at -1 kV also reduces pore size from ~37 to ~23 μm. These results suggest that while fast rotation or reverse biases can promote alignment or deposition efficiency, they do so at the cost of potential restrictions on cellular infiltration.

SEM measurements confirmed a similar trend of ranked pore size 200>1100>2000 RPM (Fig 7B and 7C). These highly skewed pore size distributions are consistent with those observed in similar structures [47, 48]. However, the average is only about one fifth of the model prediction (Fig 7A). This discrepancy may be attributable to two factors. First and foremost, image segmentation was inefficient in differentiating different planes within the depth of field which has certainly led to underestimation of pore sizes. On the other hand, the Sampson model [27] is based on isotropic random network(s) of uniform fibers, and the presence of preferred orientation and fiber diameter variance in our experiments clearly deviates from the ideal model. Nevertheless, the shift in major axes versus RPM is far more pronounced than in minor ones, in accordance with the diminishing preferred orientation at increased RPM (Fig 9).

**Fig 10:**
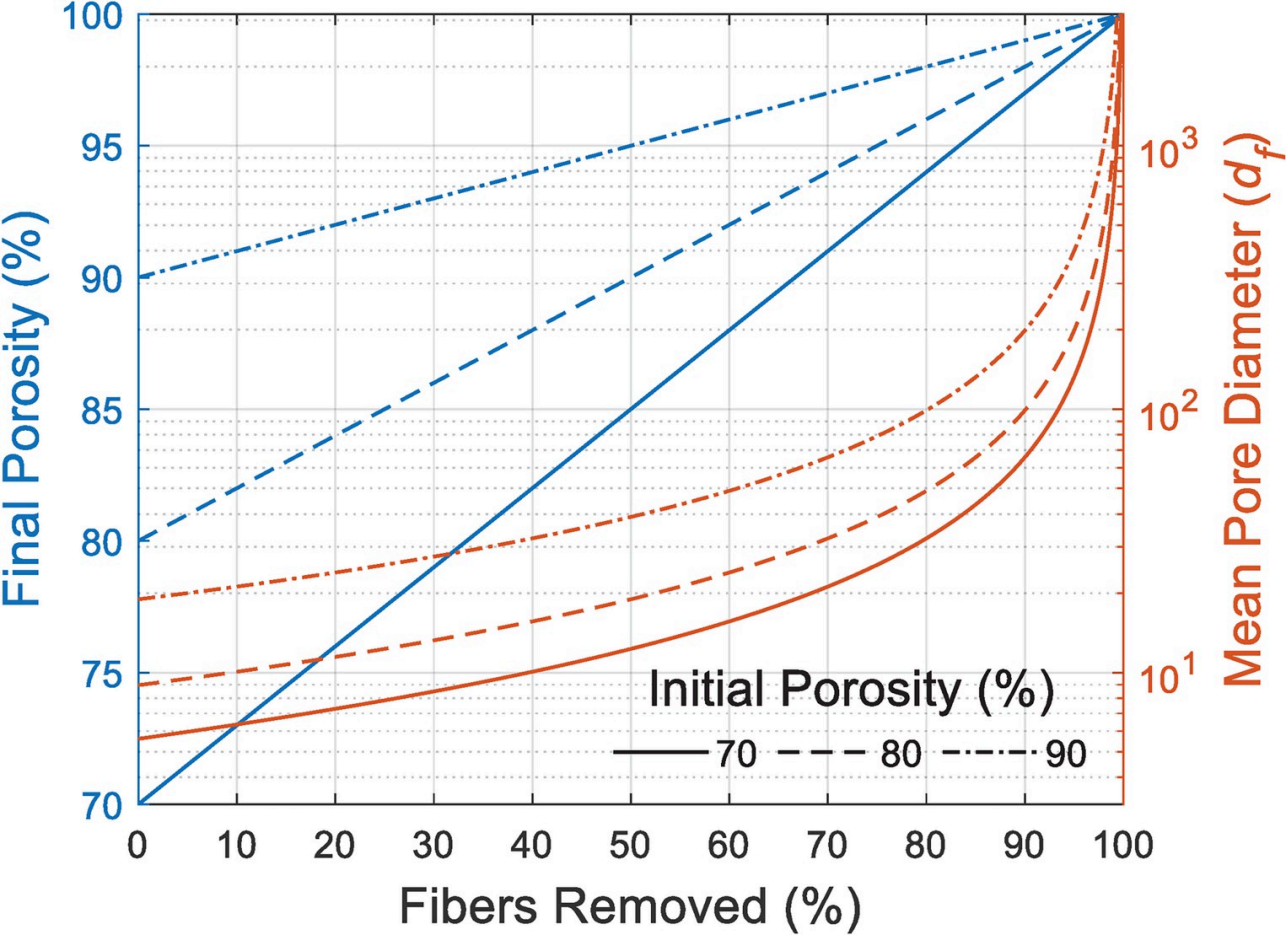
Projections of final porosity (left-hand y-axis, straight lines) and pore size (right-hand y-axis, predicted by Eq (3), curved lines) versus removal of sacrificial fiber from initial scaffolds having the porosities shown in the legend. The pore size reported is rendered dimensionless as ratio over fiber diameter.

The use of sacrificial fiber spun within matrix of more permanent fiber is often examined as a means of improving cellular infiltration [1, 2, 5, 6]. The pore prediction model also allows us to estimate the efficiency of this strategy in enlarging pore size, assuming that both types of fibers share the same diameter, and their distributions are uniformly random while uncorrelated. Fig 10 shows that at three different initial levels of porosity, as the percentage of removed fiber increases, pore diameter increases as expected but only to relatively modest levels. For 98% porous (e.g., 90% initial porosity followed by 80% fiber removal), randomly oriented fibers, the mean pore size is only ~100× of fiber diameter, e.g., ~100 μm pores with ~1μm fibers or ~10 μm pores with ~100 nm fibers. Even if we ignore the potential contraction [39] by capillary or gravitational actions on such high porosity scaffolds, this suggests that breaking random distribution of fibers, via use of either isolated porogens [1–4] or the exquisite geometric capabilities of laser ablation [49, 50], would be more efficient at enhancing cellular infiltration.

It should be noted that discussion on pore sizes until now mainly pertains to the number-averages, while electrospinning typically creates hierarchical pores spanning a wide range of sizes (e.g., Fig 7B and 7C). This contrasts with many cellular infiltration studies [51–53] that utilize more uniform pore sizes. We propose that electrospun scaffolds may still be able to accommodate efficient cellular infiltration by simply possessing a critical volume fraction of large pores. The mean pore size may remain relatively small due to the presence of many smaller pores, which can be useful in other aspects of tissue engineering such as protein adsorption [35, 54, 55].

Nevertheless, while such critical fraction is still to be exactly determined, one important aspect will be that the ‘pathway’ connecting these large pores should also be large enough to allow continuous cell migration. This is analogous to the concept of “pore throat” in study of filtration and permeability expect that connectivity is only required from either side of the scaffold. For example, theoretical estimation of mean “pore height” was found to be around half of in-plane diameter in highly porous, multi-layered random network of circular fibers [56] and more likely to bottleneck infiltration. However, in this context comprehensive 3D modeling of scaffold microstructure may prove to be more relevant than considering stacks of multiple planar networks. In particular, we note that the accessible pore volume for a particular spherical element (e.g., a cell) can be approximated via a series of morphological erosion and topological analysis inside 3D virtual scaffolds [17, 48]. Additionally, simulation of fluid flow versus medium directionality has also been carried out [57], which can be useful in the study of fiber orientation-guided cell migration.

It is important to note that spatial variations in porosity and microstructures found within a single electrospun deposit can sometimes overshadow the influence of deliberately controlled process parameters. For example, in the current study 200 RPM depositions can have pore sizes identical to or larger than 2000 RPM depositions depending on the location (Figs 4 and 7A). Other parameters such as bias, solvent identity, and environmental humidity are also known [19, 20, 30] to modify not only porosity averages, but also its spatial gradients. Given the central role of residual solvents and electric charges in forming porosity gradients, it would be very interesting to see how they accumulate and affect microstructure evolution in time. The effects of air flow and electrical resistance would also make attractive candidates for future investigations. Nonetheless, our work demonstrates that for electrospinning process development it is paramount to prepare samples from multiple regions of a larger deposit to fully capture the entire range of characteristics, especially when limited field-of-view is available to a certain characterization.

One can logically speculate that the internal gradients reported here will grow more complex in multi-needle or needle-less electrospinning. These setups have been extensively studied [40, 58, 59] as scale-up strategies, but their effects on microstructural gradients remain largely unexplored. Our prior work revealed that a delicate interplay exists between individual fiber jets that can create inhomogeneous contribution to the deposit [20]. Paired with exacerbated solvent retention, the resulting heterogeneity could become quite sophisticated. Translation of either the needle or the collector during deposition does present an opportunity to mitigate these effects, although the precise impact of the frequency and character of these motions on porosity and property development constitute a ‘rich’ area of research.

Finally, it is worthy to note that Eq (1) applies only to specimens whose vertical dimension is significantly smaller than horizontal ones–i.e., thin films–so that densification occurs mainly in this direction [30]. Extending the same methodology–determining porosity from shrinkage–to more ‘3D’ structures will necessity more comprehensive measurement and modeling of the sintering process. On the other hand, our method should be compatible with arbitrary membranes regardless of fabrication techniques or compositions, so long as they can be fully densified. Replacing furnaces with online sintering (e.g., high-power lasers) may also enable real-time porosity sampling of selective regions, although certain non-destructive electrical/optical measurements of deposition density may prove to be more efficient.

## Conclusions

A new method of scaffold characterization, laser metrology, was found to provide a substantial improvement in our ability to visualize the spatial gradients of porosity and pore size in electrospun depositions. Among the three rotational speeds investigated, considerable sensitivity of both properties was observed in the central region of electrospun PCL depositions, while generally invariant near the edges. This reflects the variability in residual solvent accumulation/removal across these regions. A 38% decrease in pore size (~37 to ~23 μm) was recorded after a modest increase from 200 to 2000 RPM (0.099 to 0.995 m/s) despite negligible fiber alignment, underlining the need to balance desirable mechanical/morphological properties against cell-permissive porosity in practical scaffold designs.

Our finding again highlights electrospinning’s exquisite sensitivity to process conditions such as bias or rotational speeds. Current procedures in which little standardization of these parameters exist clearly contribute to the widely variable results in which progress toward useful electrospun products is greatly hampered. On the other hand, making intelligent use of this sensitivity–feedback-controlled, real-time changes in process variables–holds the potential for ‘ideal’ scaffolds combining desirable bio-mechanical properties with porosity gradients optimized for cellular infiltration.

## Supporting information

S1 File (PDF)

S1 Data (GZ)
