# Supplementary material for: Visualization of porosity and pore size gradients in electrospun scaffolds using laser metrology
Source: PLoS One. 2023 Mar 9;18(3):e0282903. doi: 10.1371/journal.pone.0282903 (PMC9997878; doi:10.1371/journal.pone.0282903)
Supplement: S1 File — (PDF) [file pone.0282903.s001.pdf]

Supplemental Information:

Materials and Methods

Solution Preparation

A solution of 5 wt% PCL (Sigma-Aldrich, St. Louis, MO, M_n = 80,000 g/mol) in 1,1,1,3,3,3-hexafluoroisopropanol (HFIP) (Oakwood Chemicals, West Columbia, SC) was prepared in a 125 mL Erlenmeyer flask and mechanically mixed at 200 RPM with a 4 cm long x 8 mm diameter stir bar at room temperature (~20°C) for 8 hours. Once PCL was fully dissolved, well mixed, and completely homogeneous, it was transferred to a 60 cm^3 plastic syringe (BD Luer-Lok, Franklin Lakes, NJ).

Solution Characterization

The solution was characterized for solid content, surface tension and viscosity. Solid content was analyzed using a moisture analyzer (MB27, Ohaus, Parsippany, NJ) at a temperature of 58°C with a sample size of 3 g (n = 3). Surface tension was characterized at room temperature (~20°C) using a Theta Lite optical tensiometer (Biolin Scientific, Gothenburg, Sweden). The analysis was done using a pendant drop of the solution and in triplicate with a 22 Ga x 12.7 mm in length blunt needle (EFD, East Providence, RI). The surface tension was recorded at 1.7 frames per second for at least 12 s using OneAttension software (v4.0.5). Viscosity was determined with a ViscoQC™ 300 Type L (Anton-Paar, Ashland, VA) equipped with a low volume cup using a CC12 spindle, measured at a constant temperature of 23°C with a PTD 80 Peltier temperature holder and shear rate of 2.0 s^{-1} (n=3). Results of these measurements are reported below.

solid content	5.24±0.06 wt%
surface tension	17.68 mN/m
viscosity	846±8 cP

Preparation of electrospun scaffolds

The as-prepared PCL solution was processed within a Fluidnatek® LE-100 unit (Bioinicia-Fluidnatek S.L.U., Valencia, Spain) equipped with an environmental control unit (ECU) to tightly control air flow rate, temperature (T), relative humidity (RH) allowing batch-to-batch reproducibility. Electrospun fibers were deposited onto ~0.95 cm outside diameter (OD) and ~30 cm in length 316L stainless steel mandrels (McMaster-Carr, Catalog# 8936K5). Electrospinning processing parameters were as follows: the PCL solution was transferred through a 1.6 mm OD polytetrafluoroethylene (PTFE) tubing from the syringe to a 20 Ga x 12.7 mm in length blunt needle (EFD, East Providence, RI) at a flow rate of 6 mL/h. A needle-to-collector distance of 20 cm was used with an applied voltage in the needle and mandrel collector set to +10 kV and -1 kV, respectively. To maintain a clean environment during production, pre-conditioned air was filtered through a HEPA filter inside the ECU before entering the Fluidnatek LE-100 chamber for sample processing which was maintained at 25°C and 30% RH while removing evaporated solvents at an air flow rate of 80 m^3/h. Three independent rotating collector speeds were studied: 200, 1,100 and 2,000 RPM (linear speeds of 0.099, 0.547, and 0.995 m/s,

respectively). Electrospun PCL was collected under static conditions for 22 minutes in the central area of the mandrel (n=5, 4 for laser profiling and 1 for microstructural analysis).

One deposition from the 1100 RPM group displayed distinctly different characters (Figure S2) from the others and was excluded from this study. This may be attributable to the extra polish on the specific mandrel to make it fit into electrospinning apparatus. The resulting variation in contact resistances may have led to anomaly in deposition characteristics. Validation of this hypothesis is being explored but out of scope of the current study.

Scanning electron microscopy

Samples were placed on conductive carbon tape (TED Pella, Redding, CA) adhered to 1 cm diameter aluminum SEM mounts (TED Pella, Redding, CA) and sputter coated with 7 nm gold in air (Luxor, Nazareth, Belgium). Microstructure was then observed under a scanning electron microscope (SEM) (Thermo Scientific Phenom XL Desktop SEM) at accelerating voltages of 10 kV and at magnifications between 350× and 5,000×. Fiber bundles were imaged by placing the SEM mount at ~90° in the SEM chamber and using the previous imaging parameters. Fiber diameter (n > 2,500) was measured with the Fibermetric software package (v2.3.4.0) from four images (each RPM) at a magnification of 5,000×. 50 individual images were taken from deposition center at a magnification of 350× by scanning 10 columns and 5 rows. These were then compiled into stitched images using the Image Compositor Editor software (v2.0.3.0) using a structured panorama with camera motion set to "planar motion with skew." The "auto overlap" option was used to minimize possible imperfections in the final compiled image through all 50 tiles. Stitched images had a total area of ~ 3,300 x 1,550 µm, ~ 2,900 x 1,450 µm and ~ 3,400 x 870 µ, for the 200, 1,100 and 2,000 RPM depositions, respectively. Fiber orientation was analyzed from stitched images using OrientationJ (v2.0.5). Pore size was measured using Fiji/ImageJ (2.0.0-rc-69/1.52p) on SEM images (four 2,500× images for each RPM) after segmentation via DiameterJ. The major and minor axes of the 'best-fitting' ellipses of each polygonal pores are reported.

Figures

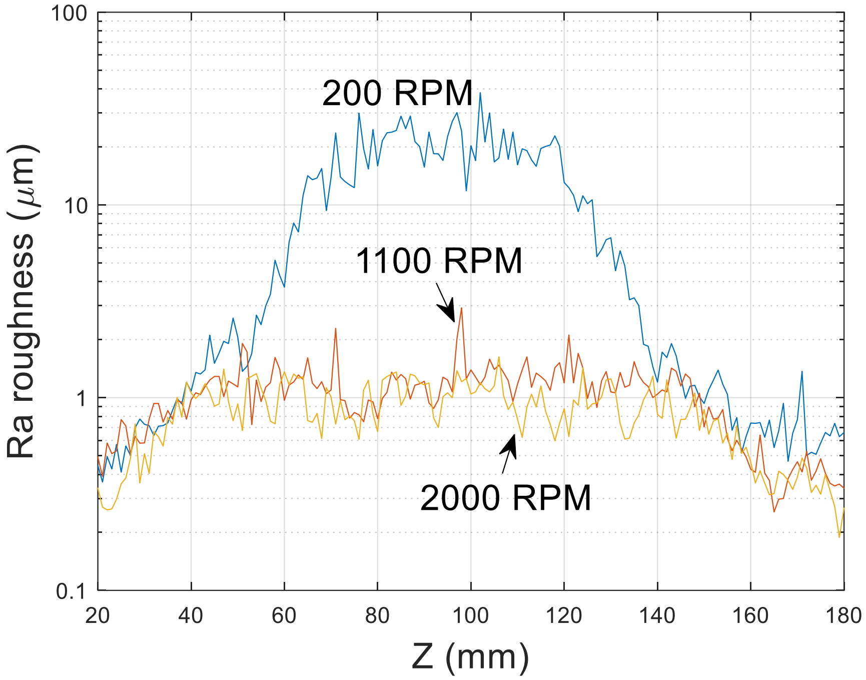

Figure S1. Roughness profiles of the depositions shown in Figure 2.

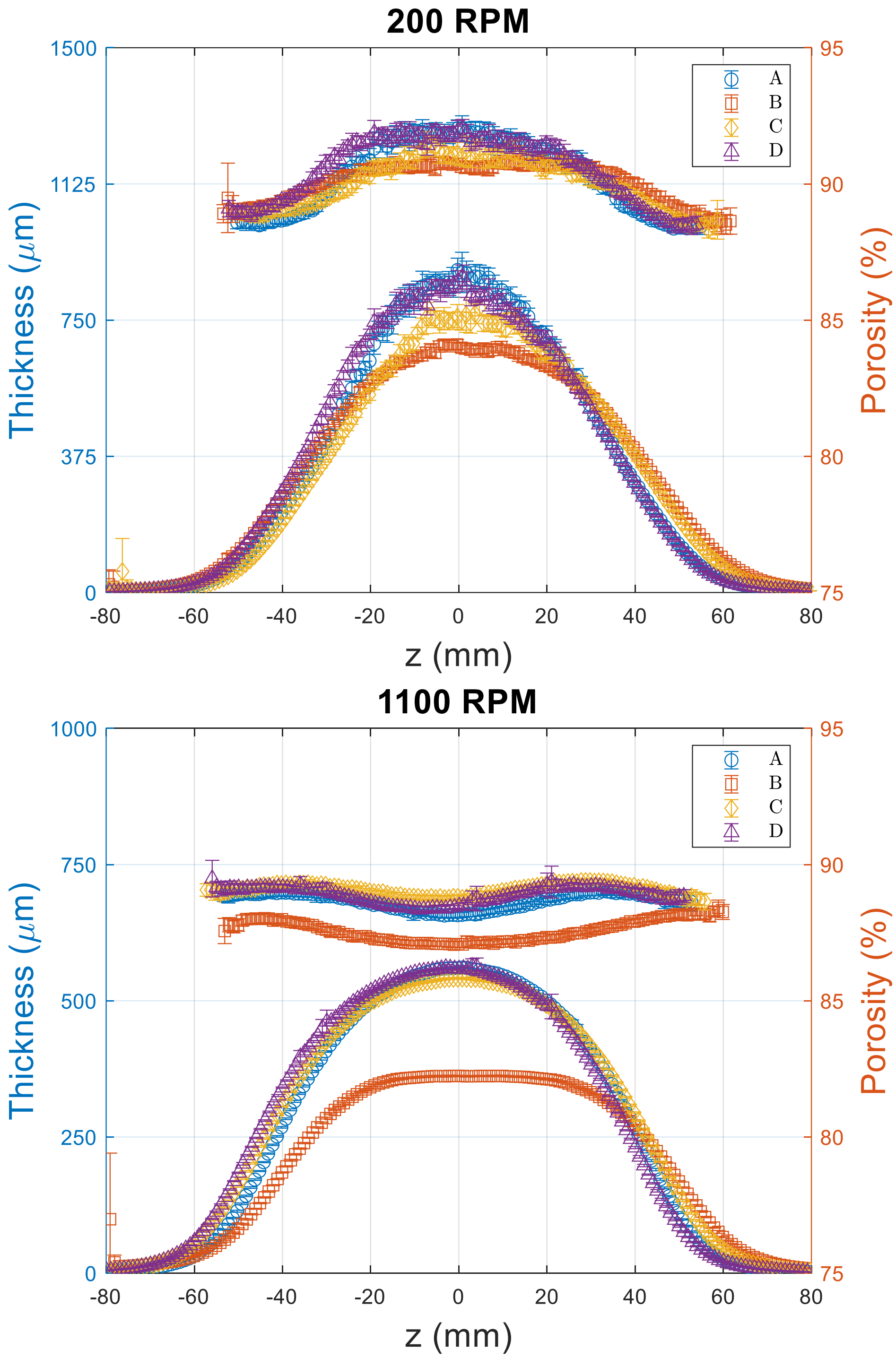

200 RPM
Thickness (μm)
Porosity (%)
z (mm)
A
B
C
D
1500
1125
750
375
0
95
90
85
80
75
-80
-60
-40
-20
0
20
40
60
80
1100 RPM
Thickness (μm)
Porosity (%)
z (mm)
A
B
C
D
1000
750
500
250
0
95
90
85
80
75
-80
-60
-40
-20
0
20
40
60
80

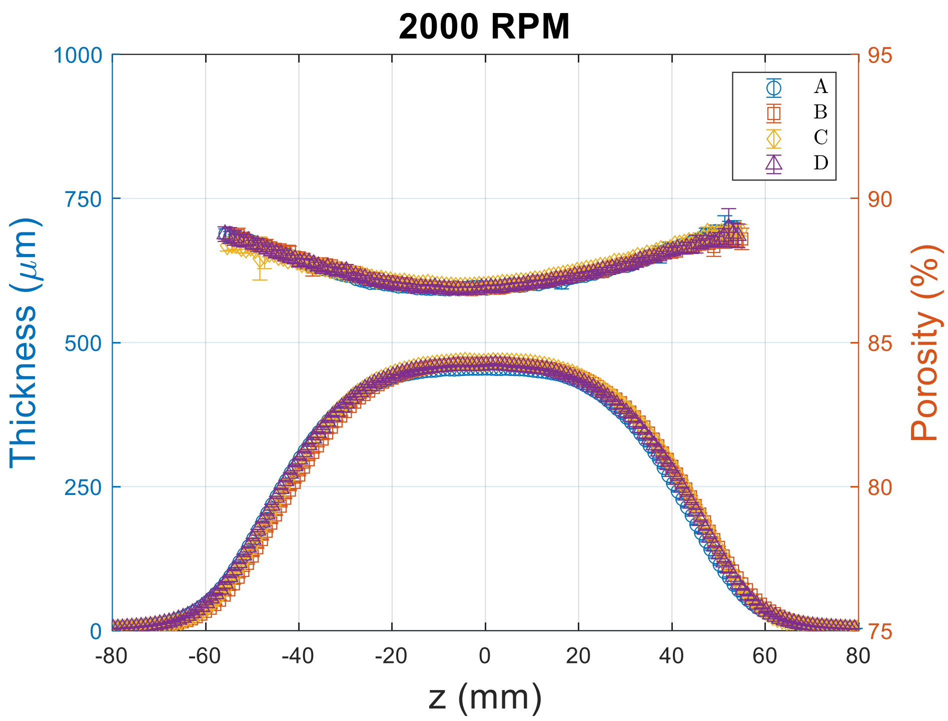

Figure S2. Thickness and porosity profiles of all depositions occurred in this study. Each datapoint and error bar represents mean ± one standard deviation across 72 thickness (or porosity) values at different azimuth/rotations for the corresponding axial position (z) from a single deposition. Sample B of the 1100 RPM group showed characters distinctly different from other depositions of the same group and is excluded from data reported in the results section. We believe this anomaly may be attributable to the extra polishing the associated collector rod has underwent.

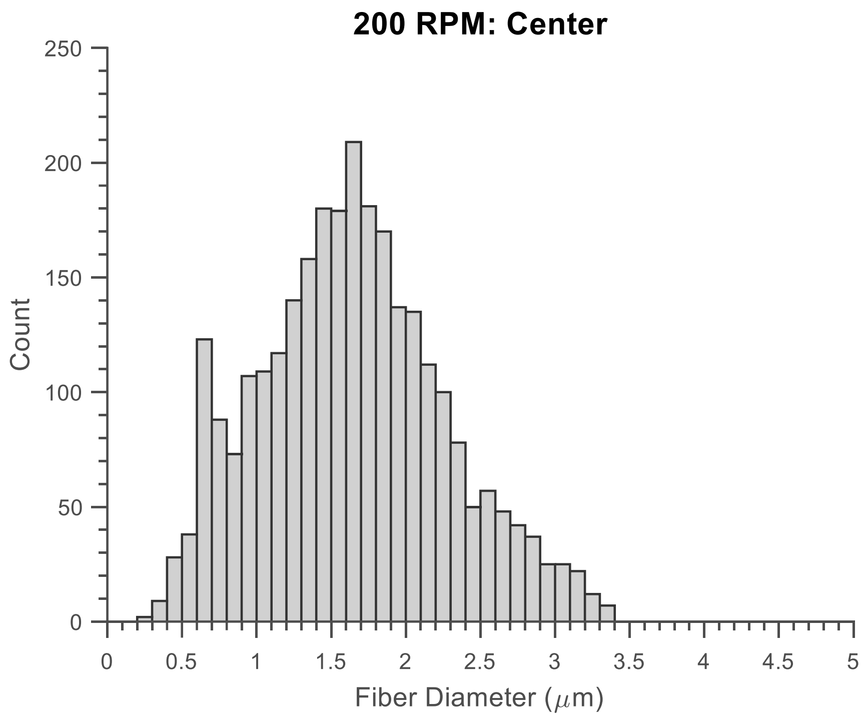

200 RPM: Center
Count
Fiber Diameter (μm)
0
50
100
150
200
250
0.5
1
1.5
2
2.5
3
3.5
4
4.5
5

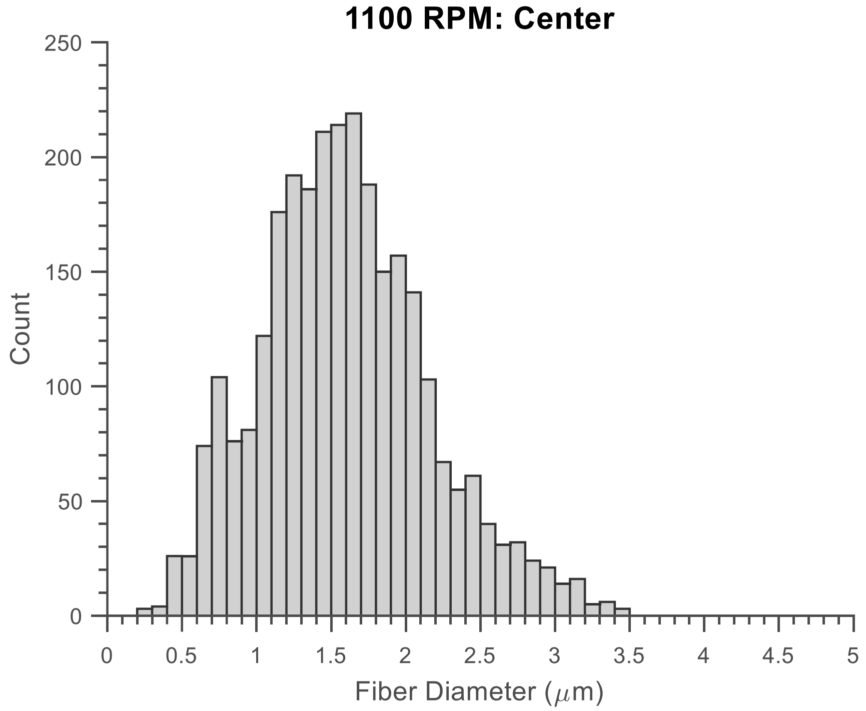

1100 RPM: Center
Count
Fiber Diameter (μm)
0
50
100
150
200
250
0.5
1
1.5
2
2.5
3
3.5
4
4.5
5

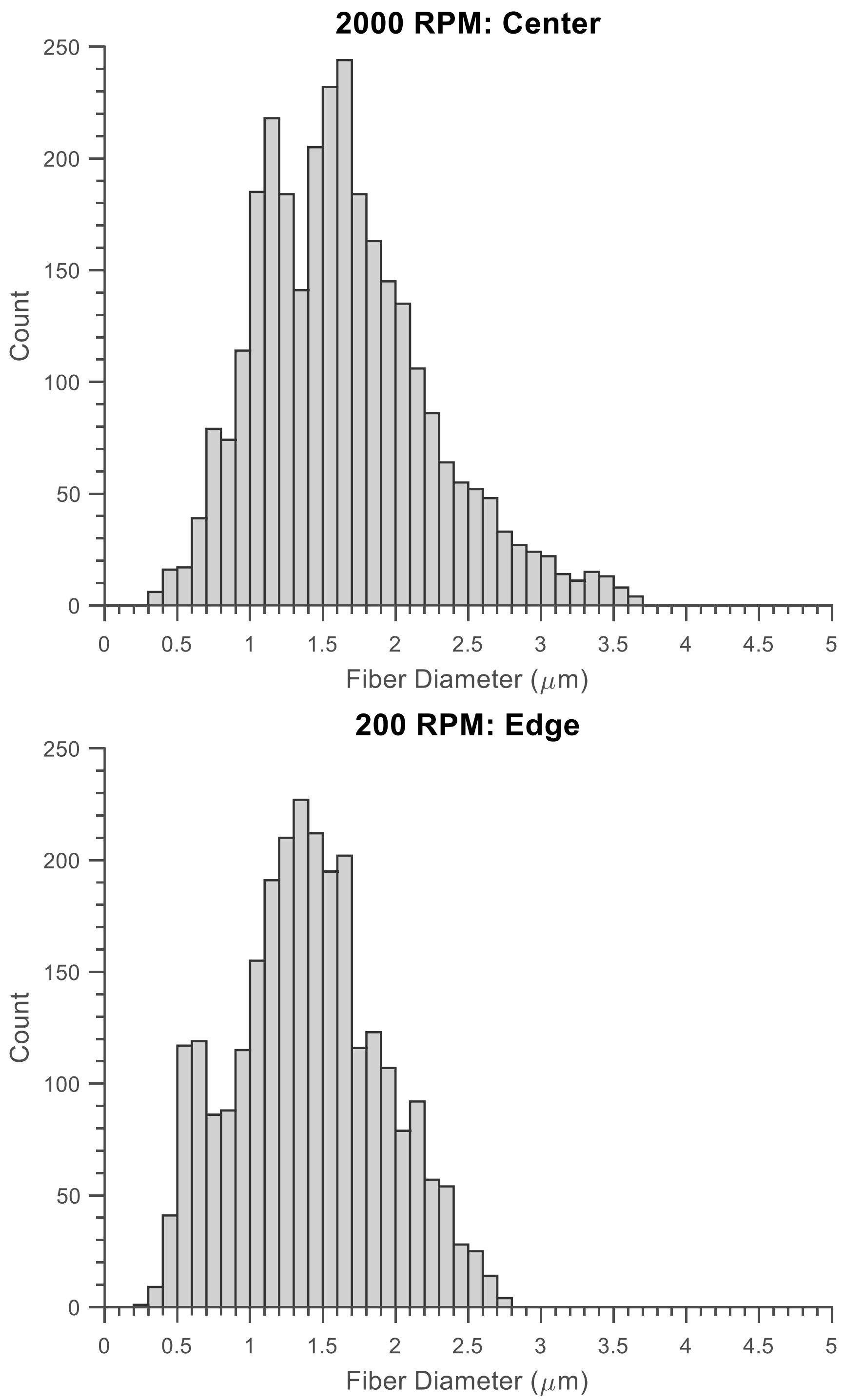
2000 RPM: Center
Count
Fiber Diameter (μm)
200 RPM: Edge
Count
Fiber Diameter (μm)

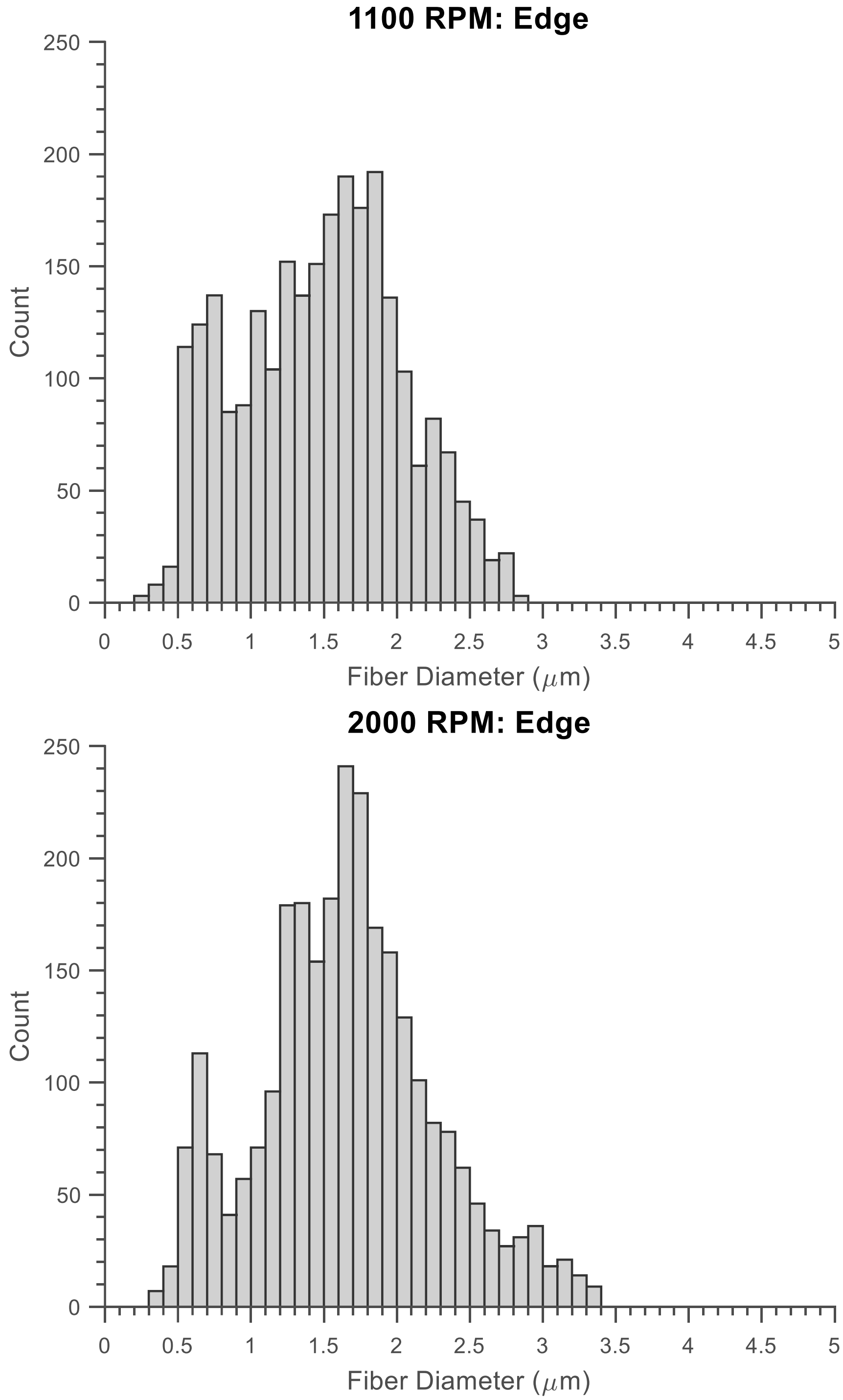

Figure S3. Histogram of fiber diameters for different RPM and locations.

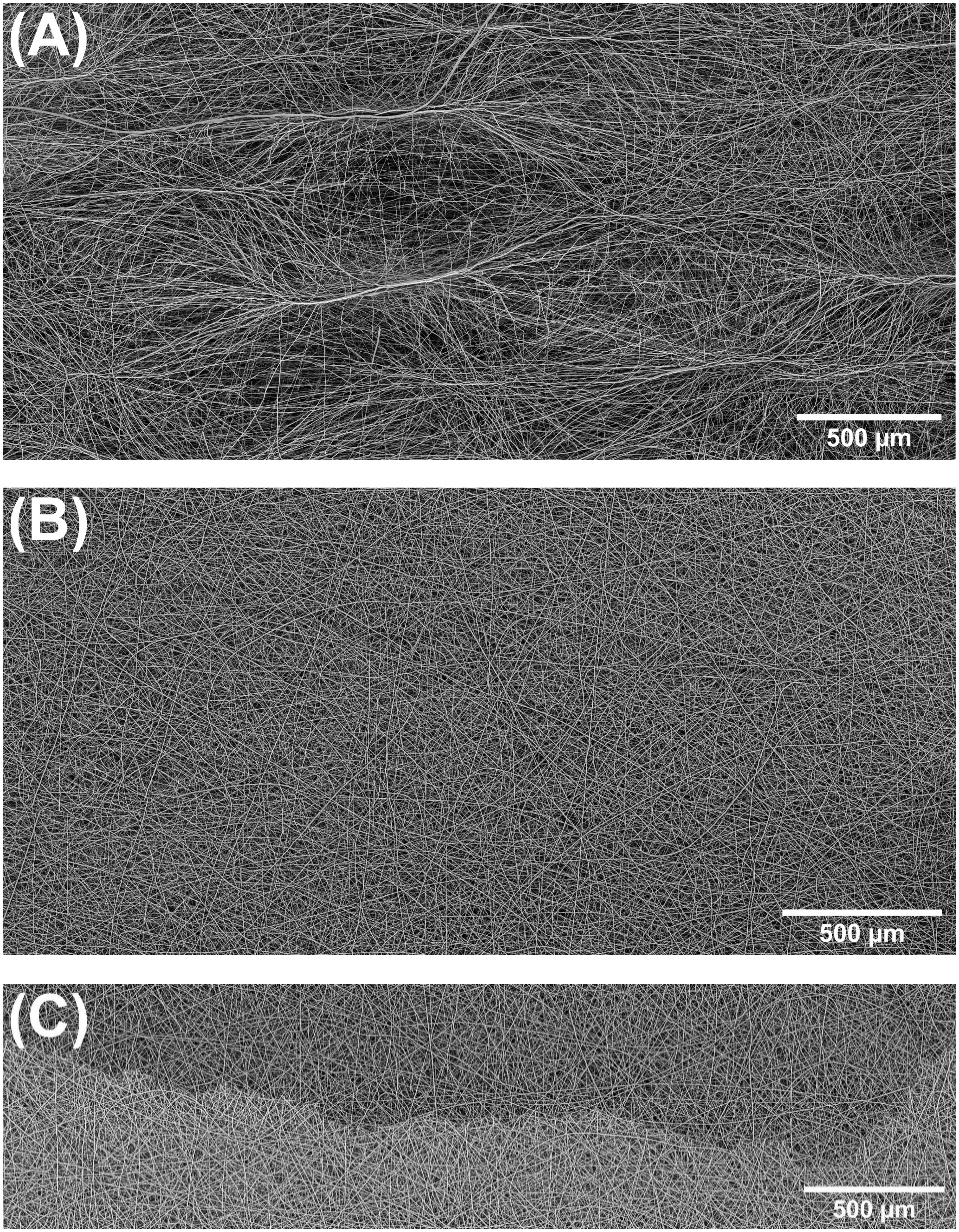

Figure S4. Stitched SEM images of (A) 200, (B) 1100, and (C) 2000 RPM depositions. The presence of aligned fiber bundles in 200 RPM is also visible.

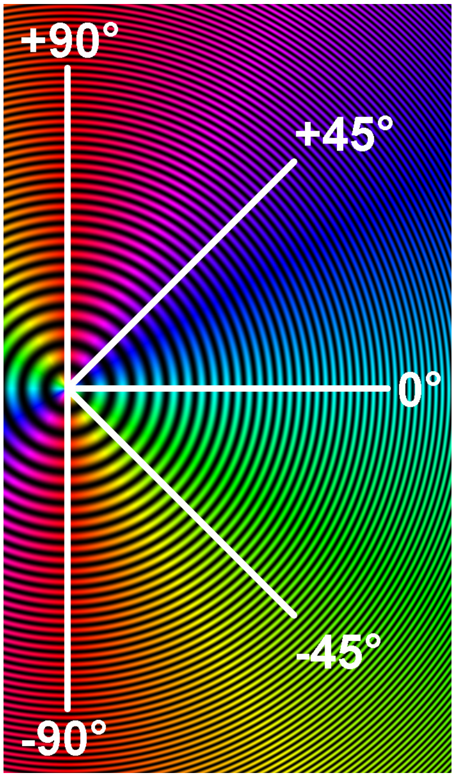

Figure S5. Colormap used in Figure 9B to D. 0° corresponds to the axial direction, and ±90° corresponds to the azimuthal direction of the mandrel collector.
