# Supplementary figures and images for: Visualization of porosity and pore size gradients in electrospun scaffolds using laser metrology
Source: PLoS One. 2023 Mar 9;18(3):e0282903. doi: 10.1371/journal.pone.0282903 (PMC9997878; doi:10.1371/journal.pone.0282903)

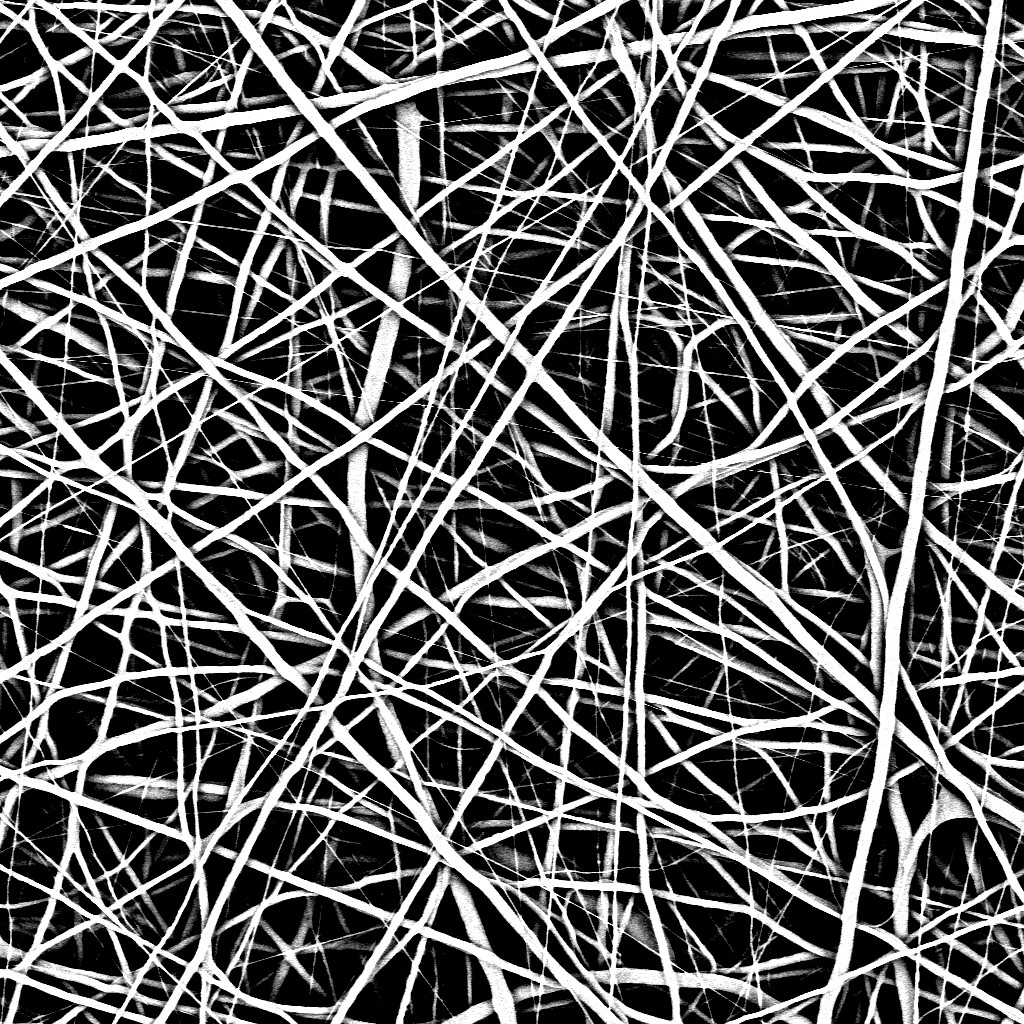

Supplement: S1 Data — (GZ) [file pone.0282903.s002.gz › data/PoreDiameter/RPM1100 (1).jpg]

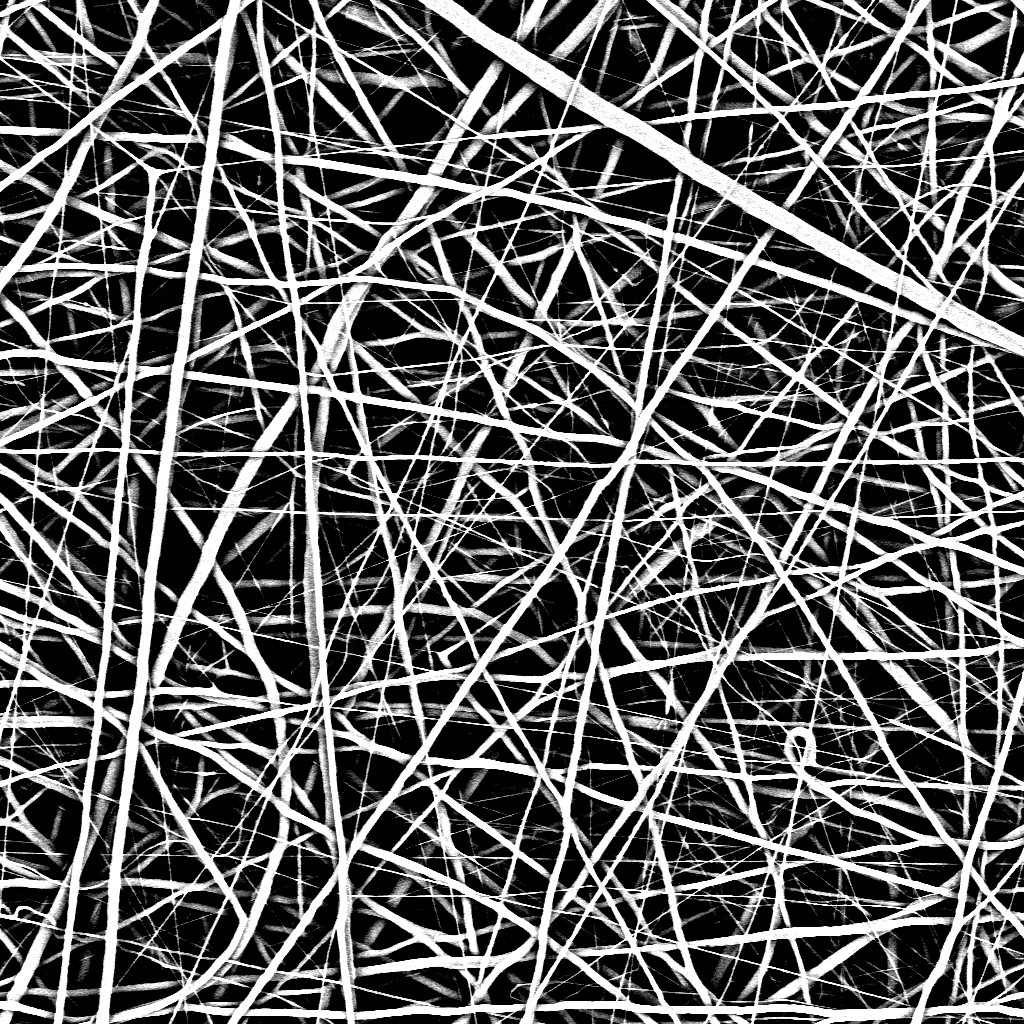

Supplement: S1 Data — (GZ) [file pone.0282903.s002.gz › data/PoreDiameter/RPM1100 (2).jpg]

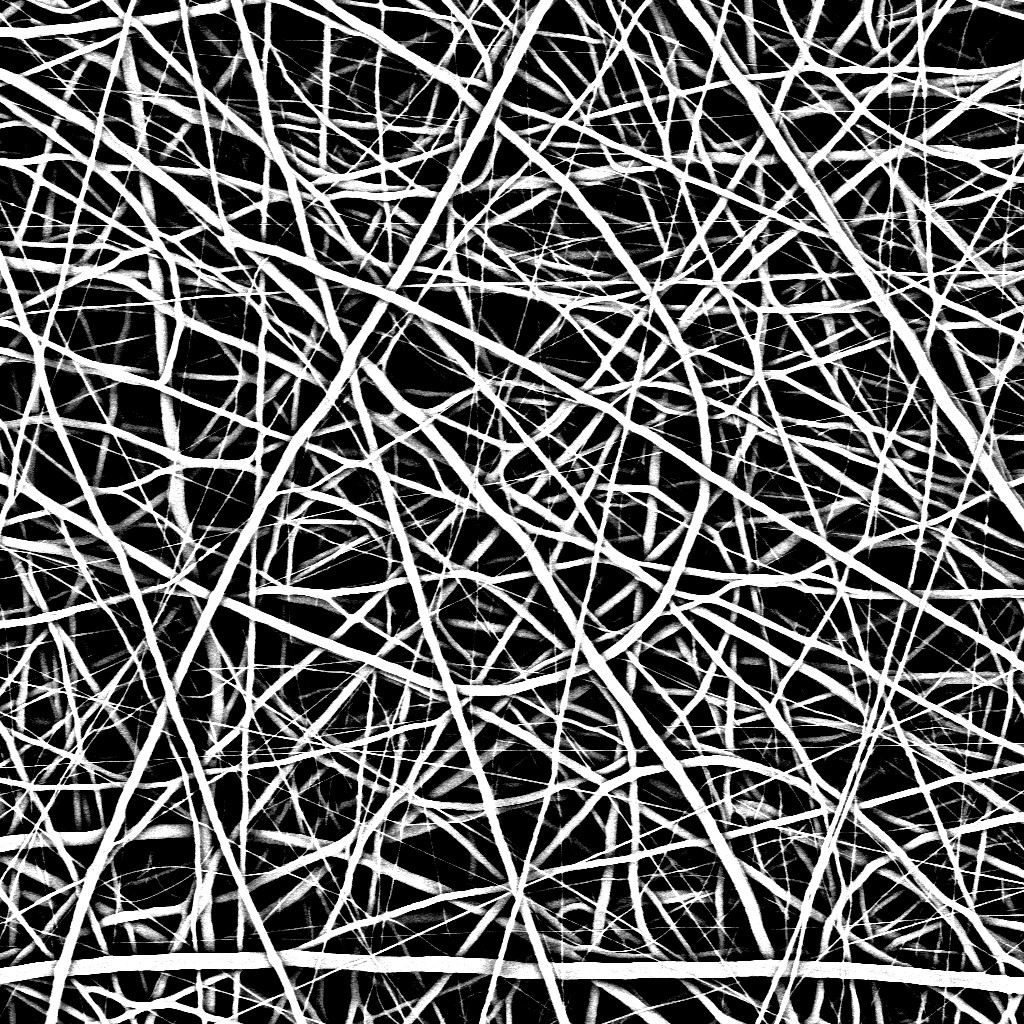

Supplement: S1 Data — (GZ) [file pone.0282903.s002.gz › data/PoreDiameter/RPM1100 (3).jpg]

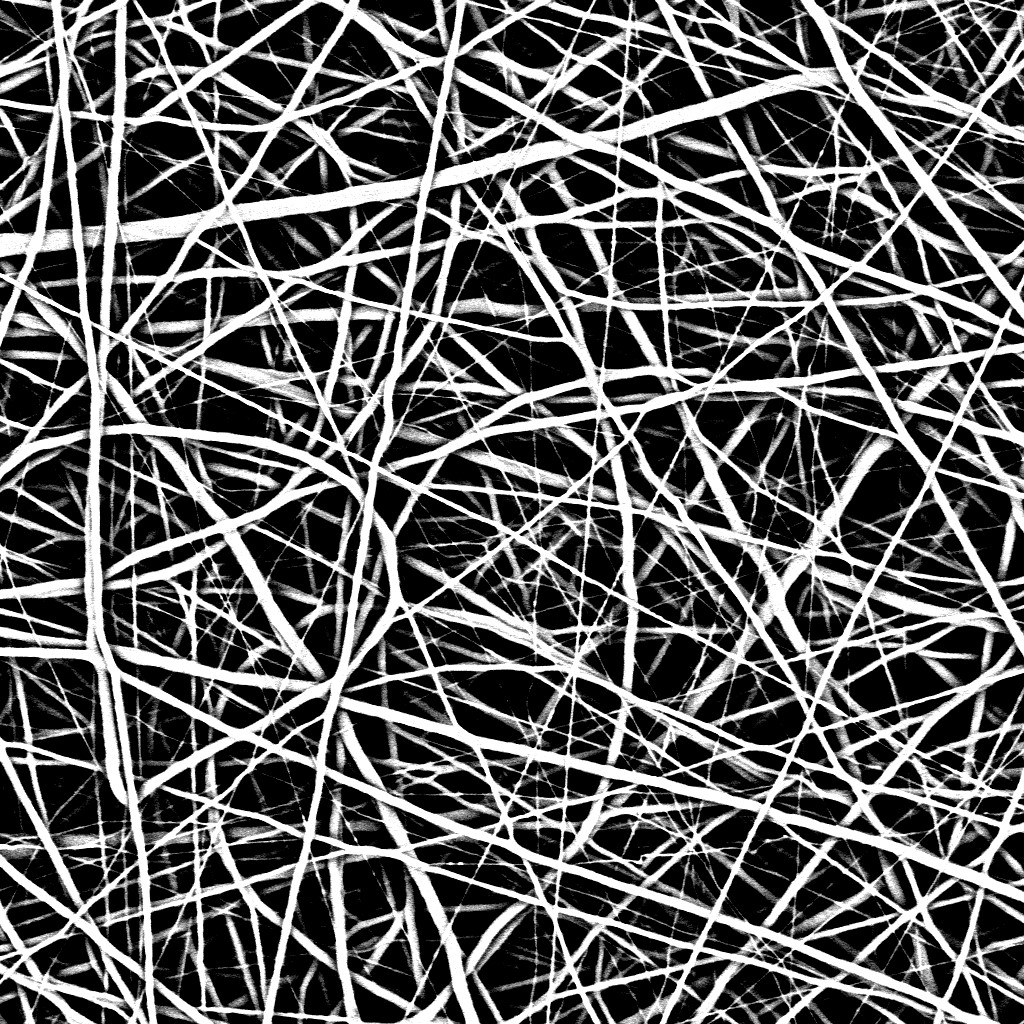

Supplement: S1 Data — (GZ) [file pone.0282903.s002.gz › data/PoreDiameter/RPM1100 (4).jpg]

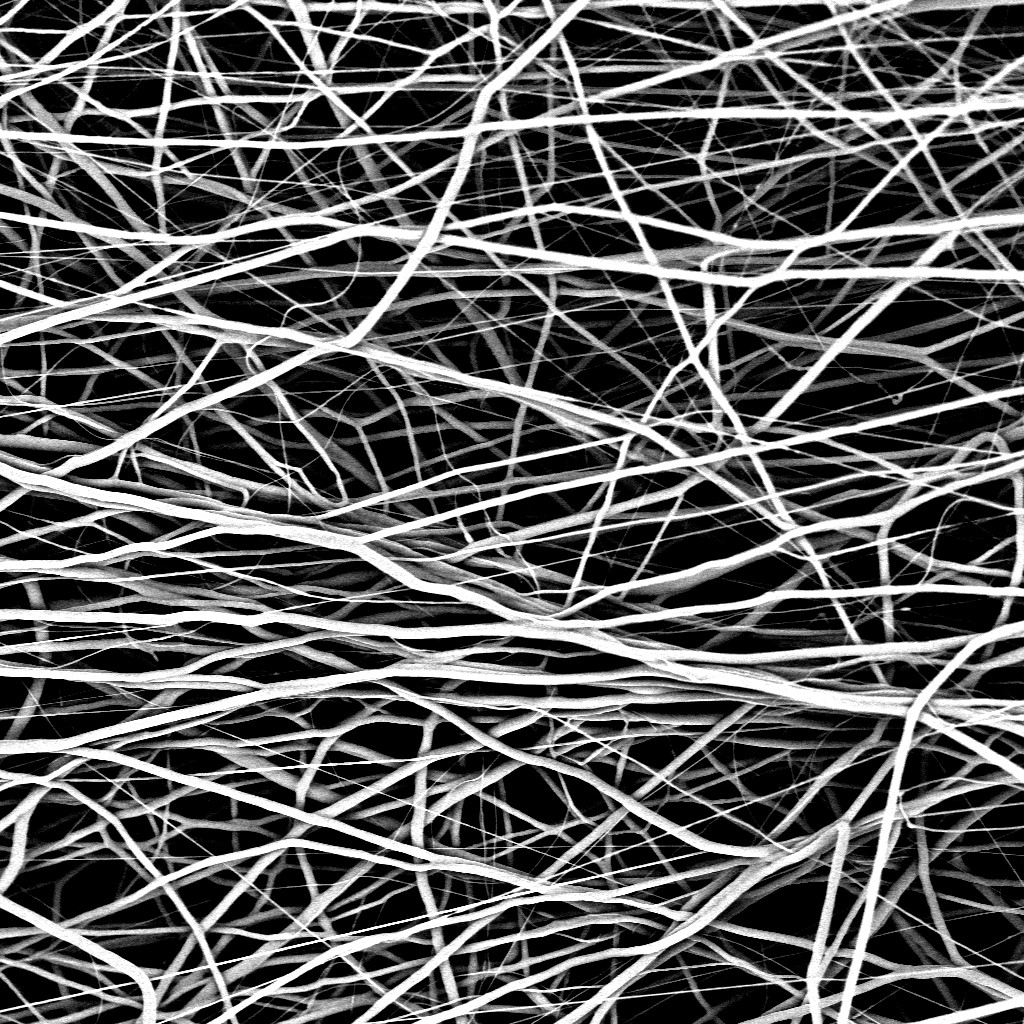

Supplement: S1 Data — (GZ) [file pone.0282903.s002.gz › data/PoreDiameter/RPM200 (1).jpg]

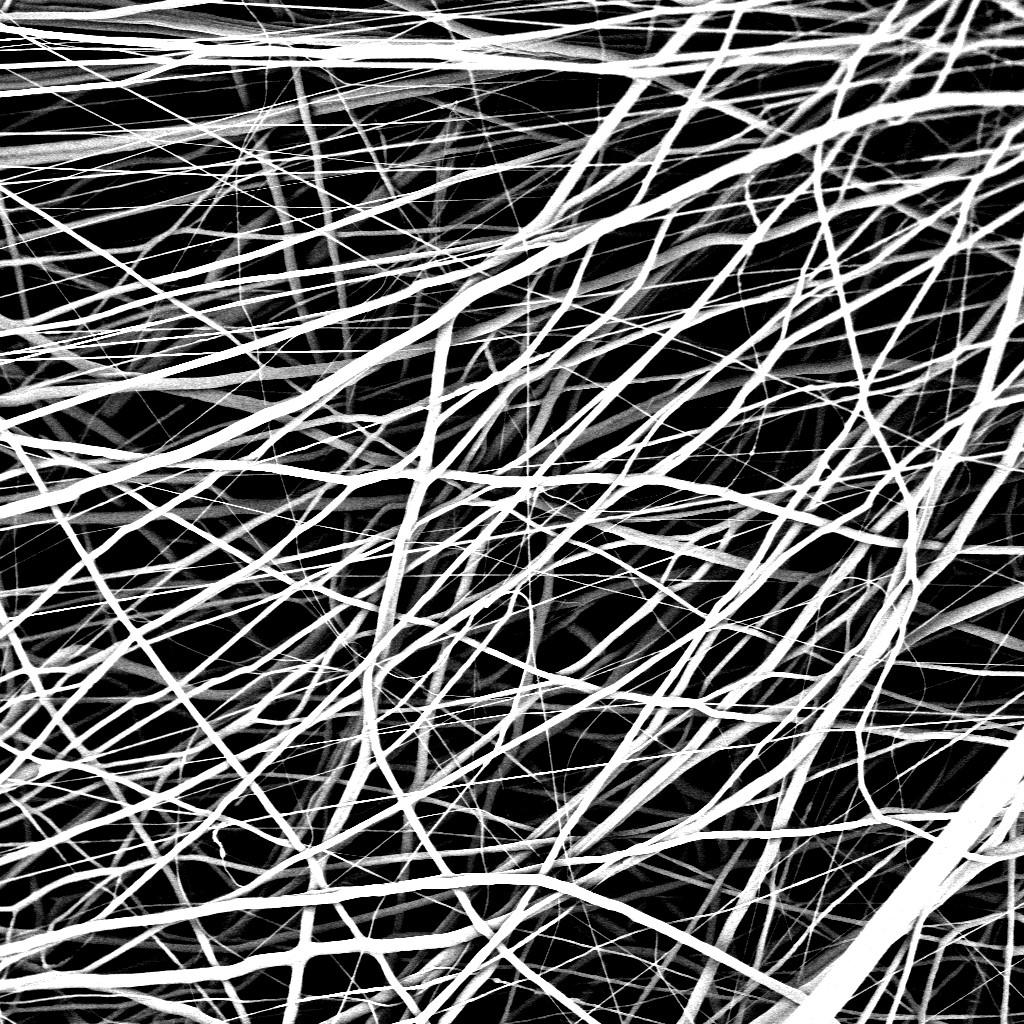

Supplement: S1 Data — (GZ) [file pone.0282903.s002.gz › data/PoreDiameter/RPM200 (2).jpg]

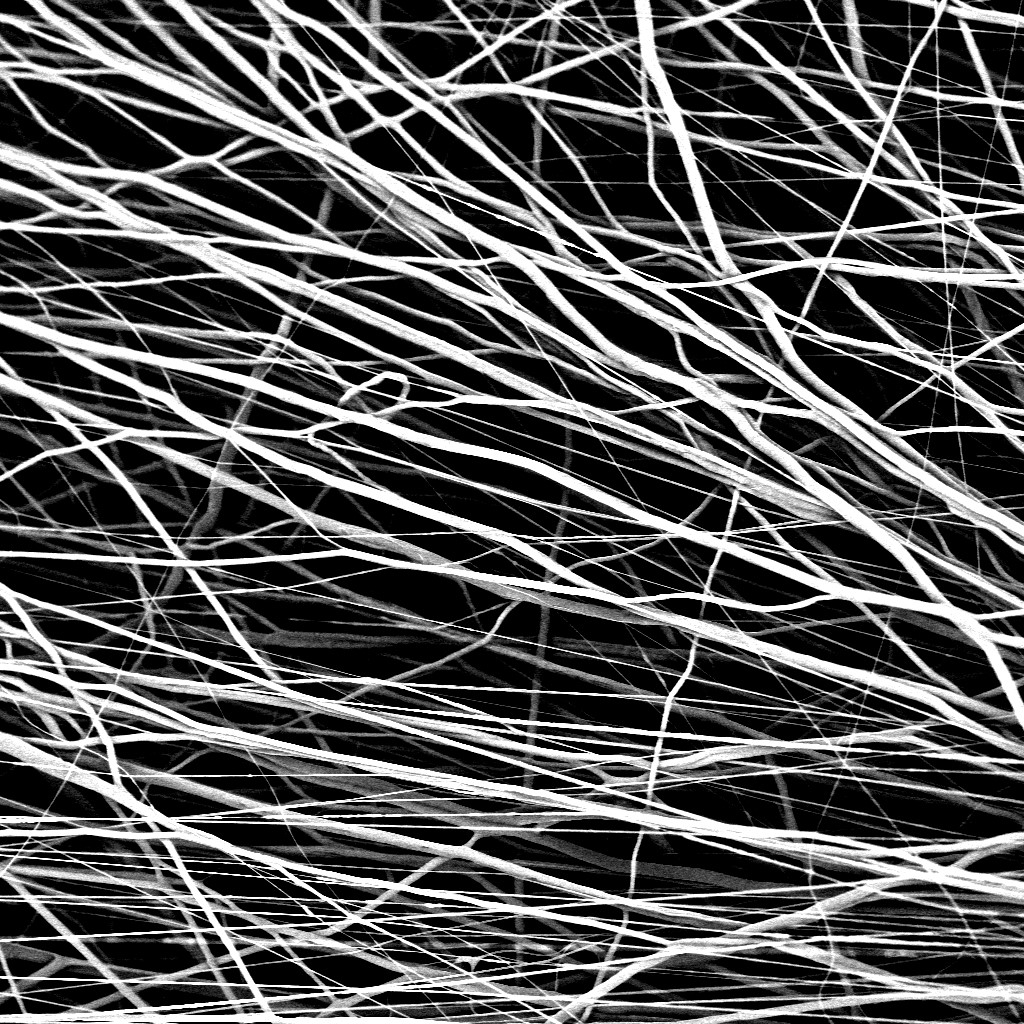

Supplement: S1 Data — (GZ) [file pone.0282903.s002.gz › data/PoreDiameter/RPM200 (3).jpg]

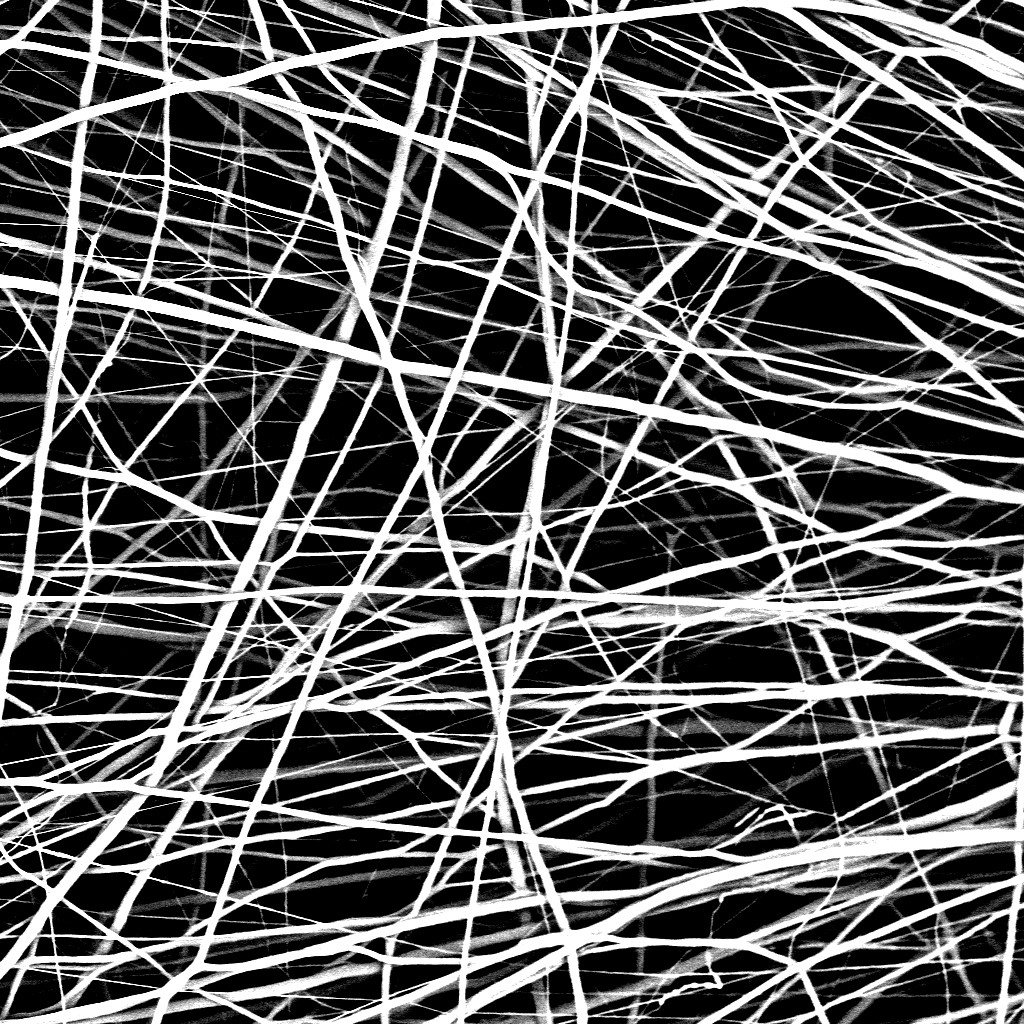

Supplement: S1 Data — (GZ) [file pone.0282903.s002.gz › data/PoreDiameter/RPM200 (4).jpg]

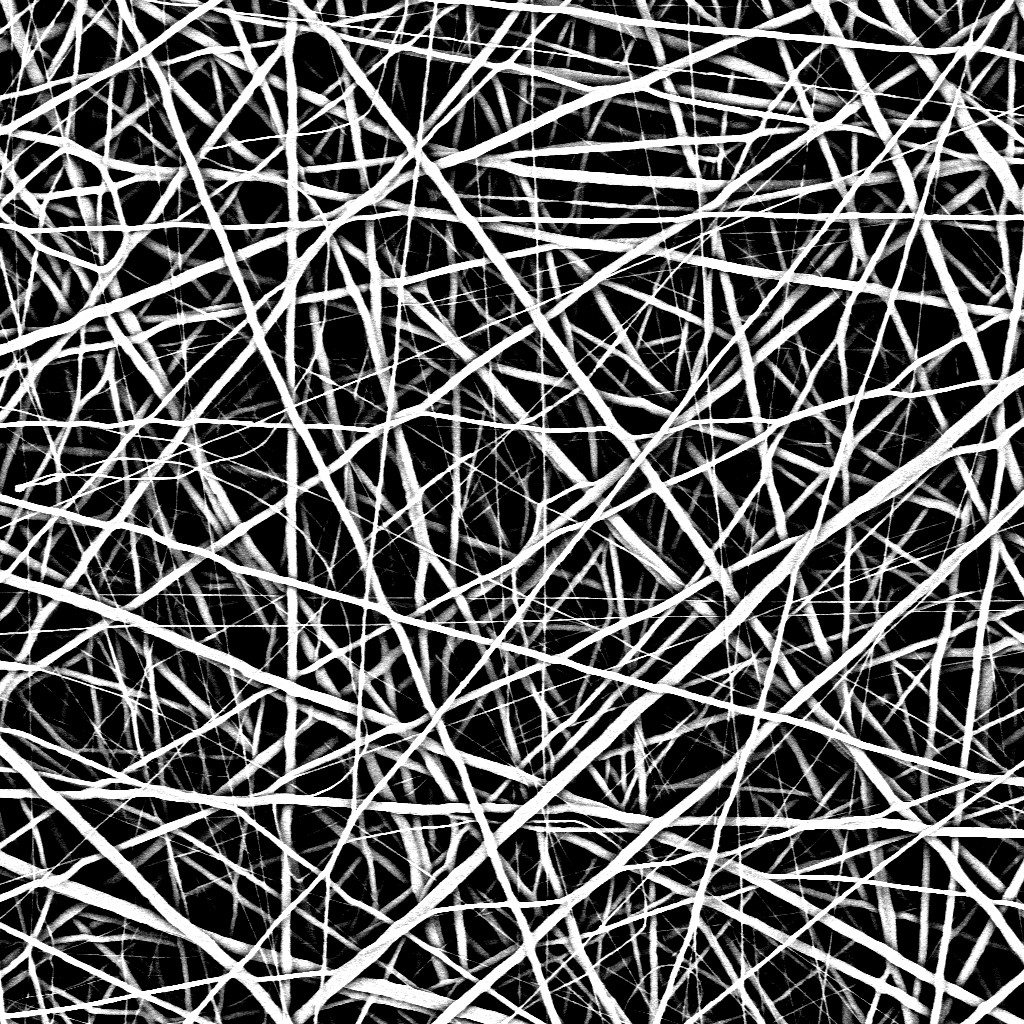

Supplement: S1 Data — (GZ) [file pone.0282903.s002.gz › data/PoreDiameter/RPM2000 (1).jpg]

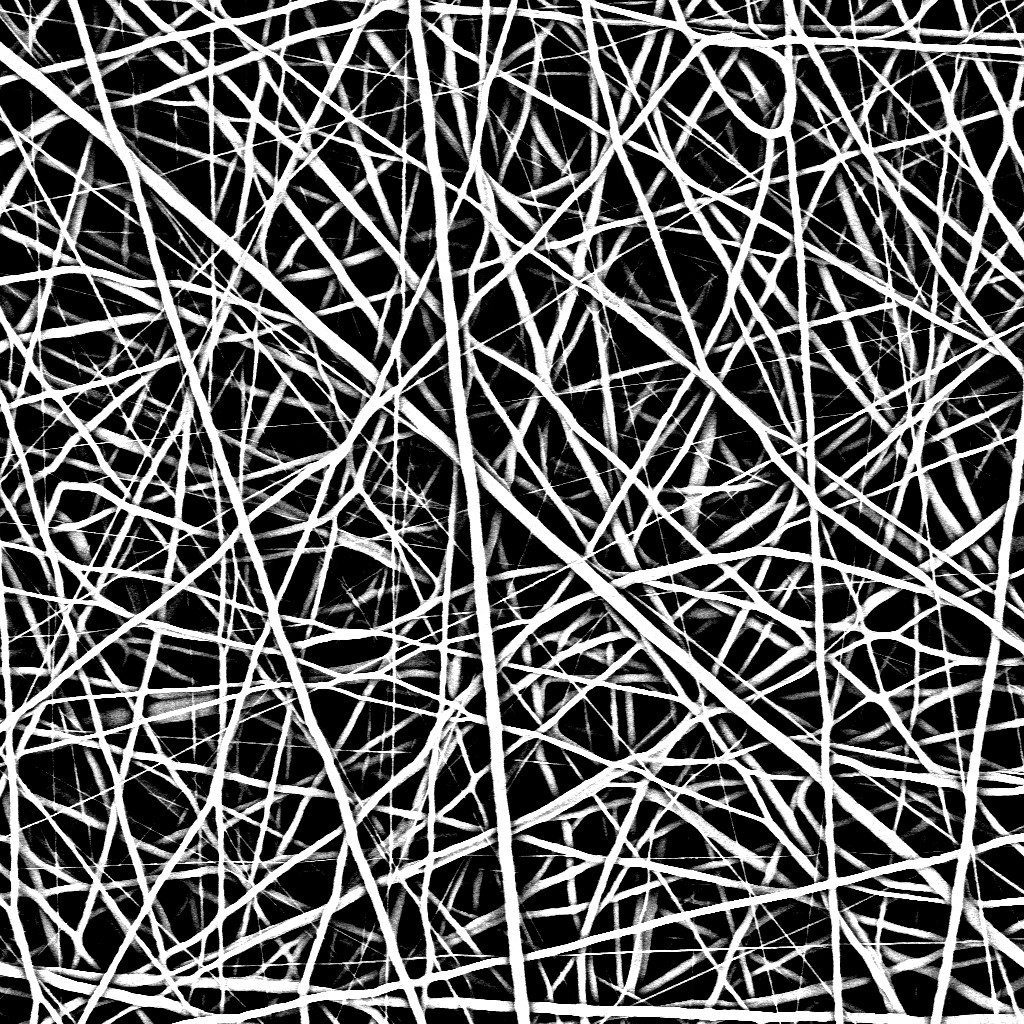

Supplement: S1 Data — (GZ) [file pone.0282903.s002.gz › data/PoreDiameter/RPM2000 (2).jpg]

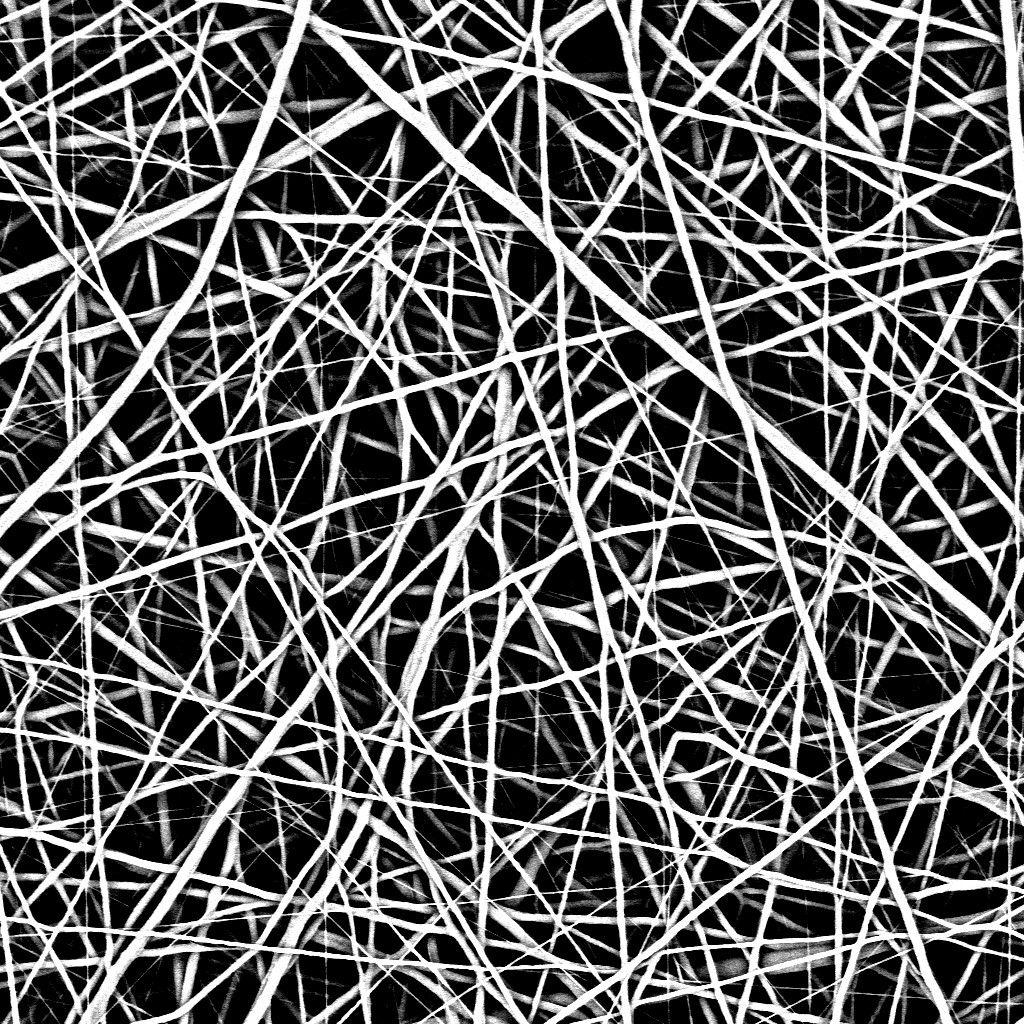

Supplement: S1 Data — (GZ) [file pone.0282903.s002.gz › data/PoreDiameter/RPM2000 (3).jpg]

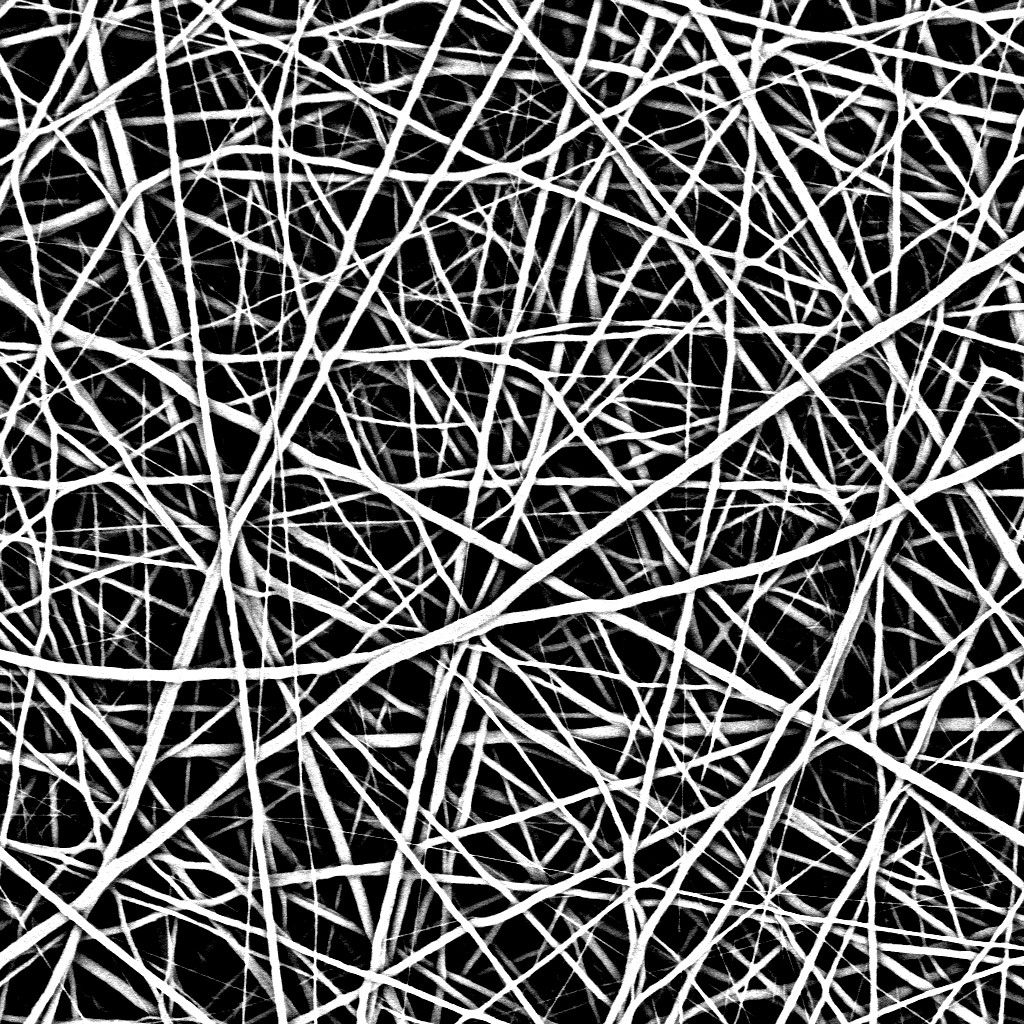

Supplement: S1 Data — (GZ) [file pone.0282903.s002.gz › data/PoreDiameter/RPM2000 (4).jpg]

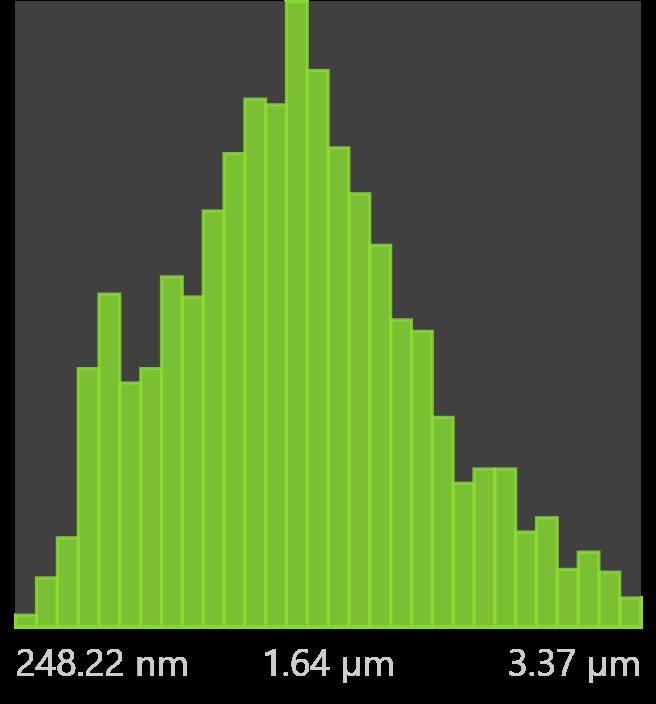

Supplement: S1 Data — (GZ) [file pone.0282903.s002.gz › data/FiberDiameter/Fibermetric for Central area/200rpm at 5,000X_20220113163858/Export/FiberHistogram.jpg]

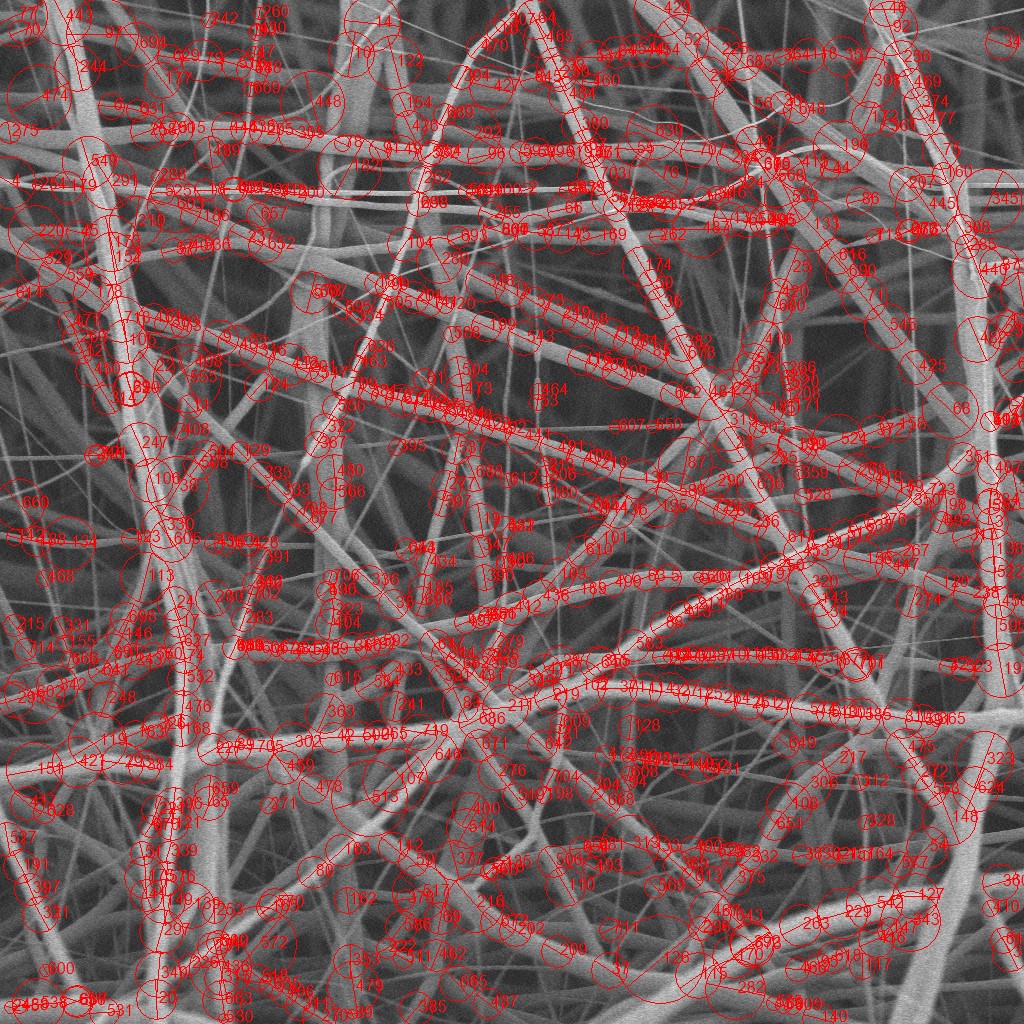

Supplement: S1 Data — (GZ) [file pone.0282903.s002.gz › data/FiberDiameter/Fibermetric for Central area/200rpm at 5,000X_20220113163858/Export/FibermetricImageMeasurements_3fe1d71a-1580-427d-9d1e-c36942401663.jpg]

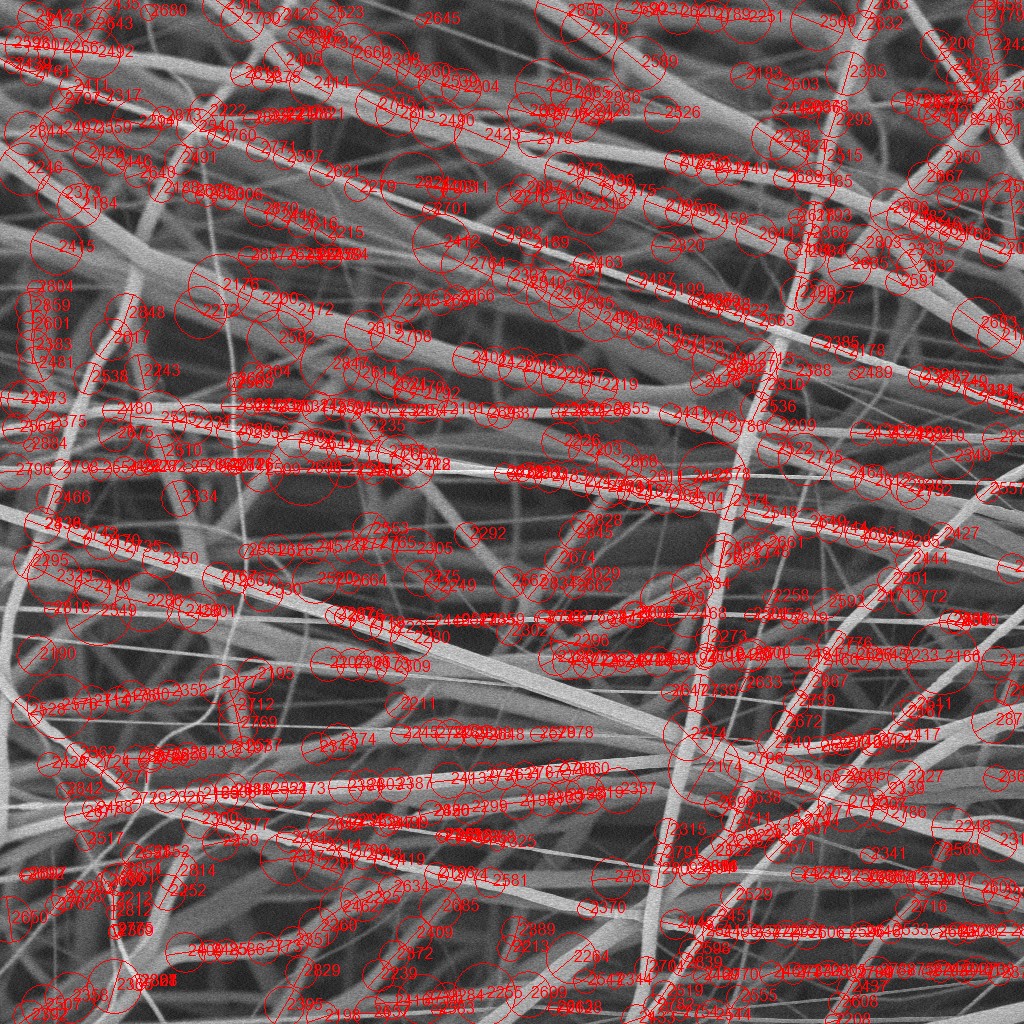

Supplement: S1 Data — (GZ) [file pone.0282903.s002.gz › data/FiberDiameter/Fibermetric for Central area/200rpm at 5,000X_20220113163858/Export/FibermetricImageMeasurements_71fc4b99-ff78-4664-8e0e-4719755ae8d2.jpg]

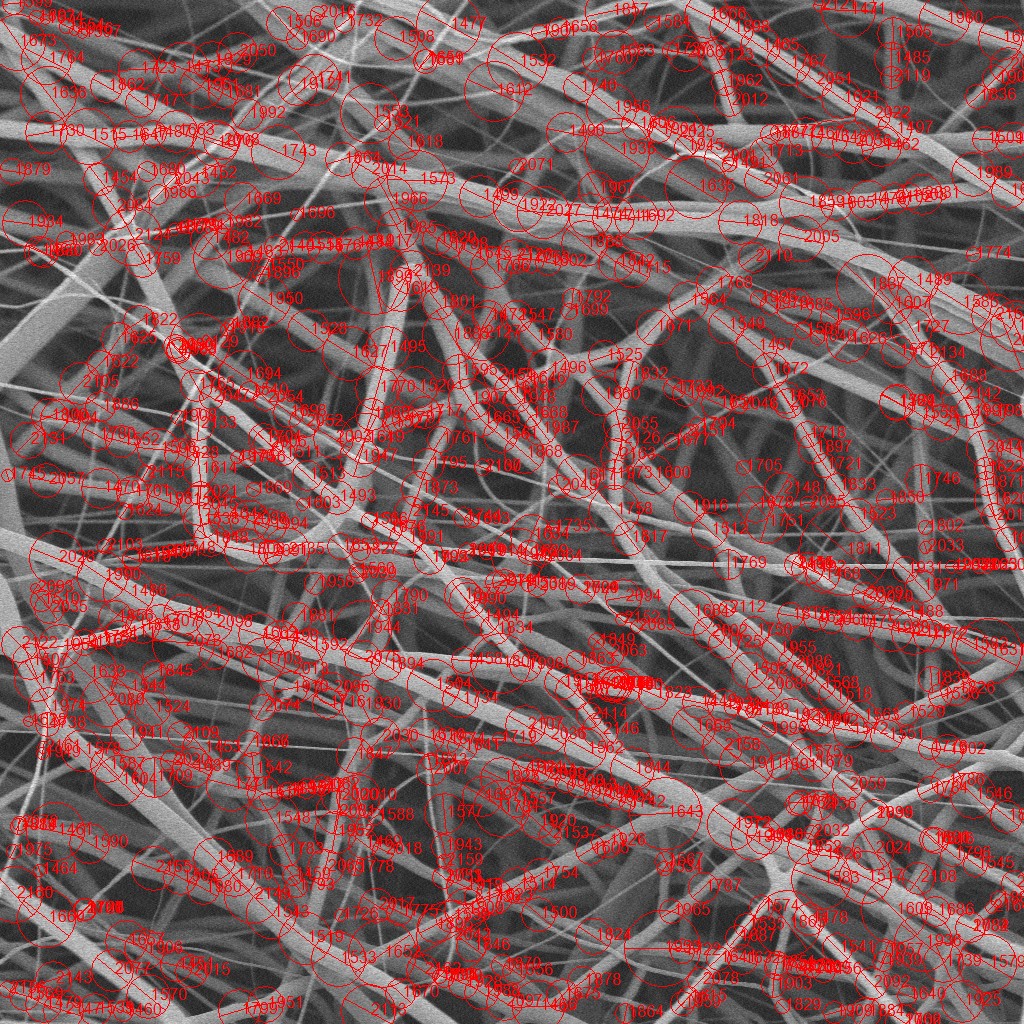

Supplement: S1 Data — (GZ) [file pone.0282903.s002.gz › data/FiberDiameter/Fibermetric for Central area/200rpm at 5,000X_20220113163858/Export/FibermetricImageMeasurements_be534f9c-dca1-49a1-81a4-109e6ea12362.jpg]

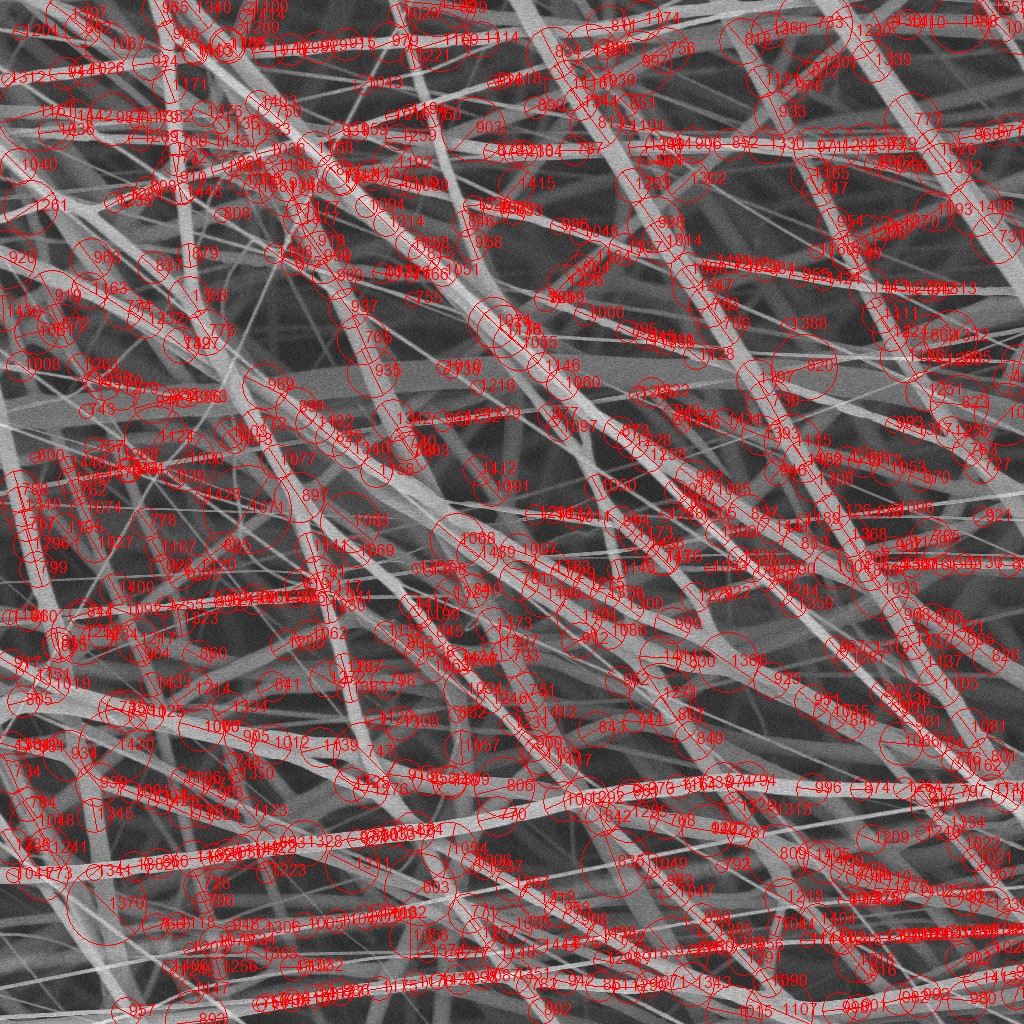

Supplement: S1 Data — (GZ) [file pone.0282903.s002.gz › data/FiberDiameter/Fibermetric for Central area/200rpm at 5,000X_20220113163858/Export/FibermetricImageMeasurements_f95283c8-728f-497f-9feb-b4bfb6c78d94.jpg]

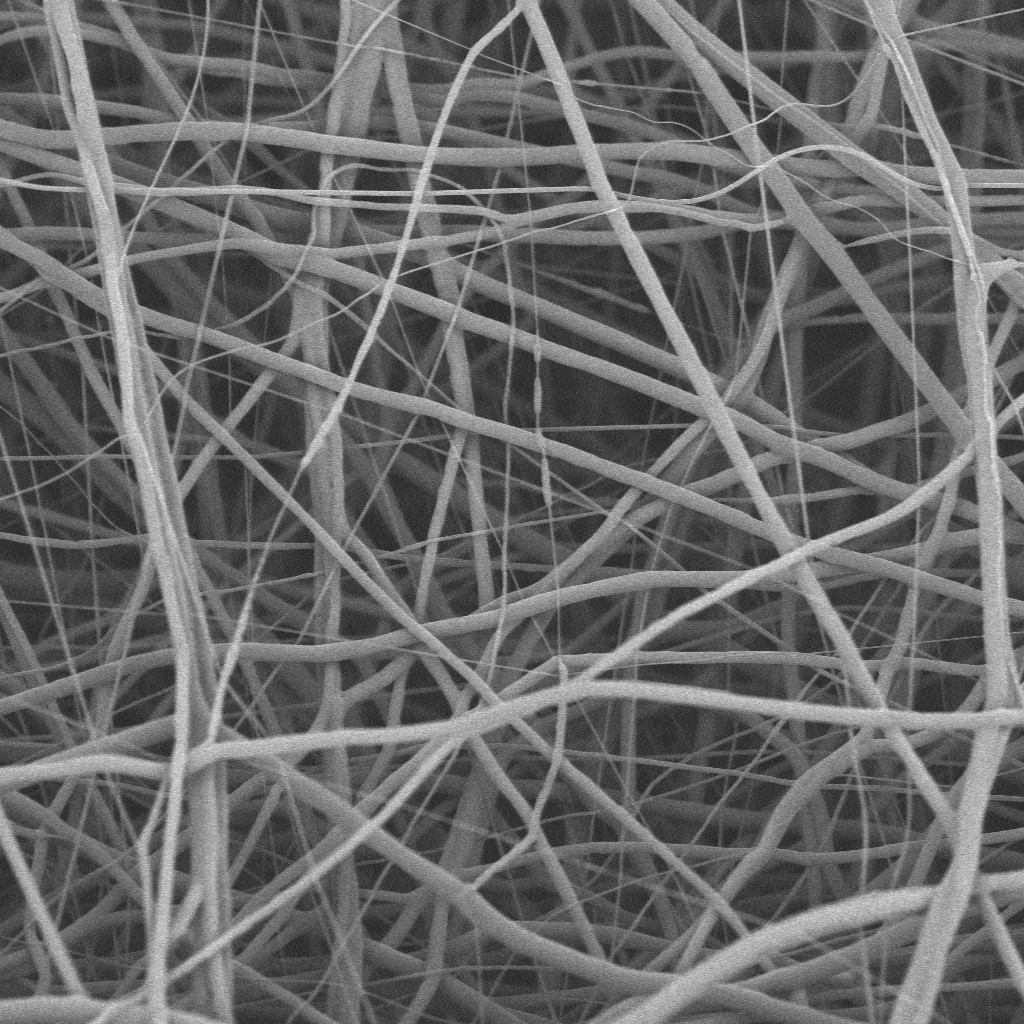

Supplement: S1 Data — (GZ) [file pone.0282903.s002.gz › data/FiberDiameter/Fibermetric for Central area/200rpm at 5,000X_20220113163858/Export/Fibermetric_Image0001.jpg]

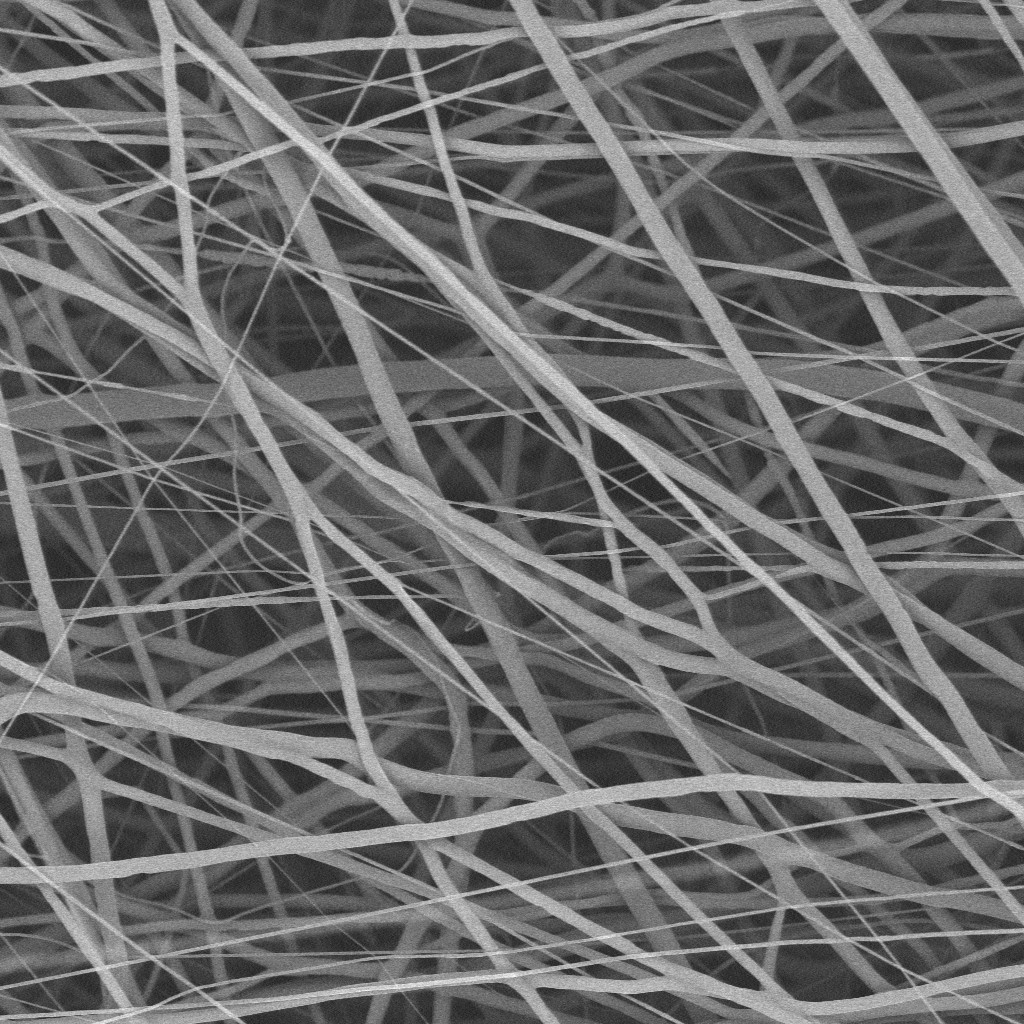

Supplement: S1 Data — (GZ) [file pone.0282903.s002.gz › data/FiberDiameter/Fibermetric for Central area/200rpm at 5,000X_20220113163858/Export/Fibermetric_Image0002.jpg]

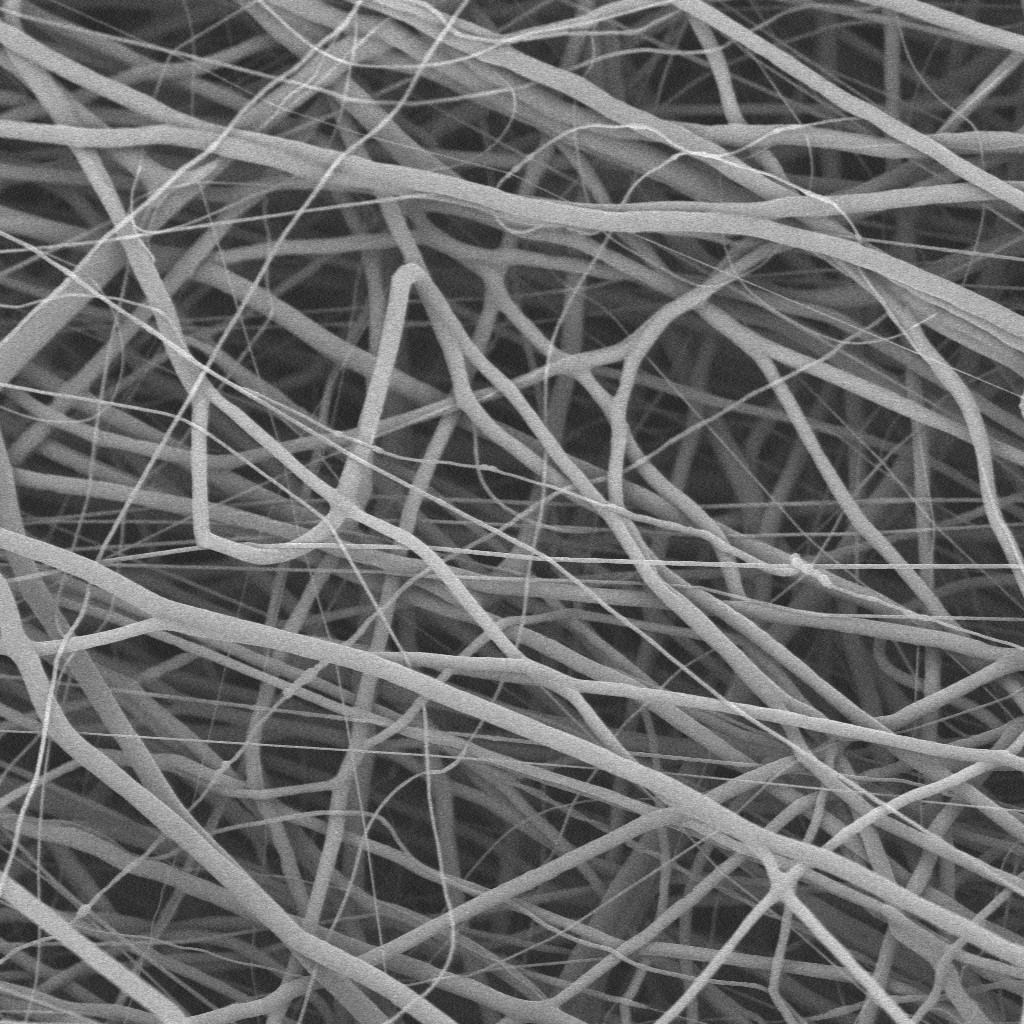

Supplement: S1 Data — (GZ) [file pone.0282903.s002.gz › data/FiberDiameter/Fibermetric for Central area/200rpm at 5,000X_20220113163858/Export/Fibermetric_Image0003.jpg]

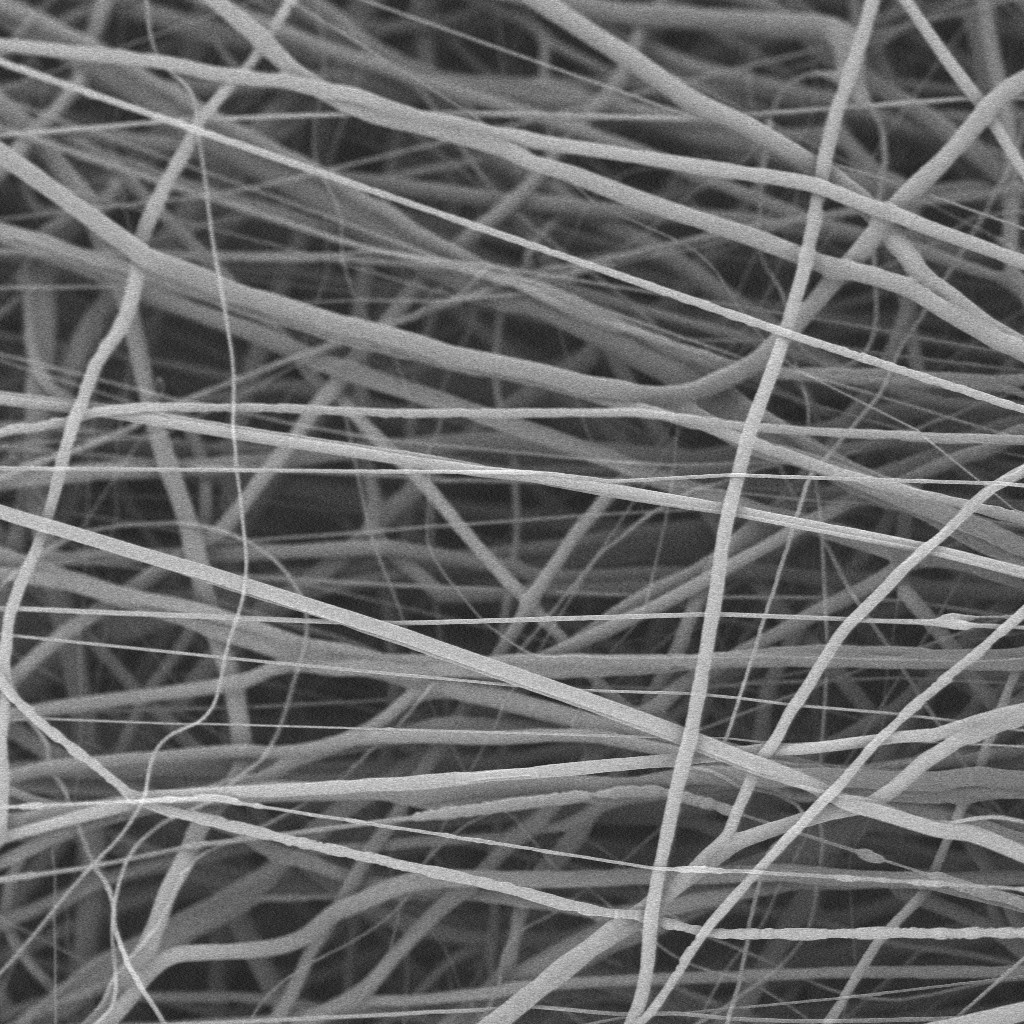

Supplement: S1 Data — (GZ) [file pone.0282903.s002.gz › data/FiberDiameter/Fibermetric for Central area/200rpm at 5,000X_20220113163858/Export/Fibermetric_Image0004.jpg]

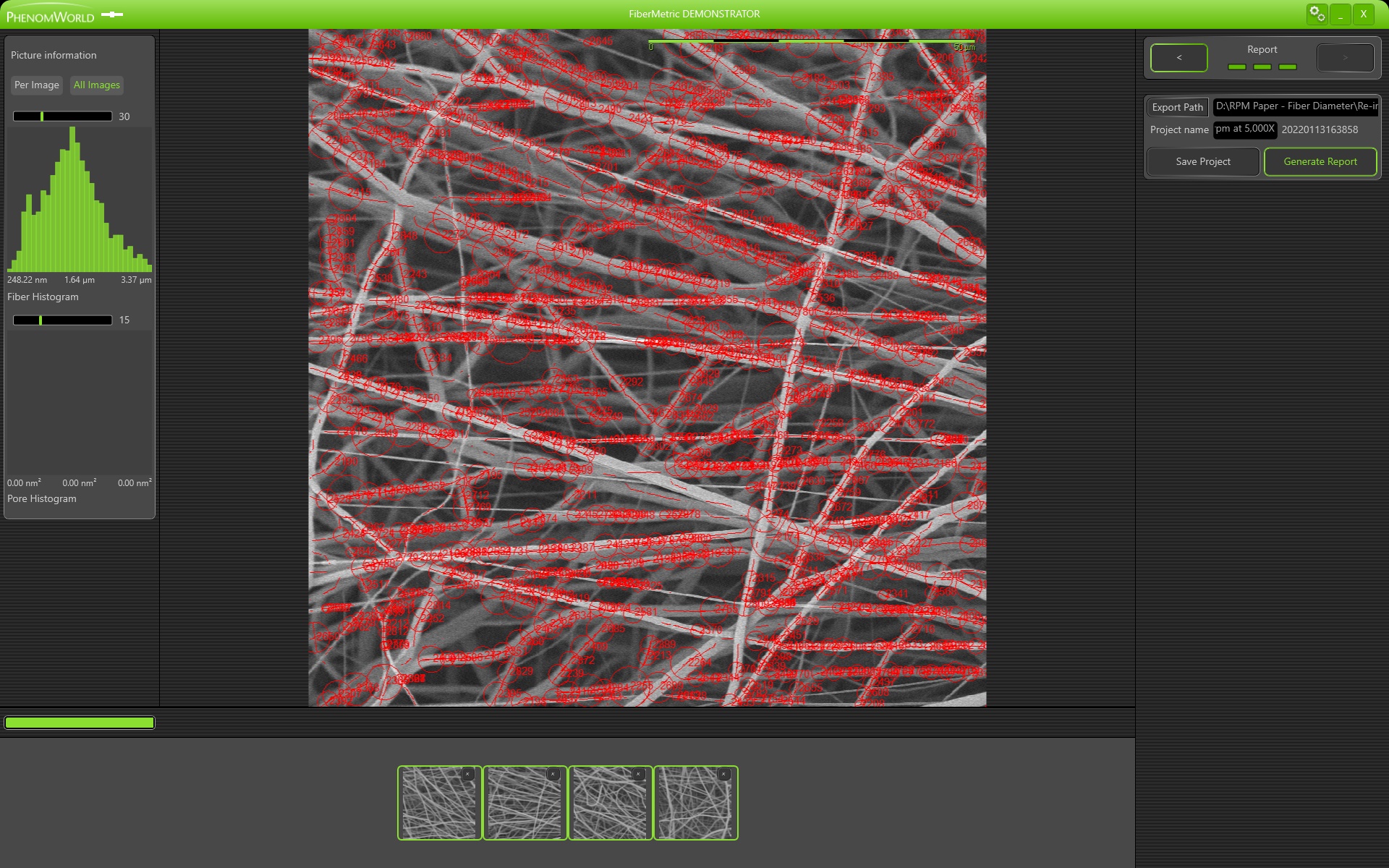

Supplement: S1 Data — (GZ) [file pone.0282903.s002.gz › data/FiberDiameter/Fibermetric for Central area/200rpm at 5,000X_20220113163858/Export/Screenshot.jpg]

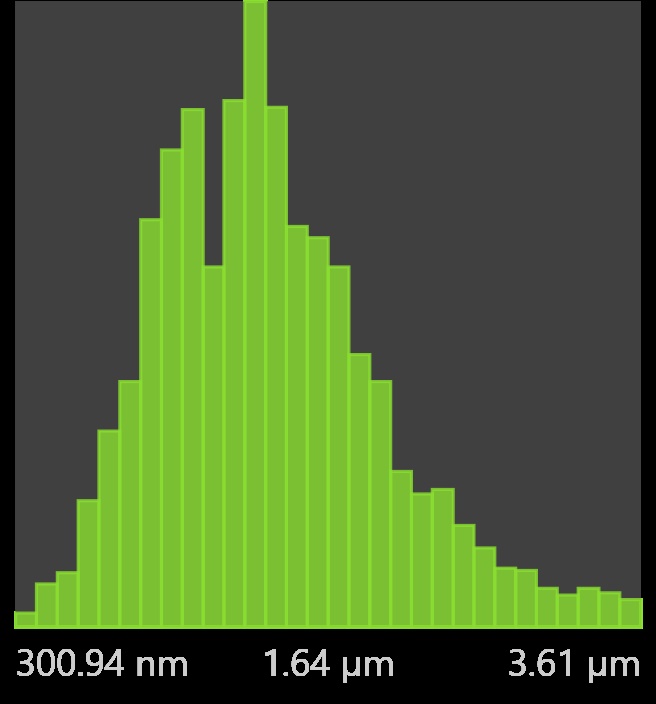

Supplement: S1 Data — (GZ) [file pone.0282903.s002.gz › data/FiberDiameter/Fibermetric for Central area/2,000rpm at 5,000X_20220113171250/Export/FiberHistogram.jpg]

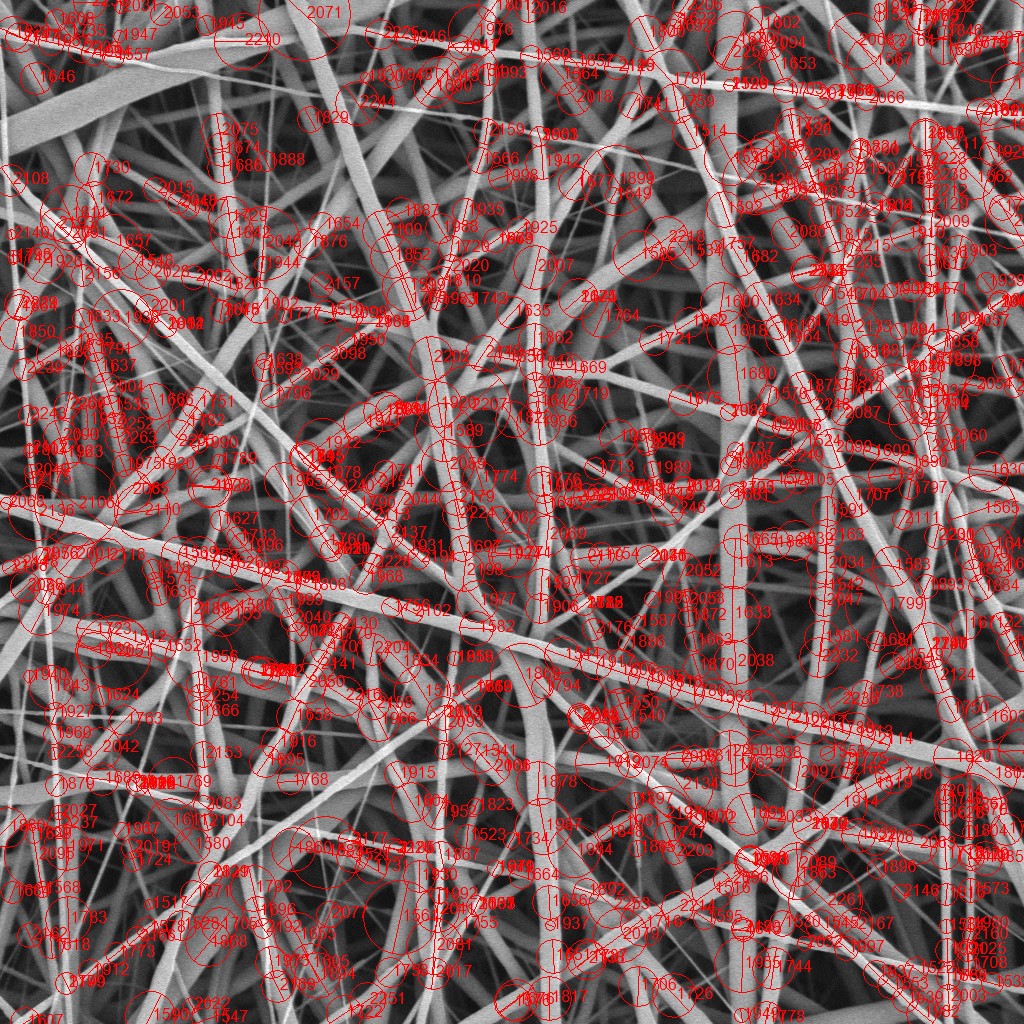

Supplement: S1 Data — (GZ) [file pone.0282903.s002.gz › data/FiberDiameter/Fibermetric for Central area/2,000rpm at 5,000X_20220113171250/Export/FibermetricImageMeasurements_070b5c6a-874f-4d1b-aaa3-2e43d89e3b47.jpg]

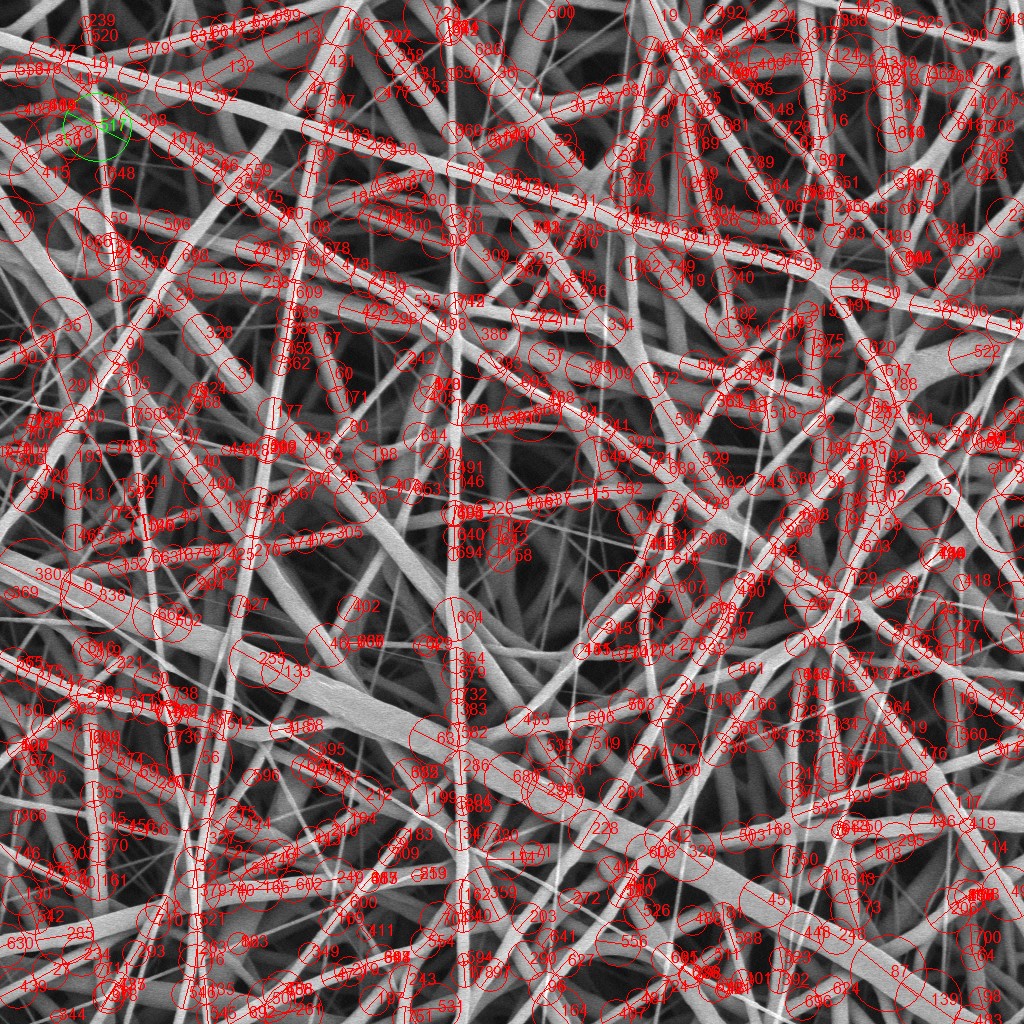

Supplement: S1 Data — (GZ) [file pone.0282903.s002.gz › data/FiberDiameter/Fibermetric for Central area/2,000rpm at 5,000X_20220113171250/Export/FibermetricImageMeasurements_234ebf67-ff7b-4fd9-a19c-cd184a88f16b.jpg]

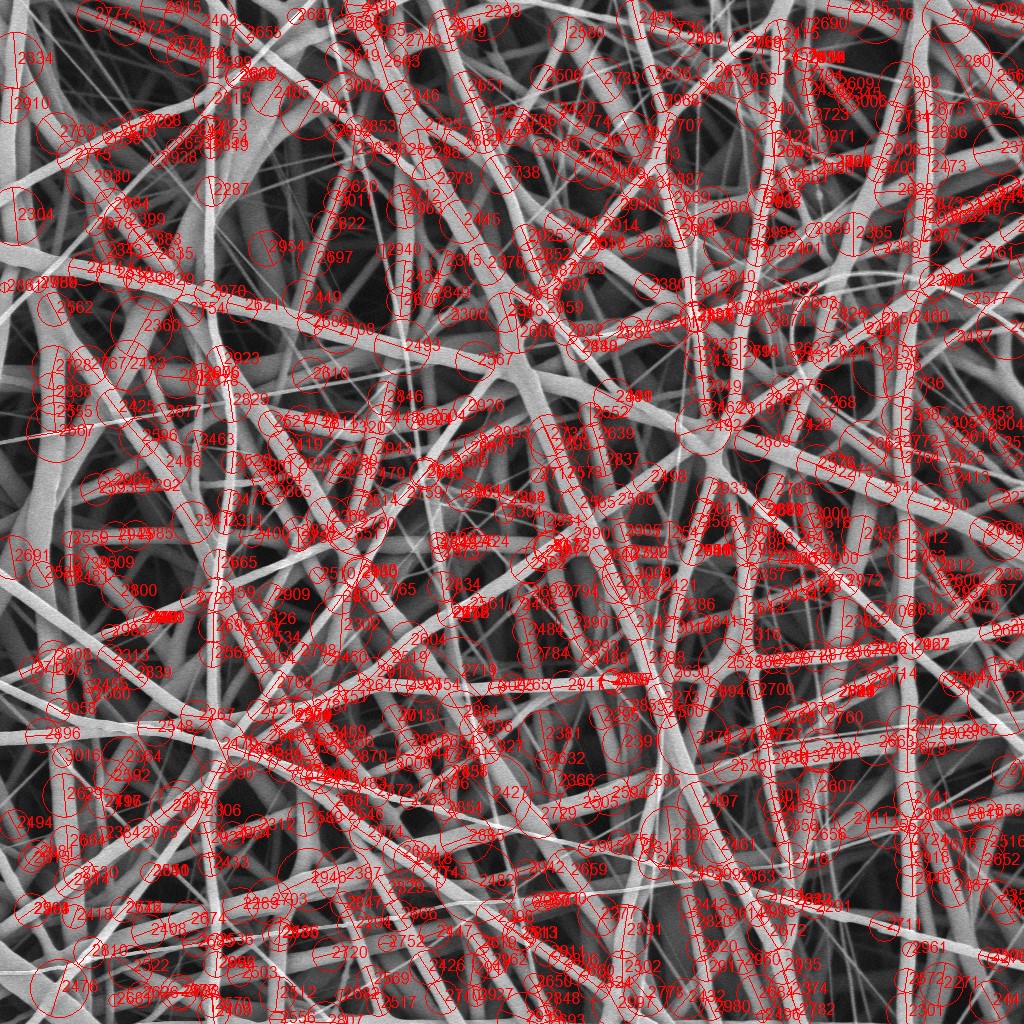

Supplement: S1 Data — (GZ) [file pone.0282903.s002.gz › data/FiberDiameter/Fibermetric for Central area/2,000rpm at 5,000X_20220113171250/Export/FibermetricImageMeasurements_9eda6ef4-6d19-4202-b6b8-76af7e8a3ea8.jpg]

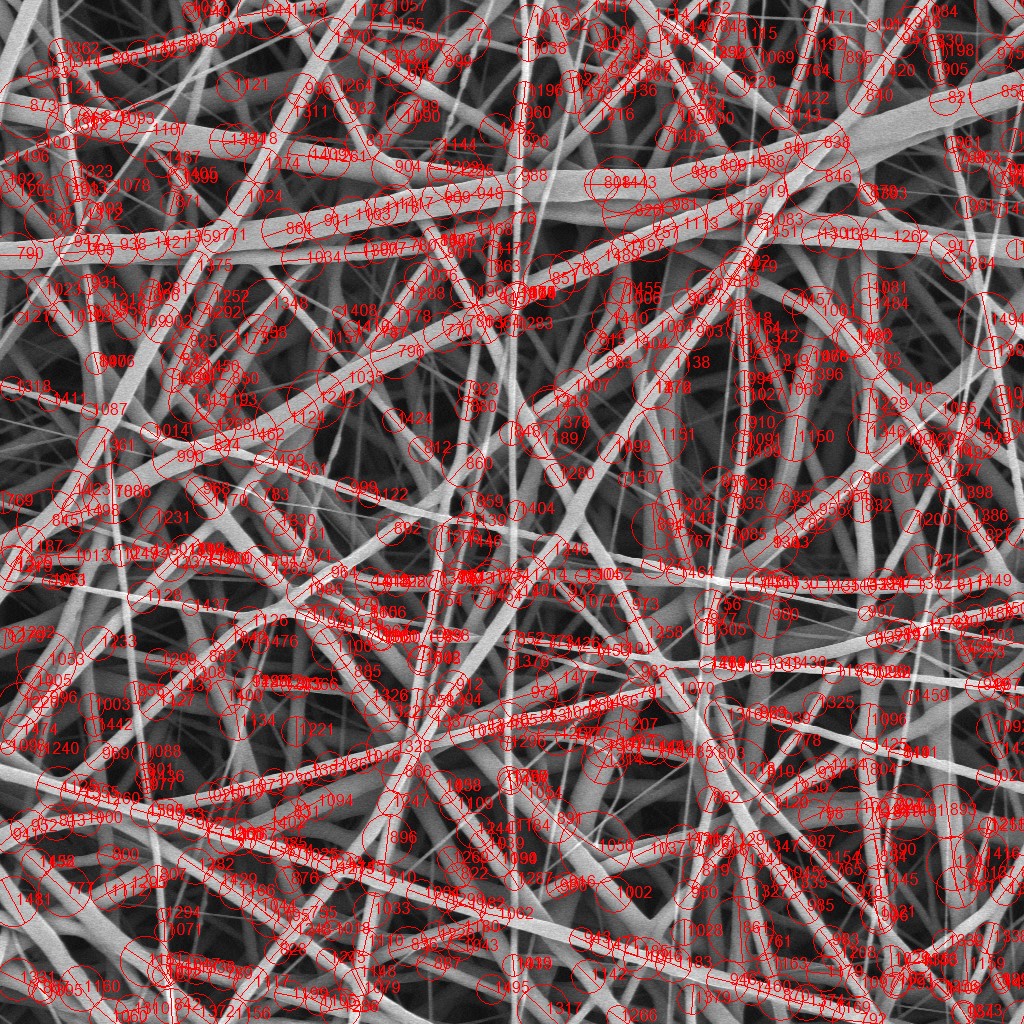

Supplement: S1 Data — (GZ) [file pone.0282903.s002.gz › data/FiberDiameter/Fibermetric for Central area/2,000rpm at 5,000X_20220113171250/Export/FibermetricImageMeasurements_d6c4c98e-0d97-41d4-96cb-5e04f11e80c9.jpg]

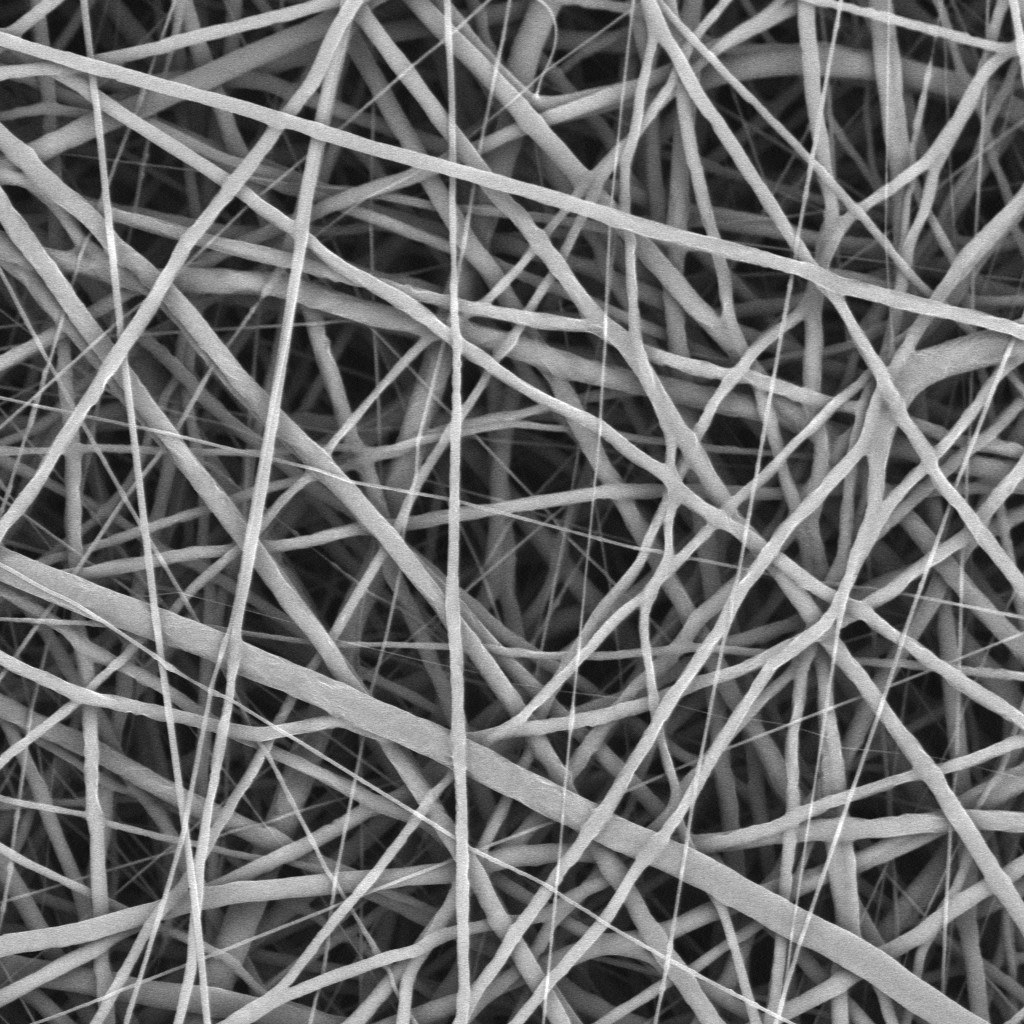

Supplement: S1 Data — (GZ) [file pone.0282903.s002.gz › data/FiberDiameter/Fibermetric for Central area/2,000rpm at 5,000X_20220113171250/Export/Fibermetric_Image0001.jpg]

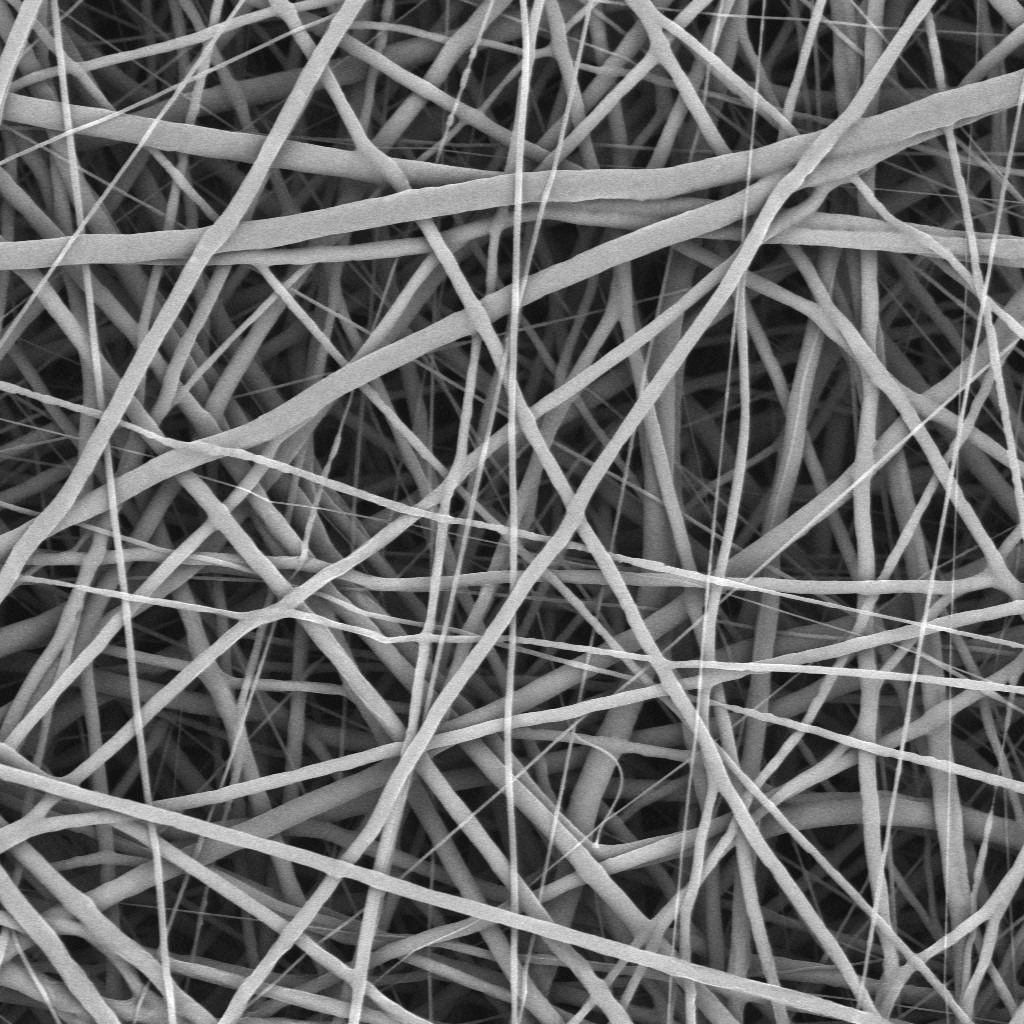

Supplement: S1 Data — (GZ) [file pone.0282903.s002.gz › data/FiberDiameter/Fibermetric for Central area/2,000rpm at 5,000X_20220113171250/Export/Fibermetric_Image0002.jpg]

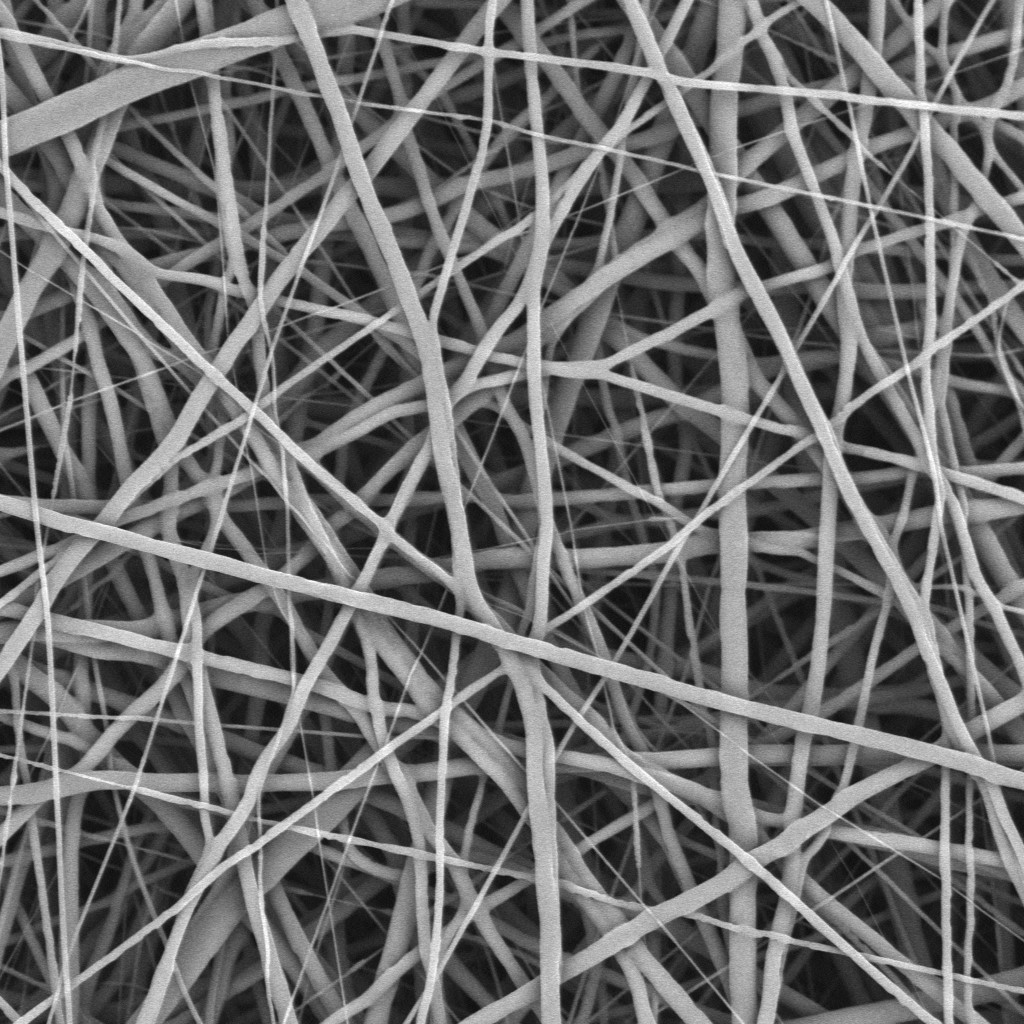

Supplement: S1 Data — (GZ) [file pone.0282903.s002.gz › data/FiberDiameter/Fibermetric for Central area/2,000rpm at 5,000X_20220113171250/Export/Fibermetric_Image0003.jpg]

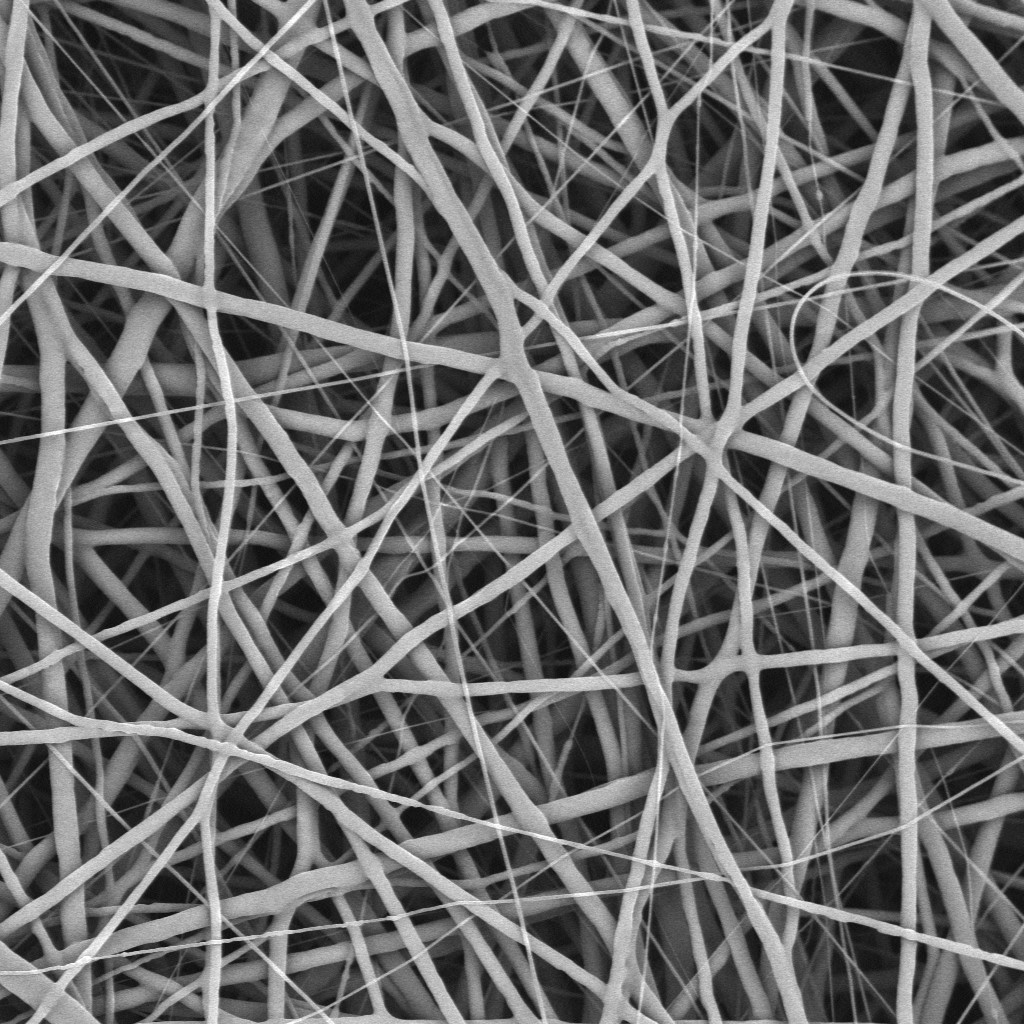

Supplement: S1 Data — (GZ) [file pone.0282903.s002.gz › data/FiberDiameter/Fibermetric for Central area/2,000rpm at 5,000X_20220113171250/Export/Fibermetric_Image0004.jpg]

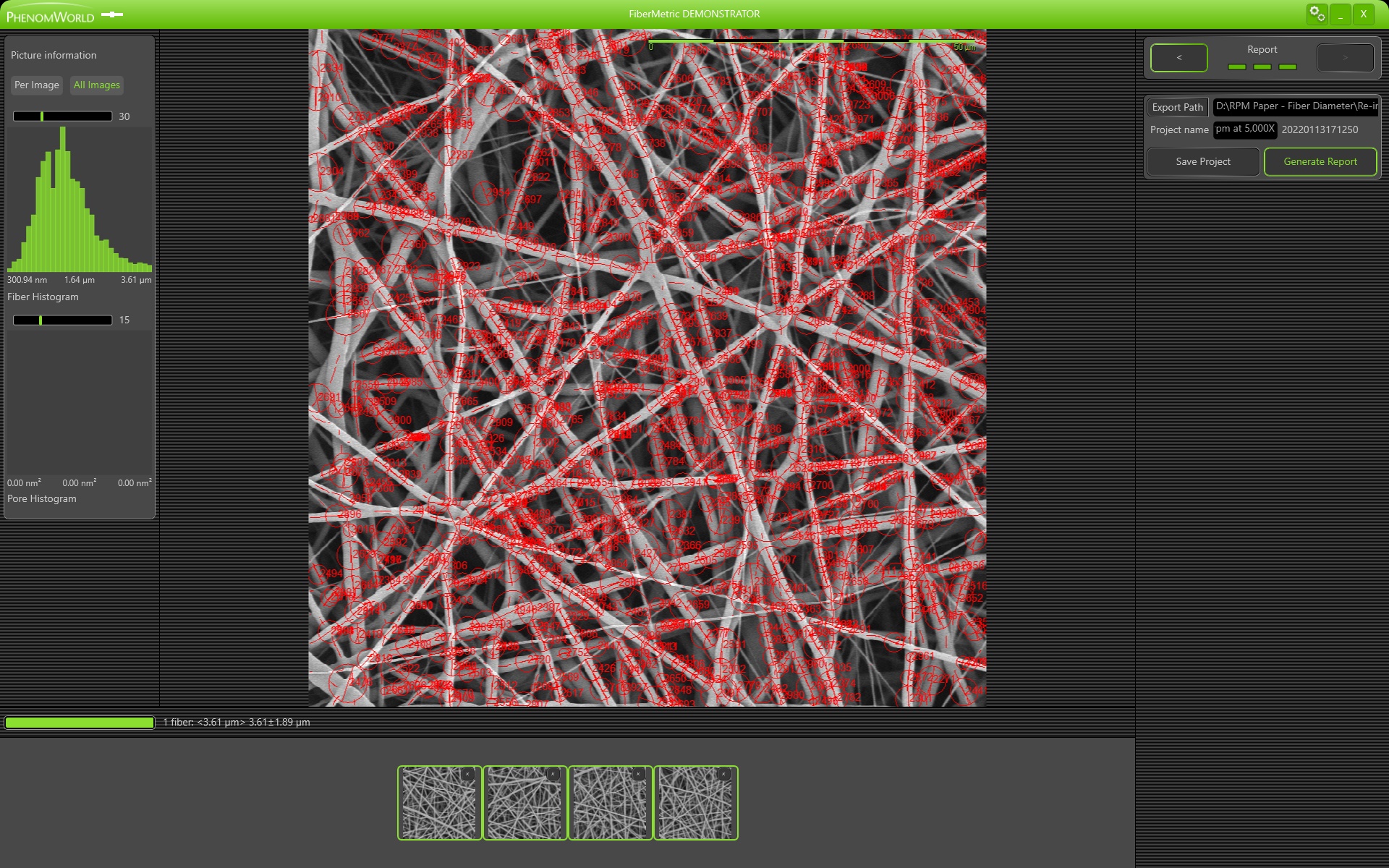

Supplement: S1 Data — (GZ) [file pone.0282903.s002.gz › data/FiberDiameter/Fibermetric for Central area/2,000rpm at 5,000X_20220113171250/Export/Screenshot.jpg]

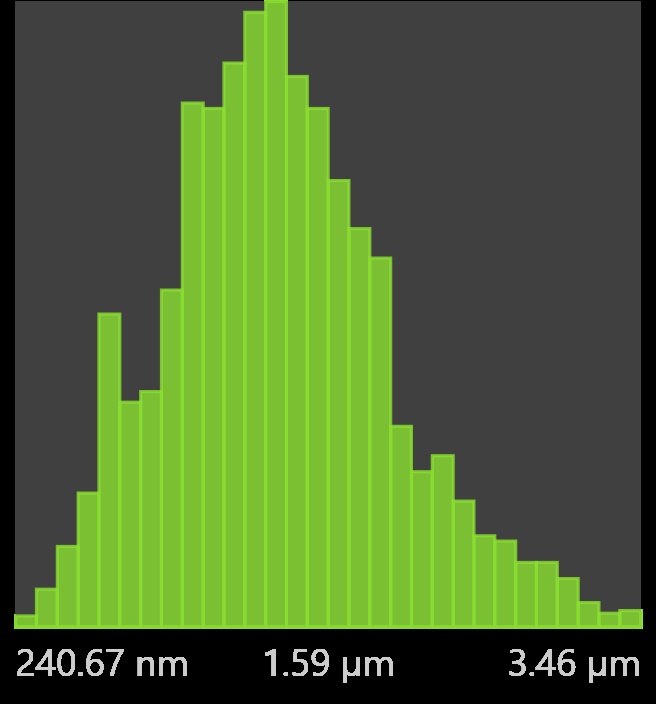

Supplement: S1 Data — (GZ) [file pone.0282903.s002.gz › data/FiberDiameter/Fibermetric for Central area/1,100rpm at 5,000X_20220113164859/Export/FiberHistogram.jpg]

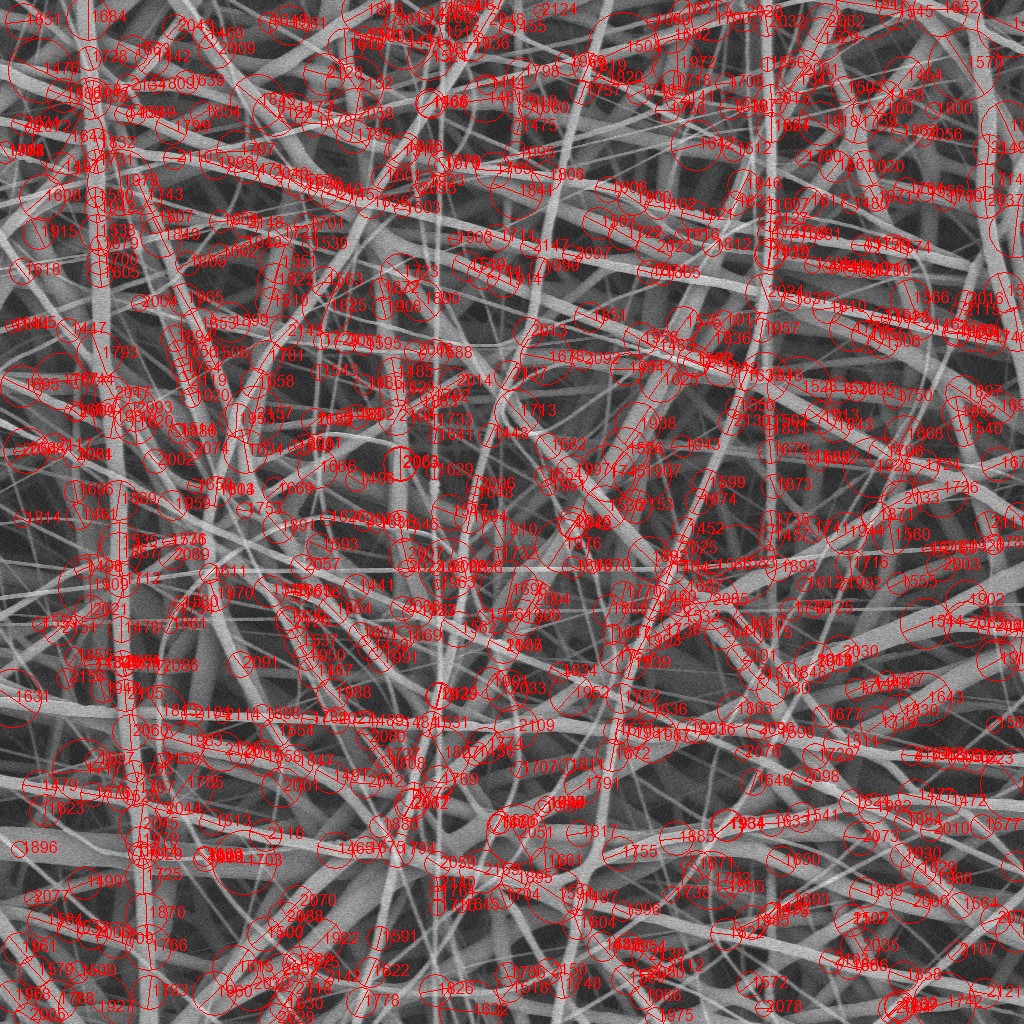

Supplement: S1 Data — (GZ) [file pone.0282903.s002.gz › data/FiberDiameter/Fibermetric for Central area/1,100rpm at 5,000X_20220113164859/Export/FibermetricImageMeasurements_03173b91-69d1-4c31-a468-7ff795785d42.jpg]

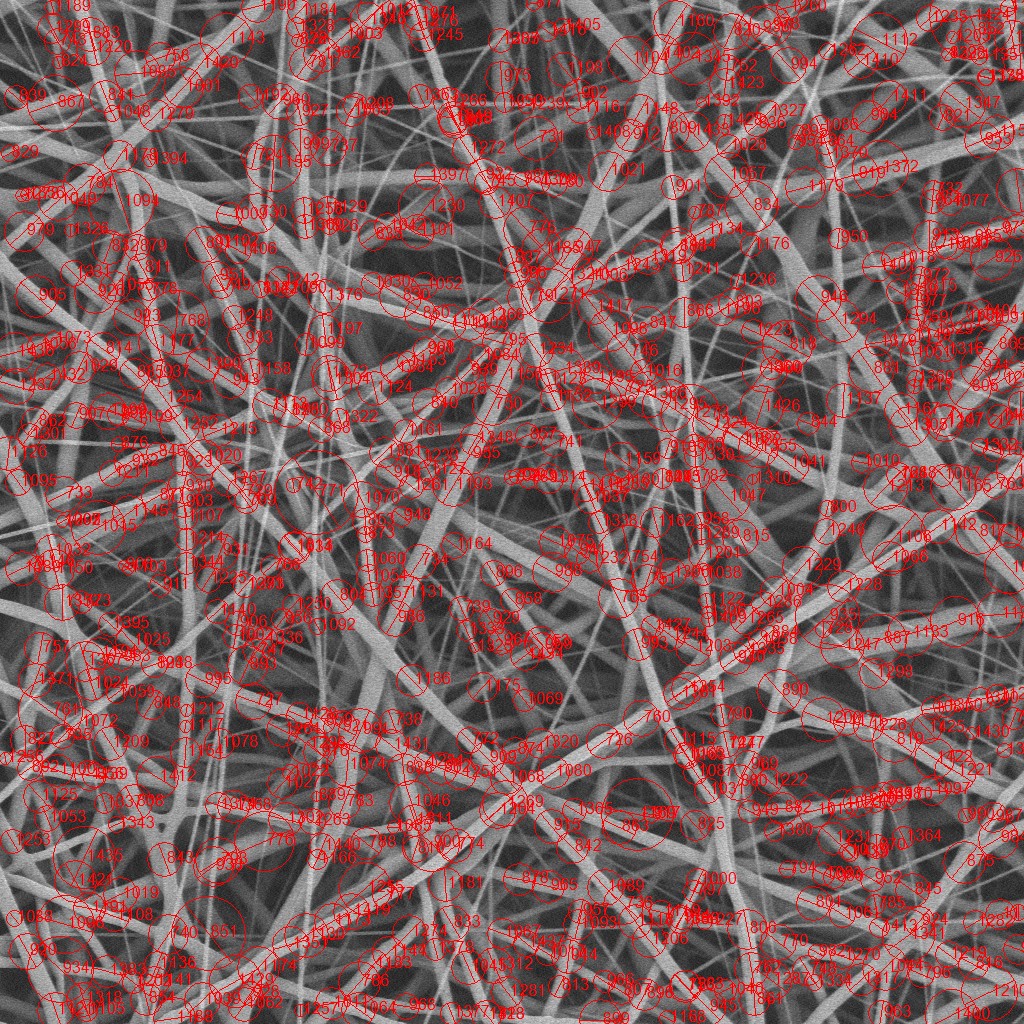

Supplement: S1 Data — (GZ) [file pone.0282903.s002.gz › data/FiberDiameter/Fibermetric for Central area/1,100rpm at 5,000X_20220113164859/Export/FibermetricImageMeasurements_0c55eda2-fc49-47a9-93cd-eb186e117346.jpg]

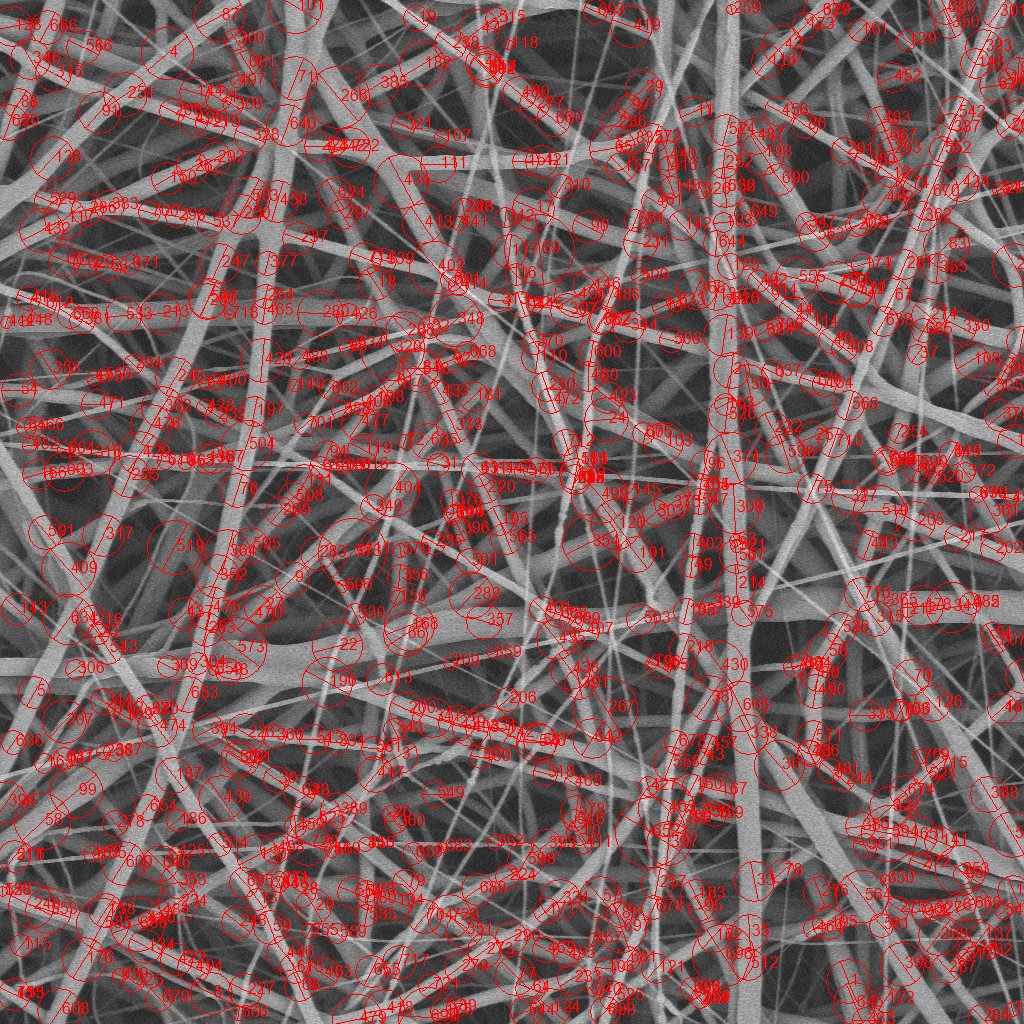

Supplement: S1 Data — (GZ) [file pone.0282903.s002.gz › data/FiberDiameter/Fibermetric for Central area/1,100rpm at 5,000X_20220113164859/Export/FibermetricImageMeasurements_247bc545-e0ae-4e8b-9ba5-07adcd716d14.jpg]

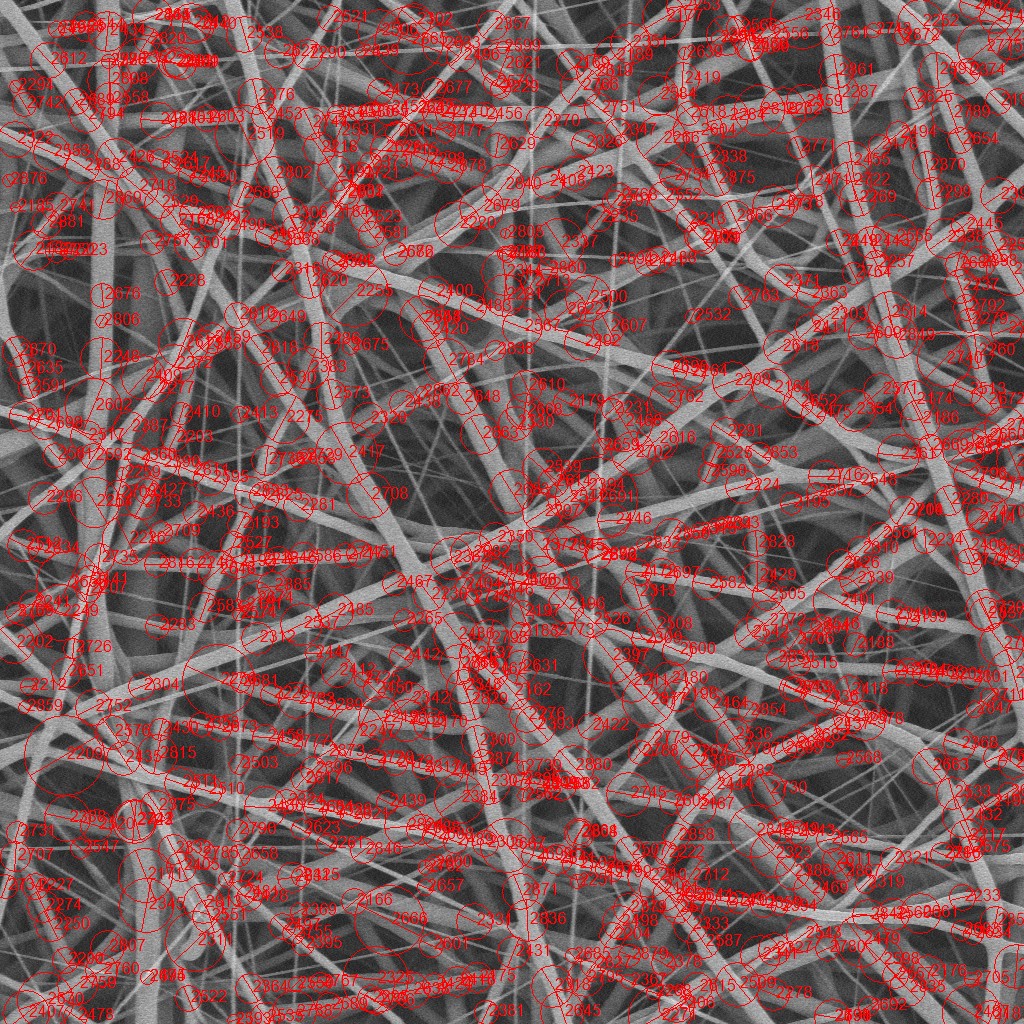

Supplement: S1 Data — (GZ) [file pone.0282903.s002.gz › data/FiberDiameter/Fibermetric for Central area/1,100rpm at 5,000X_20220113164859/Export/FibermetricImageMeasurements_9b15942d-a9a4-419f-ae16-c6e84c0bad79.jpg]

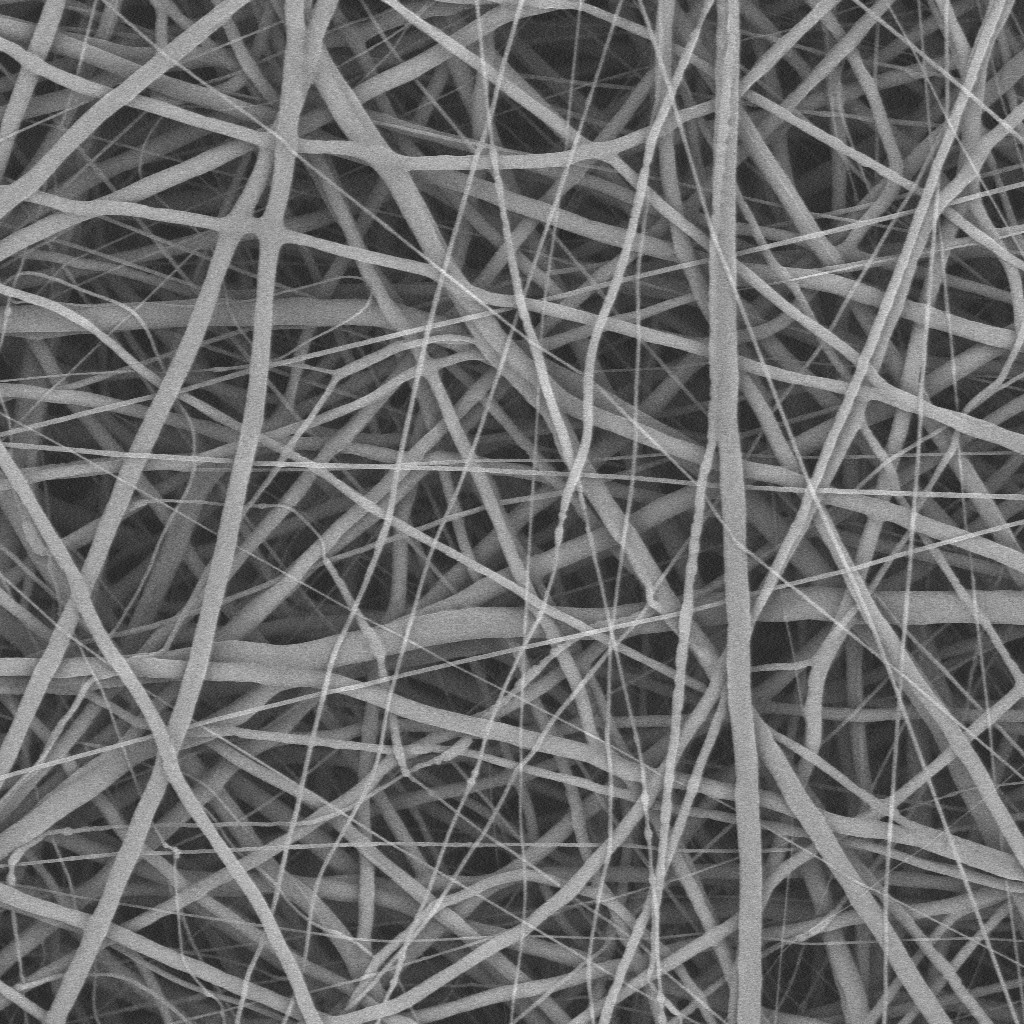

Supplement: S1 Data — (GZ) [file pone.0282903.s002.gz › data/FiberDiameter/Fibermetric for Central area/1,100rpm at 5,000X_20220113164859/Export/Fibermetric_Image0001.jpg]

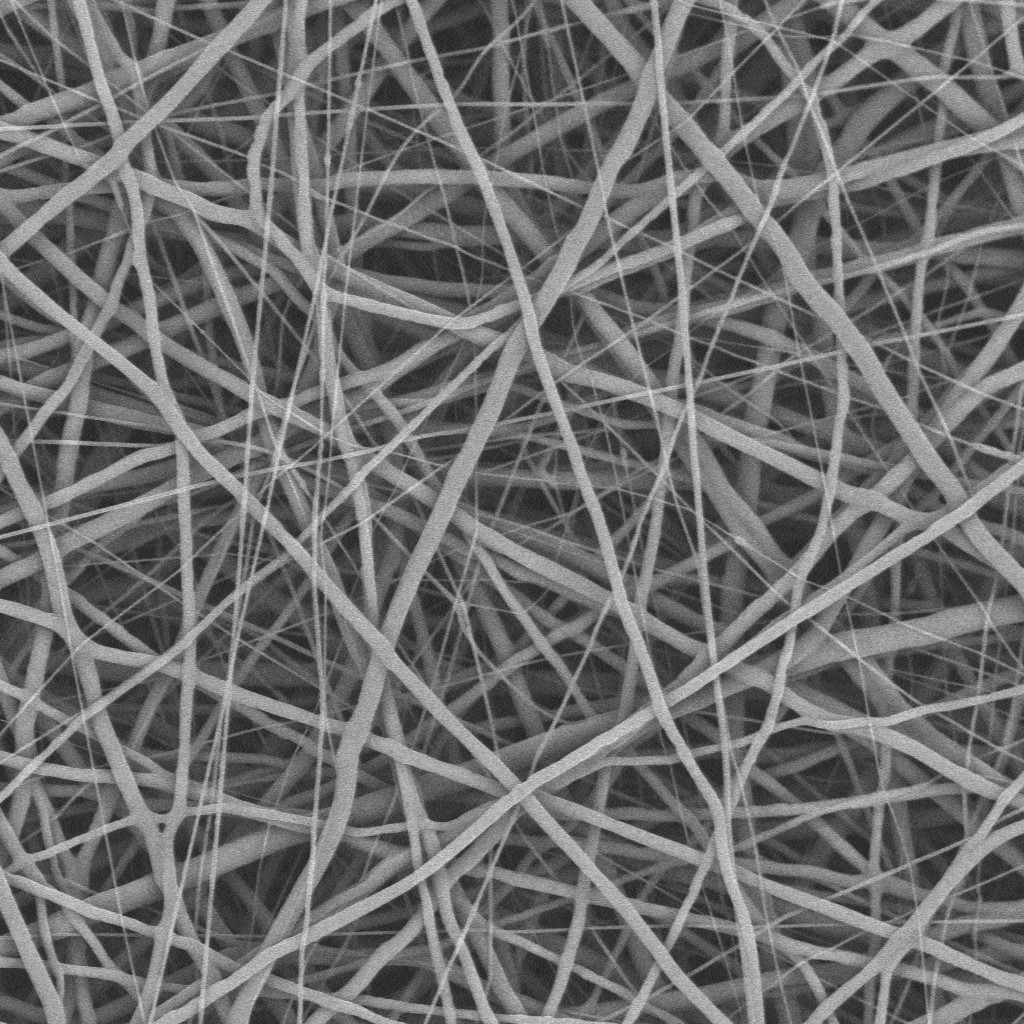

Supplement: S1 Data — (GZ) [file pone.0282903.s002.gz › data/FiberDiameter/Fibermetric for Central area/1,100rpm at 5,000X_20220113164859/Export/Fibermetric_Image0002.jpg]

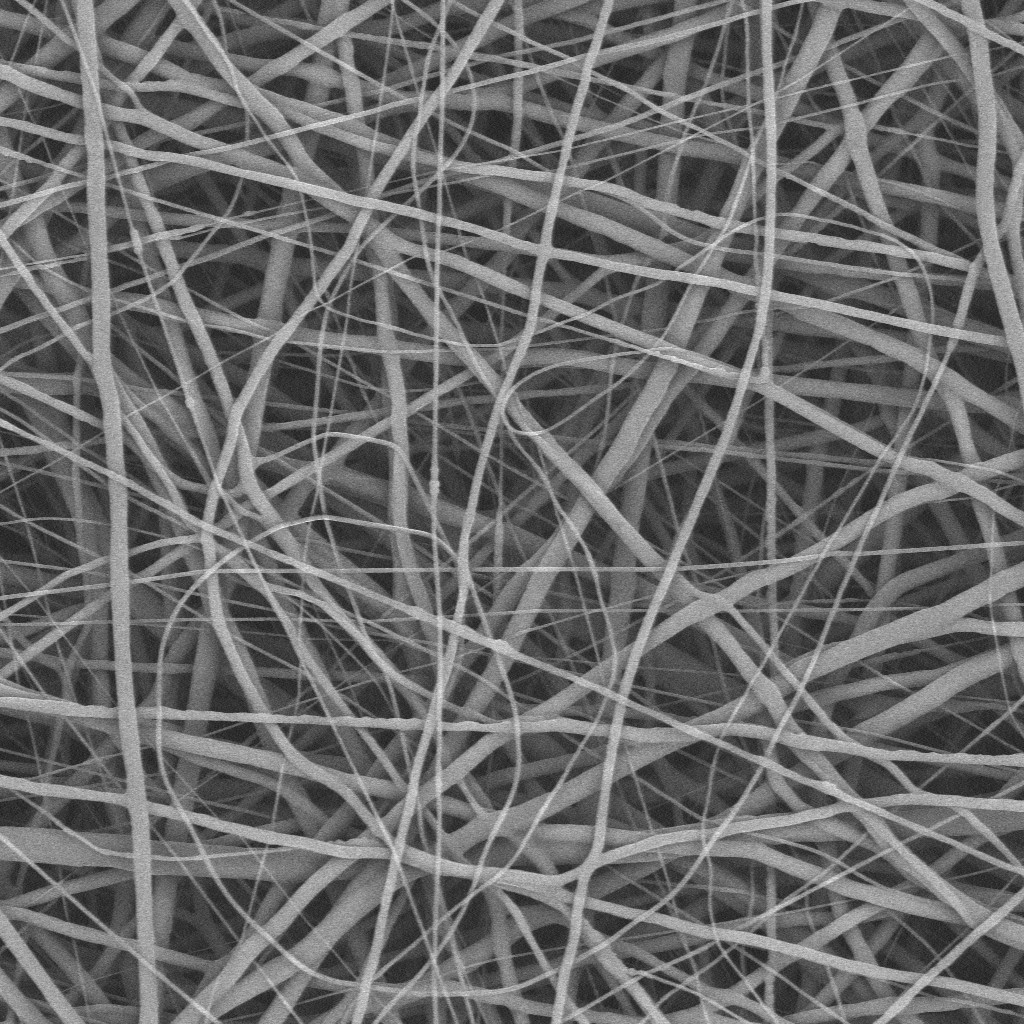

Supplement: S1 Data — (GZ) [file pone.0282903.s002.gz › data/FiberDiameter/Fibermetric for Central area/1,100rpm at 5,000X_20220113164859/Export/Fibermetric_Image0003.jpg]

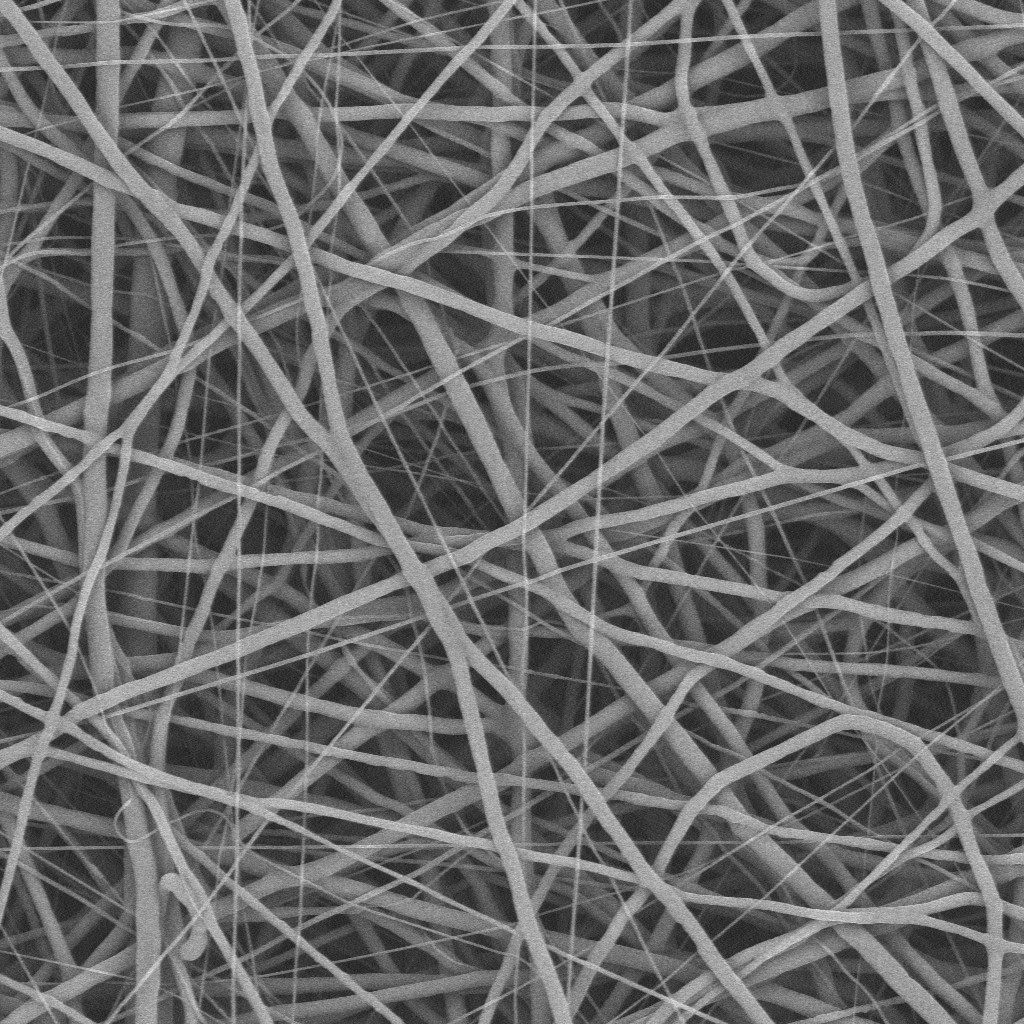

Supplement: S1 Data — (GZ) [file pone.0282903.s002.gz › data/FiberDiameter/Fibermetric for Central area/1,100rpm at 5,000X_20220113164859/Export/Fibermetric_Image0004.jpg]

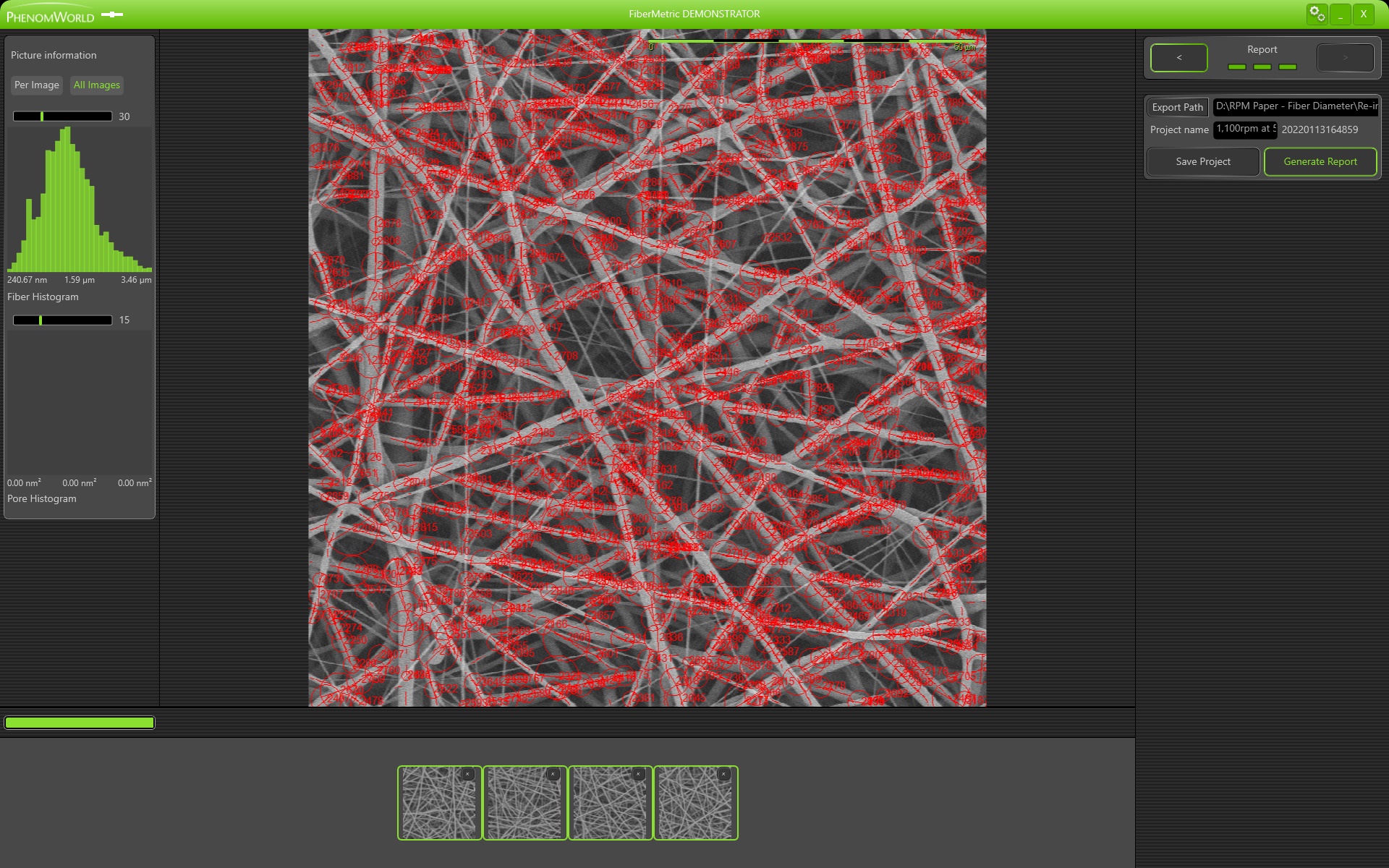

Supplement: S1 Data — (GZ) [file pone.0282903.s002.gz › data/FiberDiameter/Fibermetric for Central area/1,100rpm at 5,000X_20220113164859/Export/Screenshot.jpg]

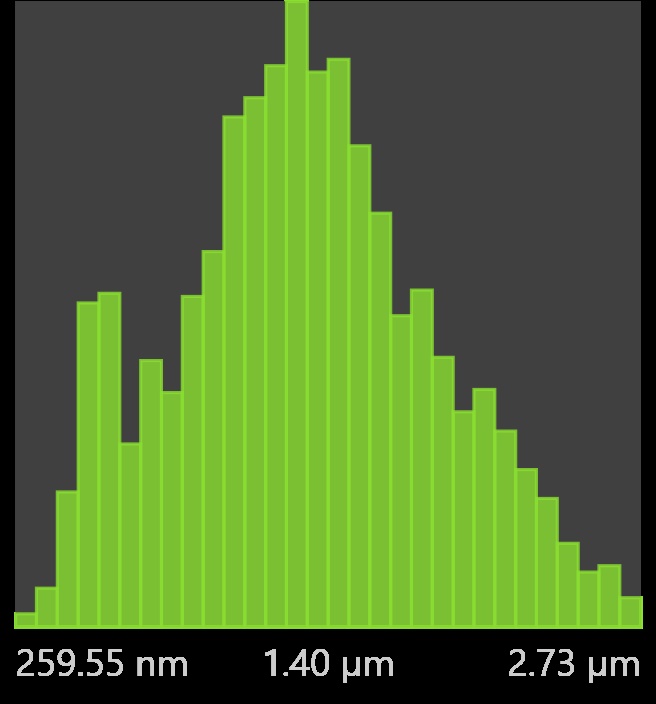

Supplement: S1 Data — (GZ) [file pone.0282903.s002.gz › data/FiberDiameter/Fiber analysis at 5,000X - Edge/Edge, 200rpm at 5,000X/200rpm, Fiber diameter_5,000X__20220127142343/Export/FiberHistogram.jpg]

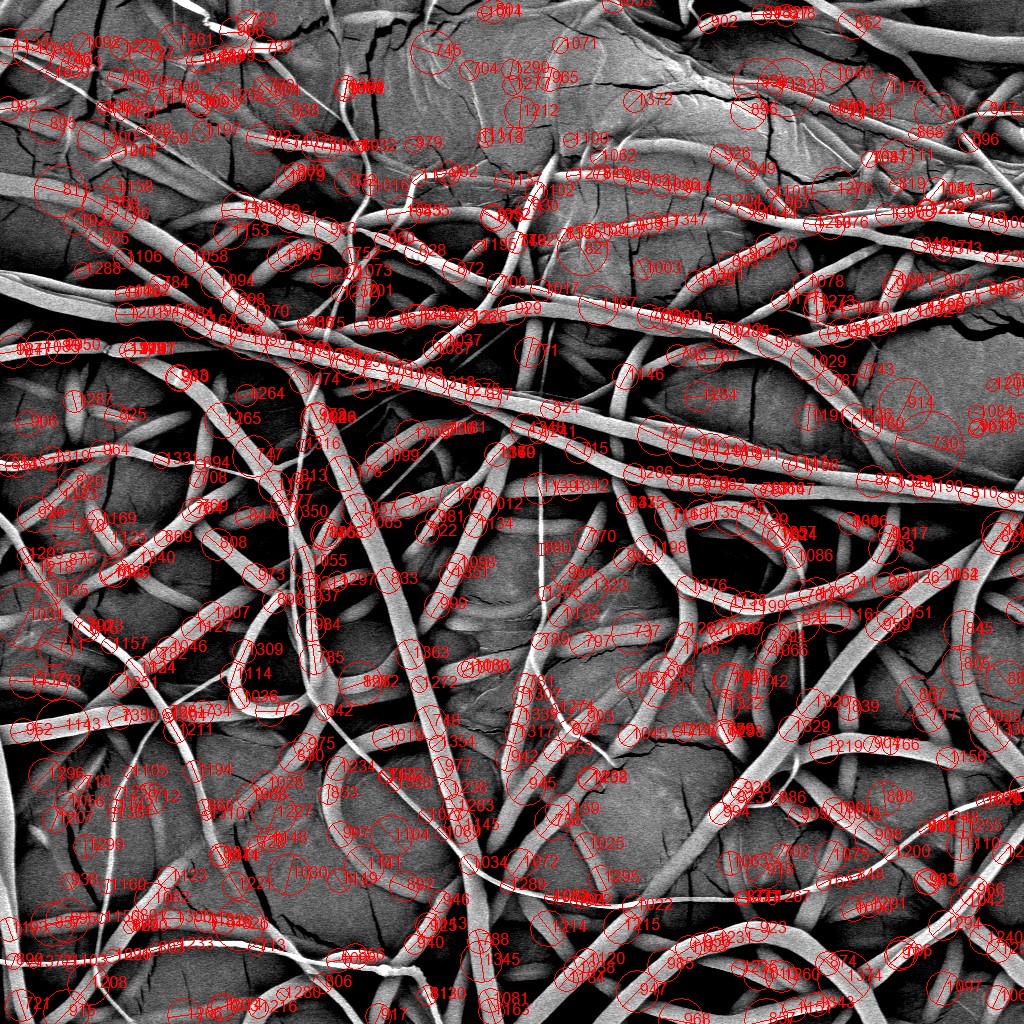

Supplement: S1 Data — (GZ) [file pone.0282903.s002.gz › data/FiberDiameter/Fiber analysis at 5,000X - Edge/Edge, 200rpm at 5,000X/200rpm, Fiber diameter_5,000X__20220127142343/Export/FibermetricImageMeasurements_03ed33e5-908f-4cdd-a6a2-4e8e896bd538.jpg]

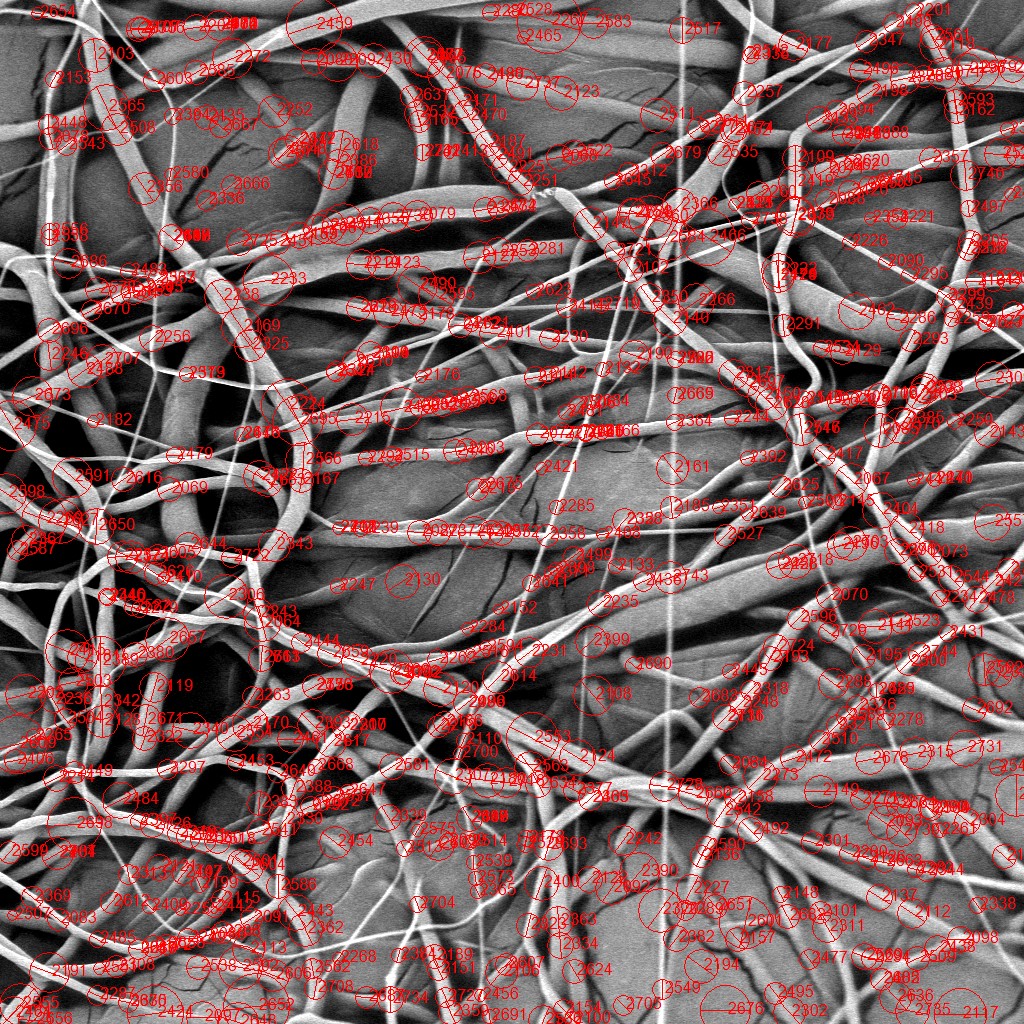

Supplement: S1 Data — (GZ) [file pone.0282903.s002.gz › data/FiberDiameter/Fiber analysis at 5,000X - Edge/Edge, 200rpm at 5,000X/200rpm, Fiber diameter_5,000X__20220127142343/Export/FibermetricImageMeasurements_8e138d37-59f5-4320-b036-56fc05c68e10.jpg]

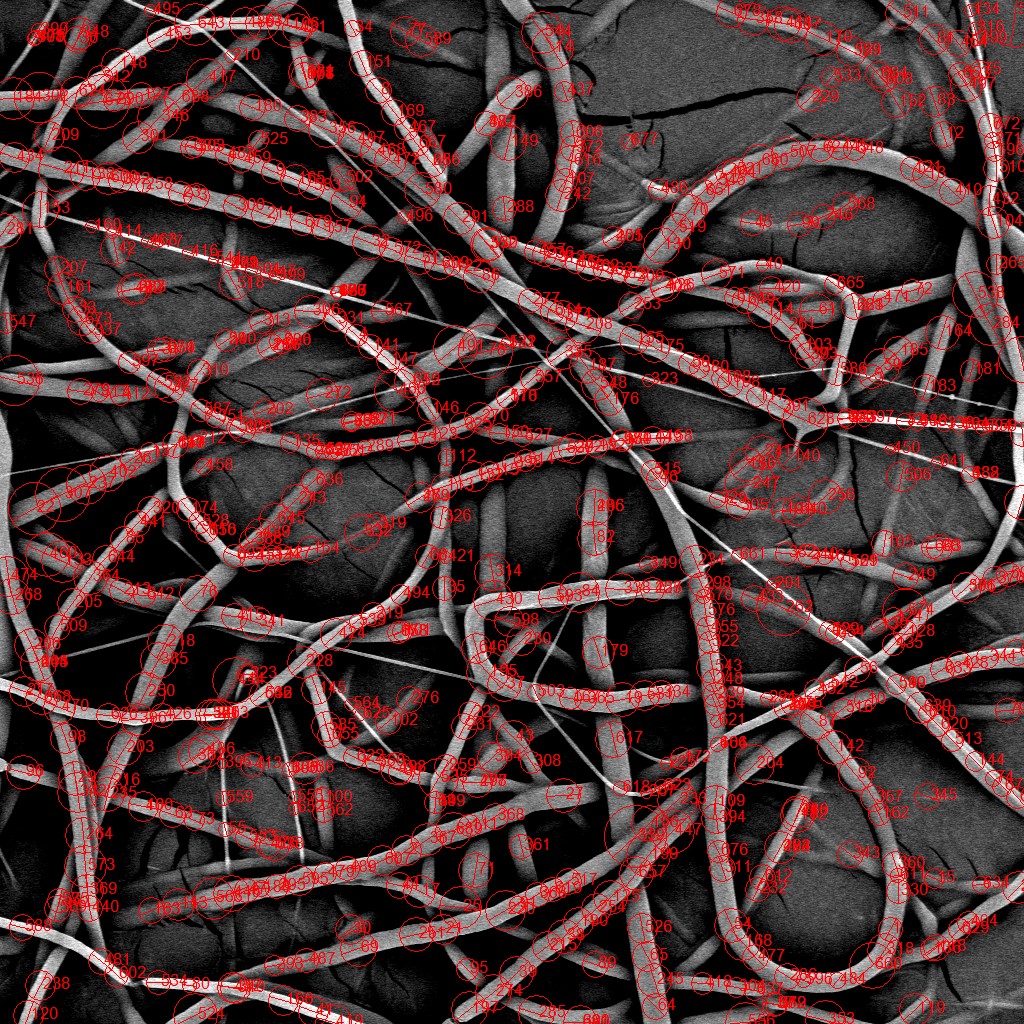

Supplement: S1 Data — (GZ) [file pone.0282903.s002.gz › data/FiberDiameter/Fiber analysis at 5,000X - Edge/Edge, 200rpm at 5,000X/200rpm, Fiber diameter_5,000X__20220127142343/Export/FibermetricImageMeasurements_9467c23d-dcb9-4e33-bdf1-f6a8d81f794c.jpg]

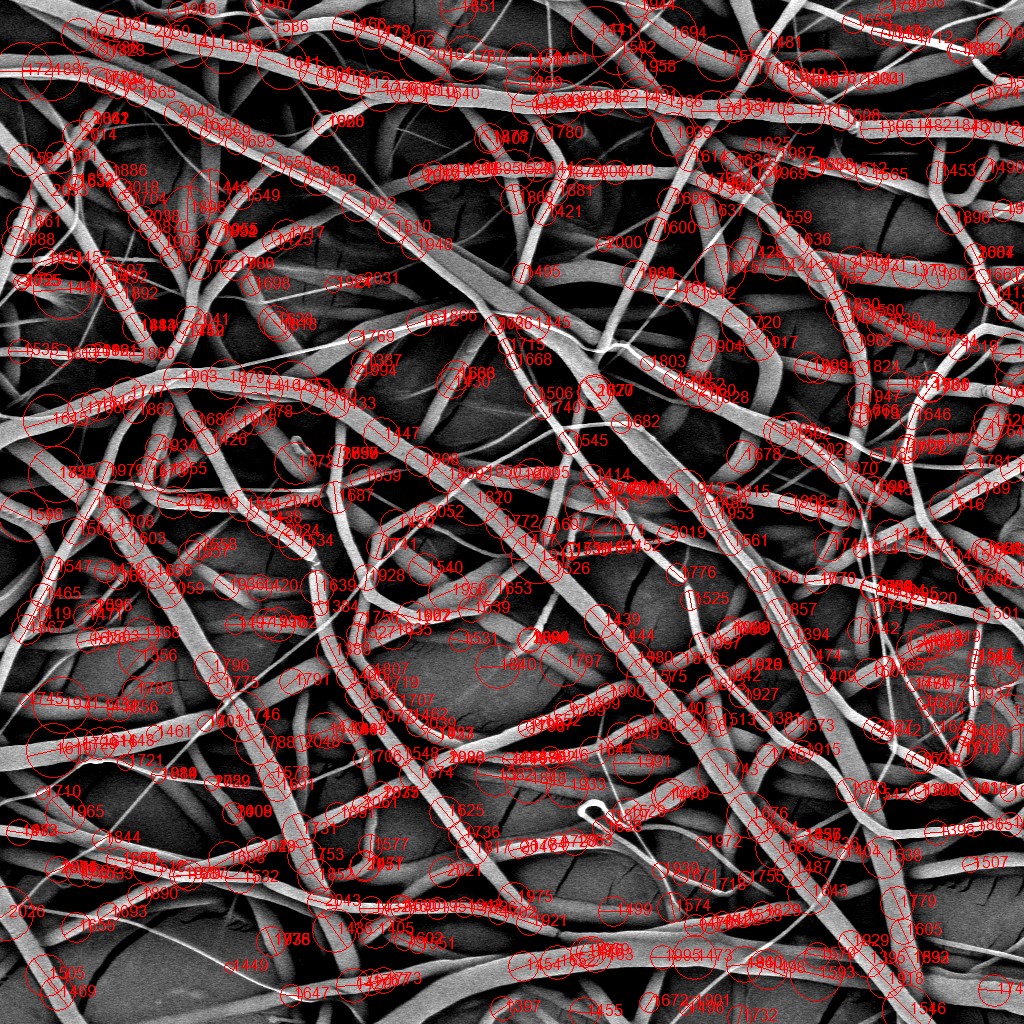

Supplement: S1 Data — (GZ) [file pone.0282903.s002.gz › data/FiberDiameter/Fiber analysis at 5,000X - Edge/Edge, 200rpm at 5,000X/200rpm, Fiber diameter_5,000X__20220127142343/Export/FibermetricImageMeasurements_f5a264c7-10dc-4bd2-8dc5-8ceb5864e378.jpg]

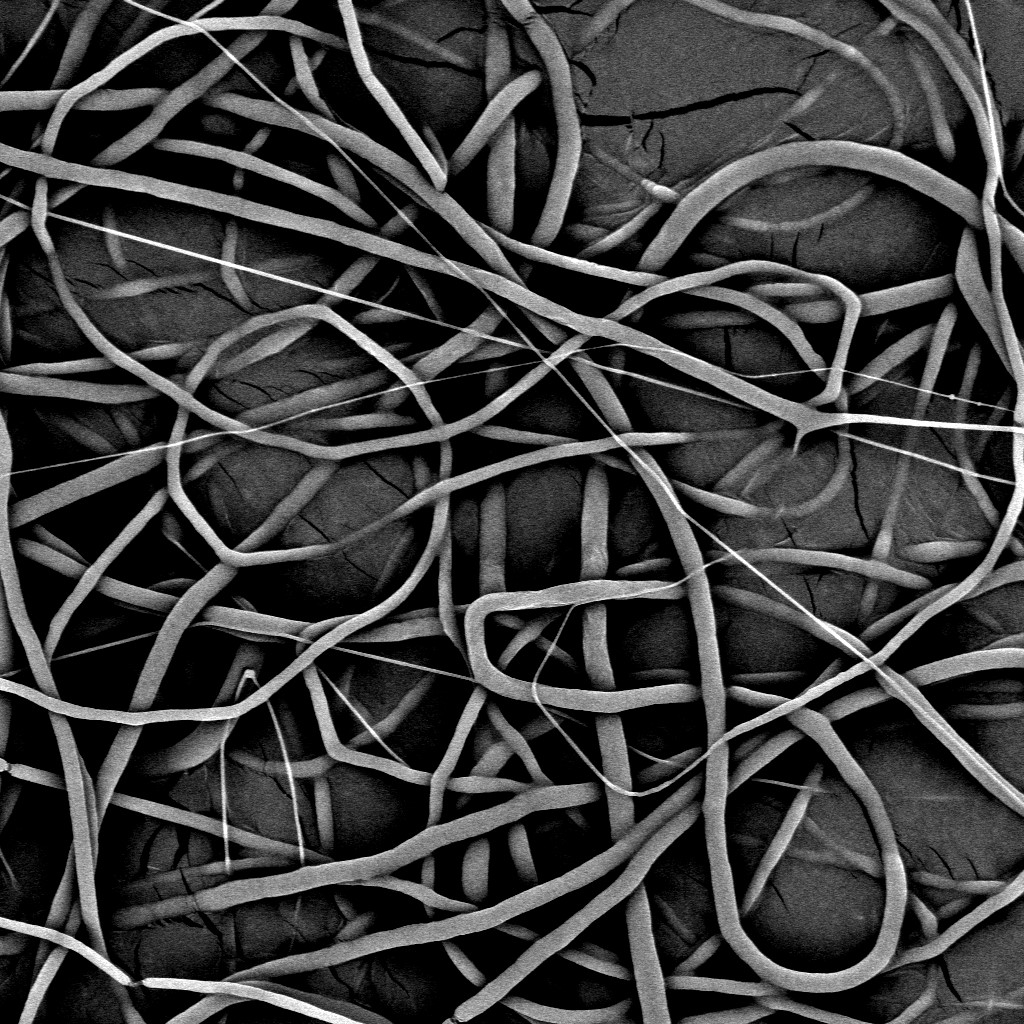

Supplement: S1 Data — (GZ) [file pone.0282903.s002.gz › data/FiberDiameter/Fiber analysis at 5,000X - Edge/Edge, 200rpm at 5,000X/200rpm, Fiber diameter_5,000X__20220127142343/Export/Fibermetric_Image0001.jpg]

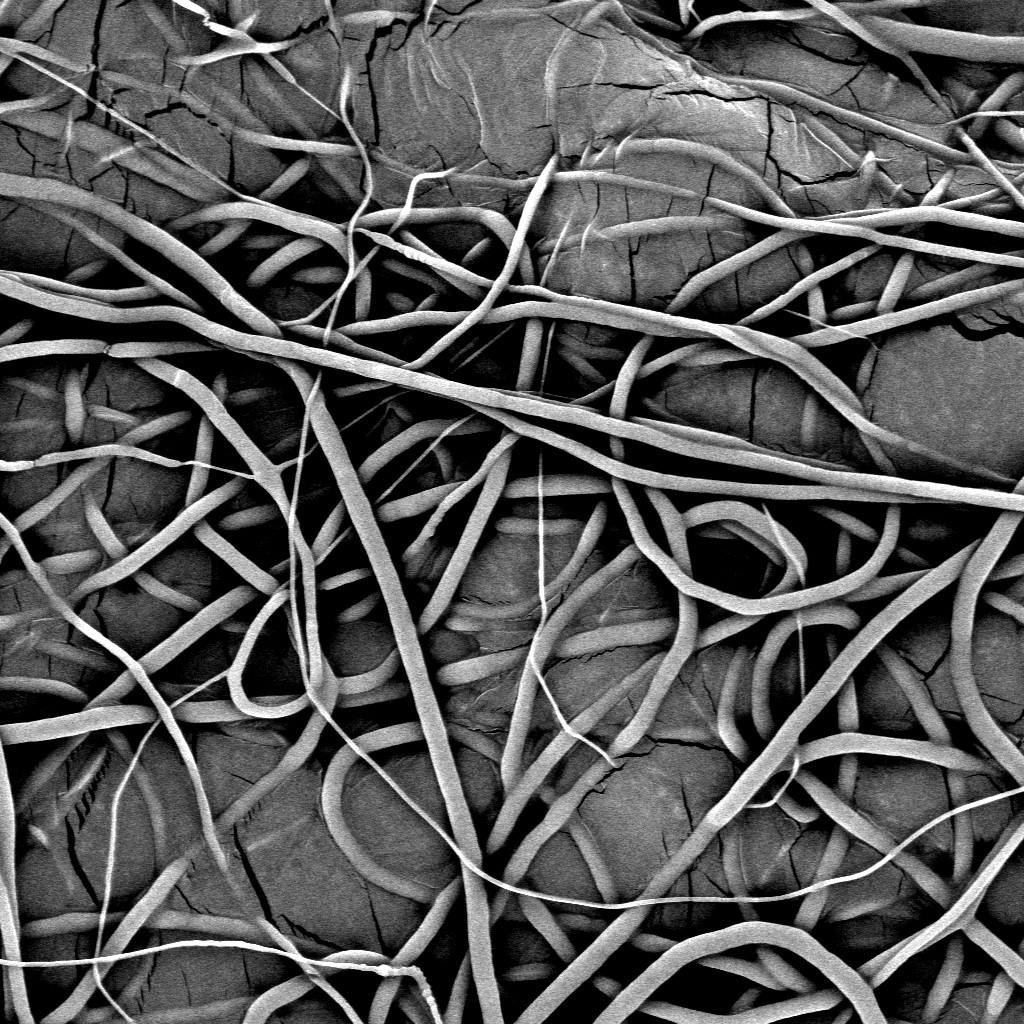

Supplement: S1 Data — (GZ) [file pone.0282903.s002.gz › data/FiberDiameter/Fiber analysis at 5,000X - Edge/Edge, 200rpm at 5,000X/200rpm, Fiber diameter_5,000X__20220127142343/Export/Fibermetric_Image0002.jpg]

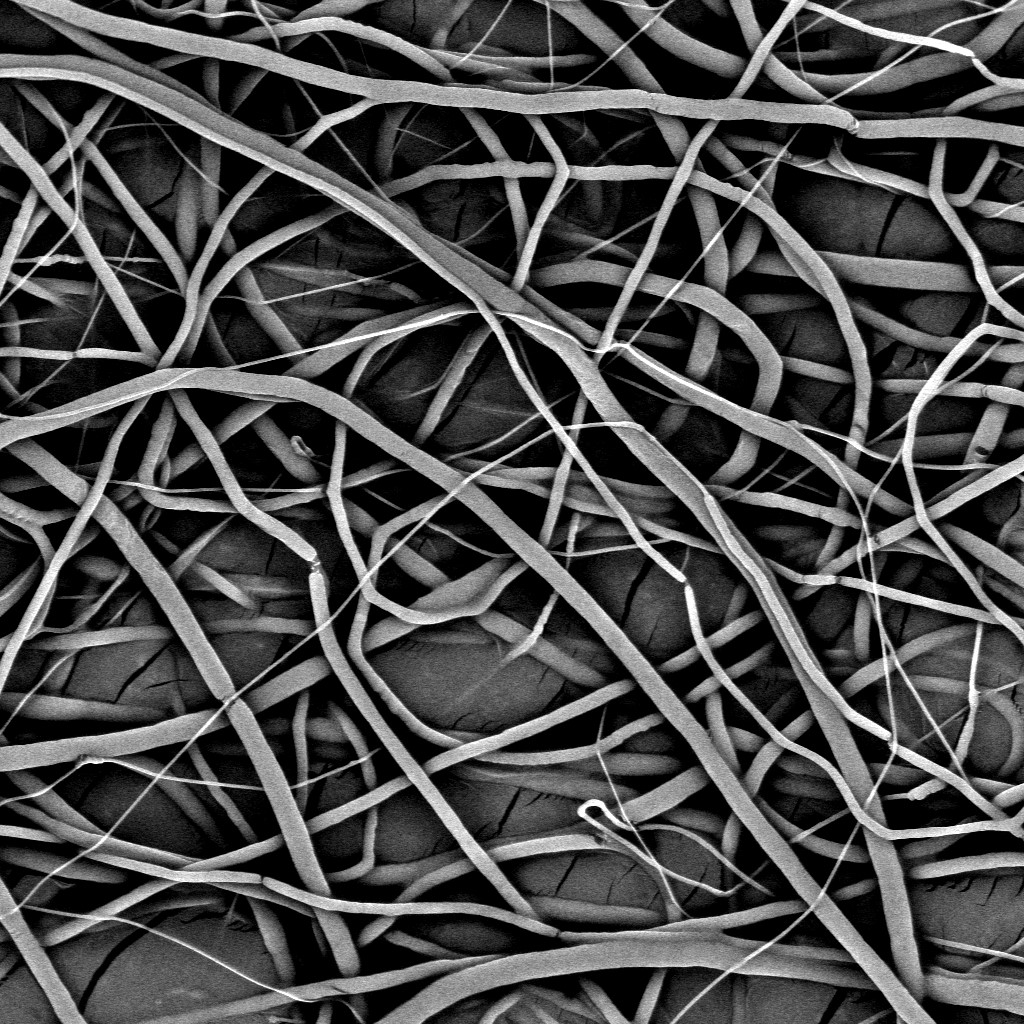

Supplement: S1 Data — (GZ) [file pone.0282903.s002.gz › data/FiberDiameter/Fiber analysis at 5,000X - Edge/Edge, 200rpm at 5,000X/200rpm, Fiber diameter_5,000X__20220127142343/Export/Fibermetric_Image0003.jpg]

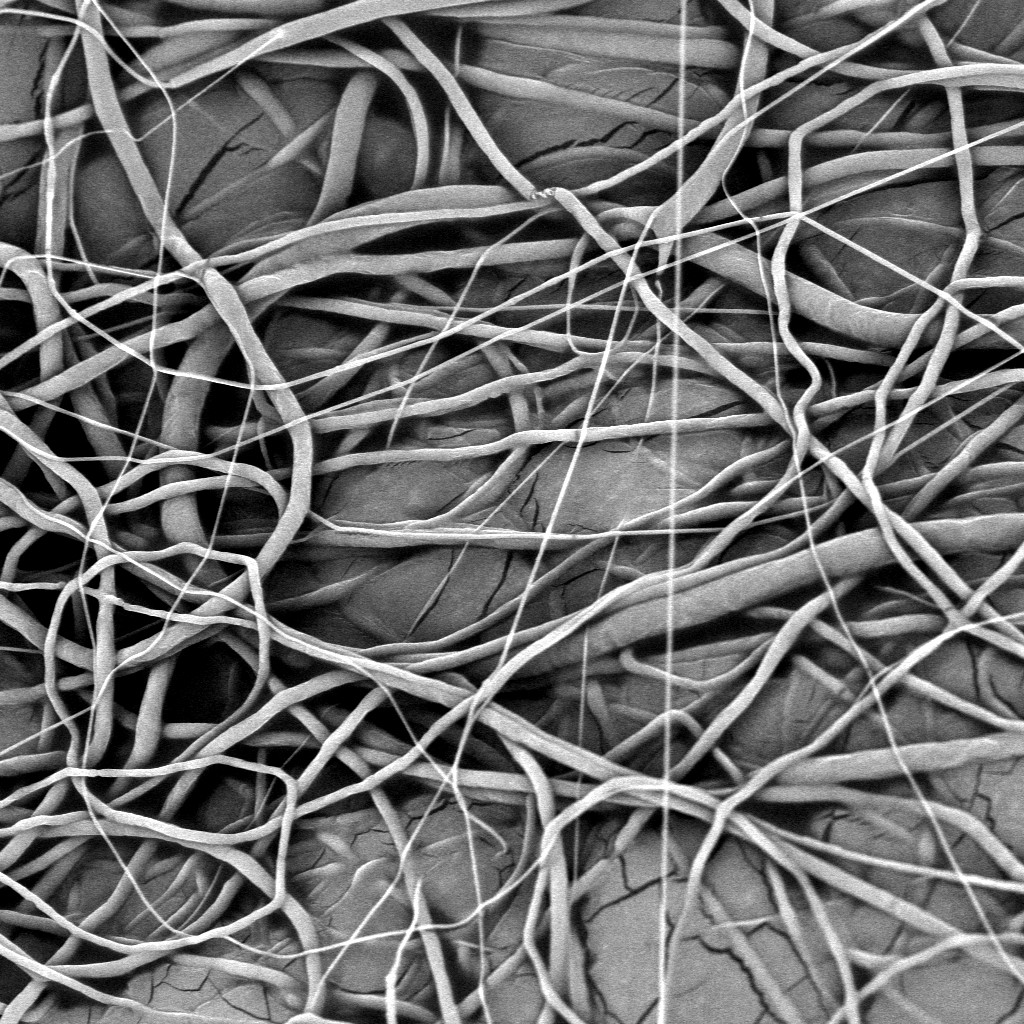

Supplement: S1 Data — (GZ) [file pone.0282903.s002.gz › data/FiberDiameter/Fiber analysis at 5,000X - Edge/Edge, 200rpm at 5,000X/200rpm, Fiber diameter_5,000X__20220127142343/Export/Fibermetric_Image0004.jpg]

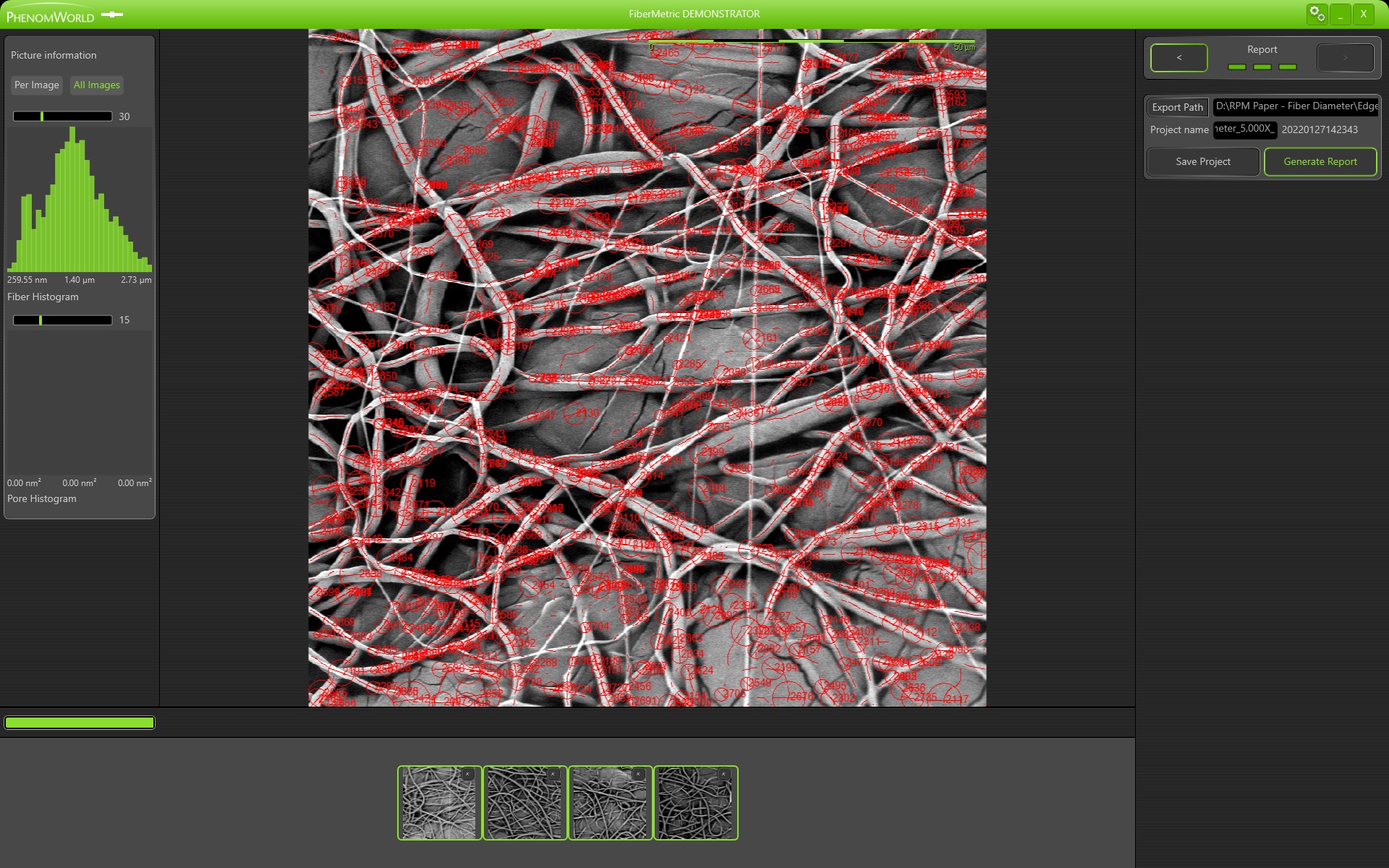

Supplement: S1 Data — (GZ) [file pone.0282903.s002.gz › data/FiberDiameter/Fiber analysis at 5,000X - Edge/Edge, 200rpm at 5,000X/200rpm, Fiber diameter_5,000X__20220127142343/Export/Screenshot.jpg]

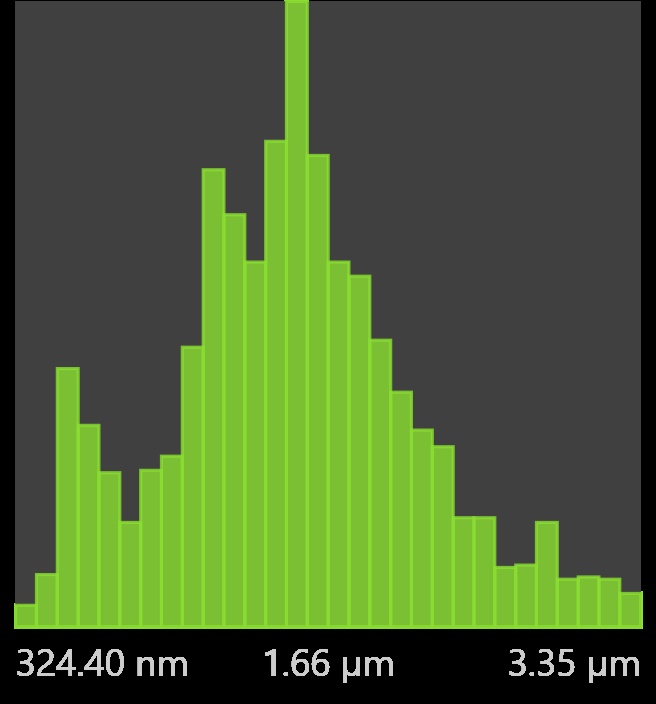

Supplement: S1 Data — (GZ) [file pone.0282903.s002.gz › data/FiberDiameter/Fiber analysis at 5,000X - Edge/Edge, 2,000rpm at 5,000X/2,000rpm, Fiber diameter_5,000X__20220127143455/Export/FiberHistogram.jpg]

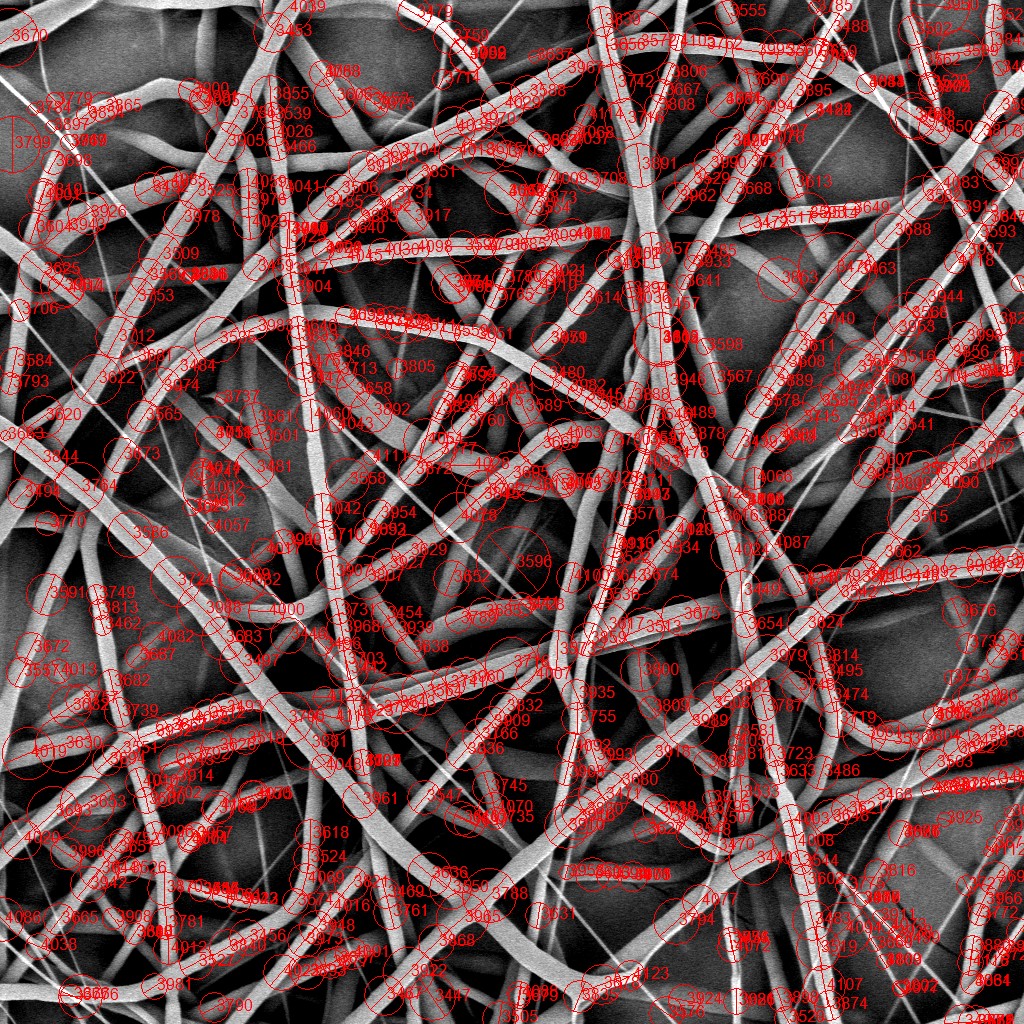

Supplement: S1 Data — (GZ) [file pone.0282903.s002.gz › data/FiberDiameter/Fiber analysis at 5,000X - Edge/Edge, 2,000rpm at 5,000X/2,000rpm, Fiber diameter_5,000X__20220127143455/Export/FibermetricImageMeasurements_1f10c811-fa5d-4863-9518-eafe9a8ea9d9.jpg]

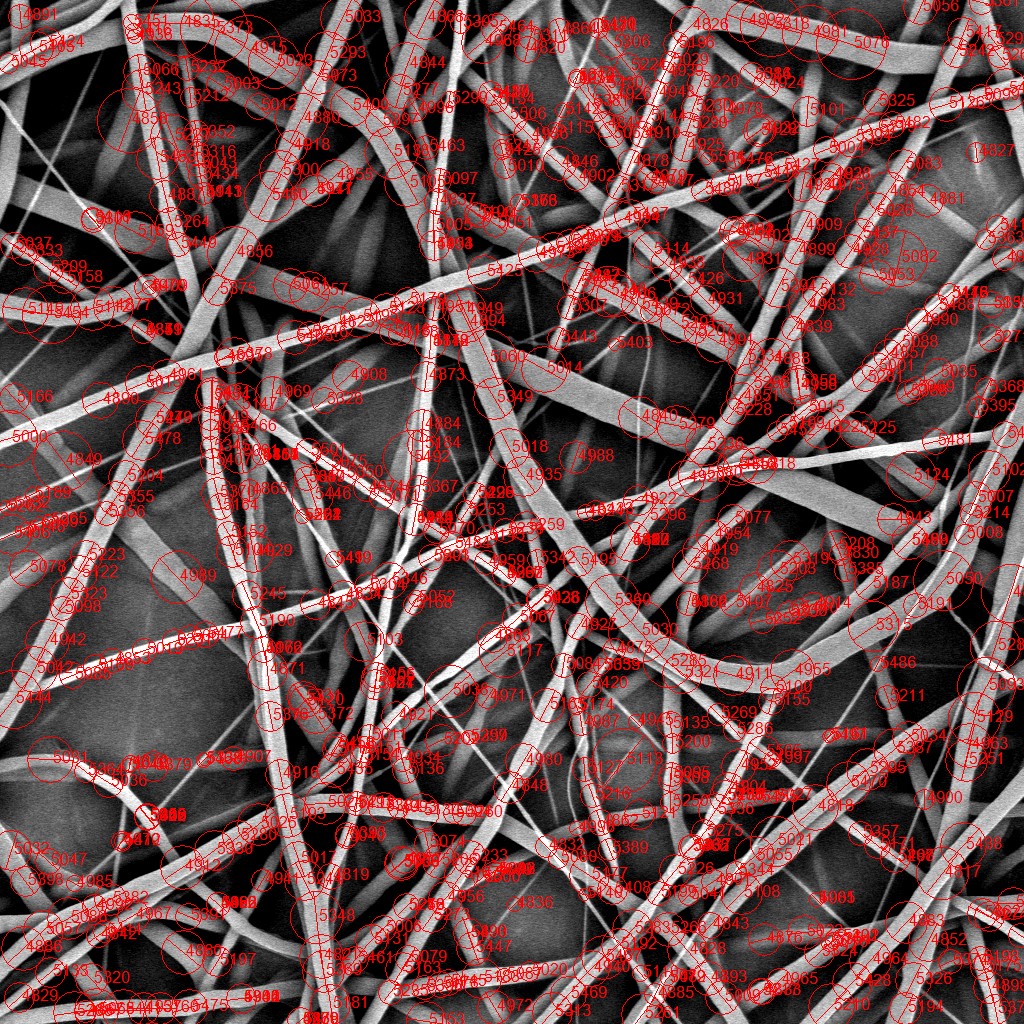

Supplement: S1 Data — (GZ) [file pone.0282903.s002.gz › data/FiberDiameter/Fiber analysis at 5,000X - Edge/Edge, 2,000rpm at 5,000X/2,000rpm, Fiber diameter_5,000X__20220127143455/Export/FibermetricImageMeasurements_27bbf9e3-5c04-4ef3-a232-a280e4757164.jpg]

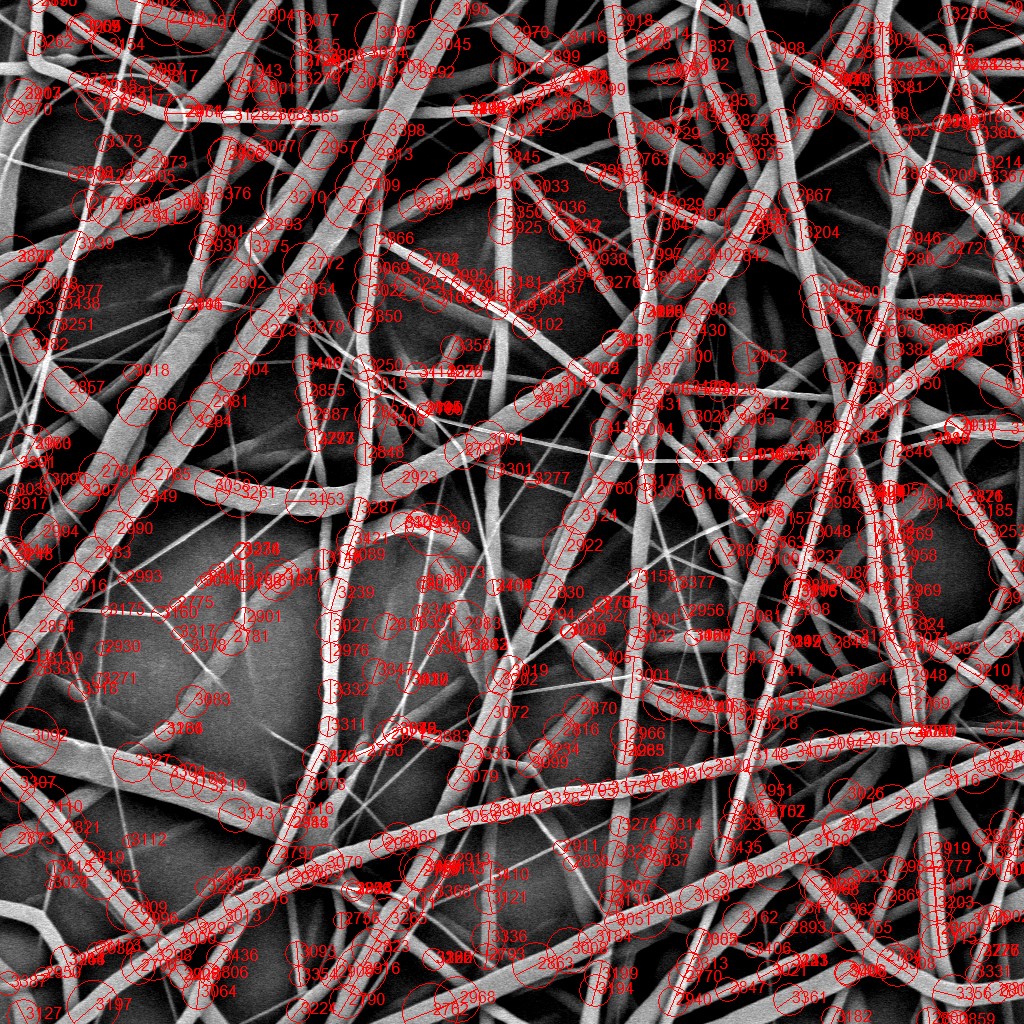

Supplement: S1 Data — (GZ) [file pone.0282903.s002.gz › data/FiberDiameter/Fiber analysis at 5,000X - Edge/Edge, 2,000rpm at 5,000X/2,000rpm, Fiber diameter_5,000X__20220127143455/Export/FibermetricImageMeasurements_9b6ea457-36cd-4d61-a2d4-057b66584d61.jpg]

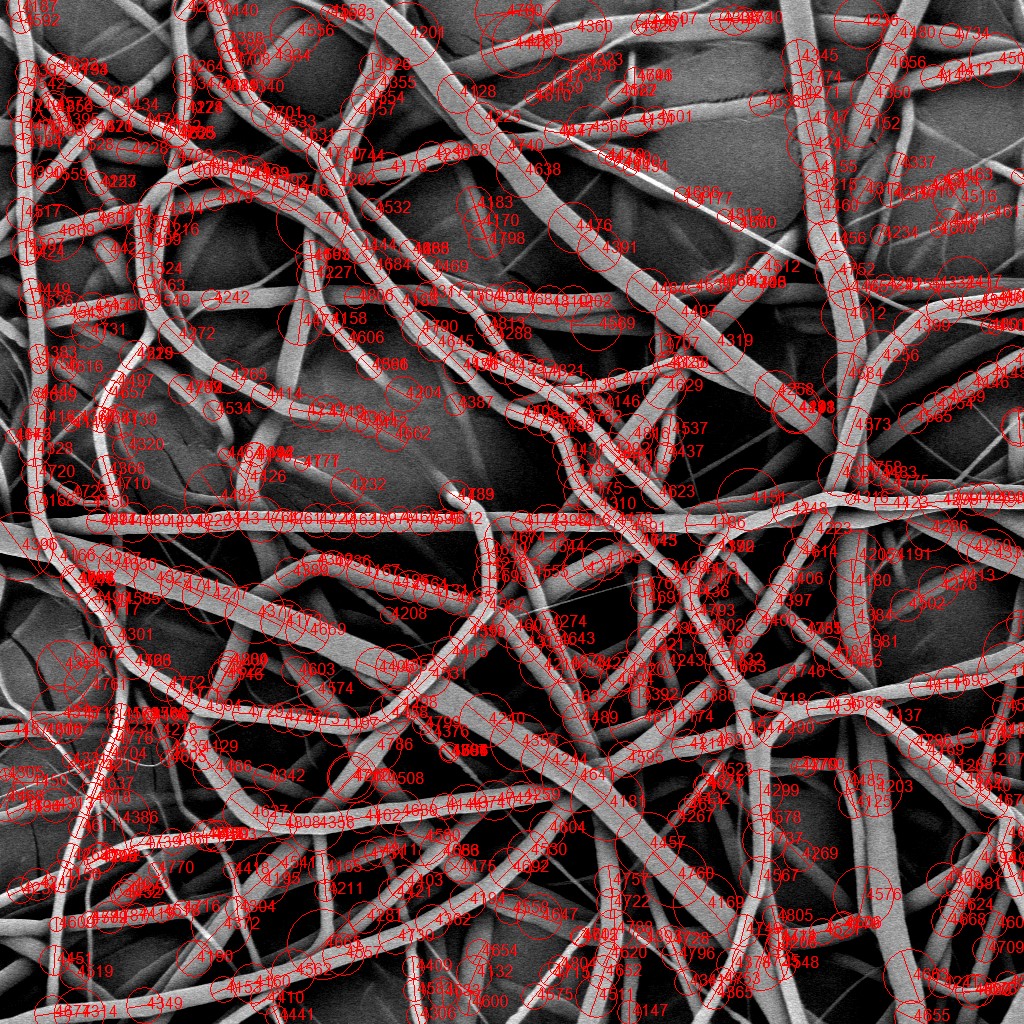

Supplement: S1 Data — (GZ) [file pone.0282903.s002.gz › data/FiberDiameter/Fiber analysis at 5,000X - Edge/Edge, 2,000rpm at 5,000X/2,000rpm, Fiber diameter_5,000X__20220127143455/Export/FibermetricImageMeasurements_a475a77f-53c3-4e46-9213-d1904de50c7d.jpg]

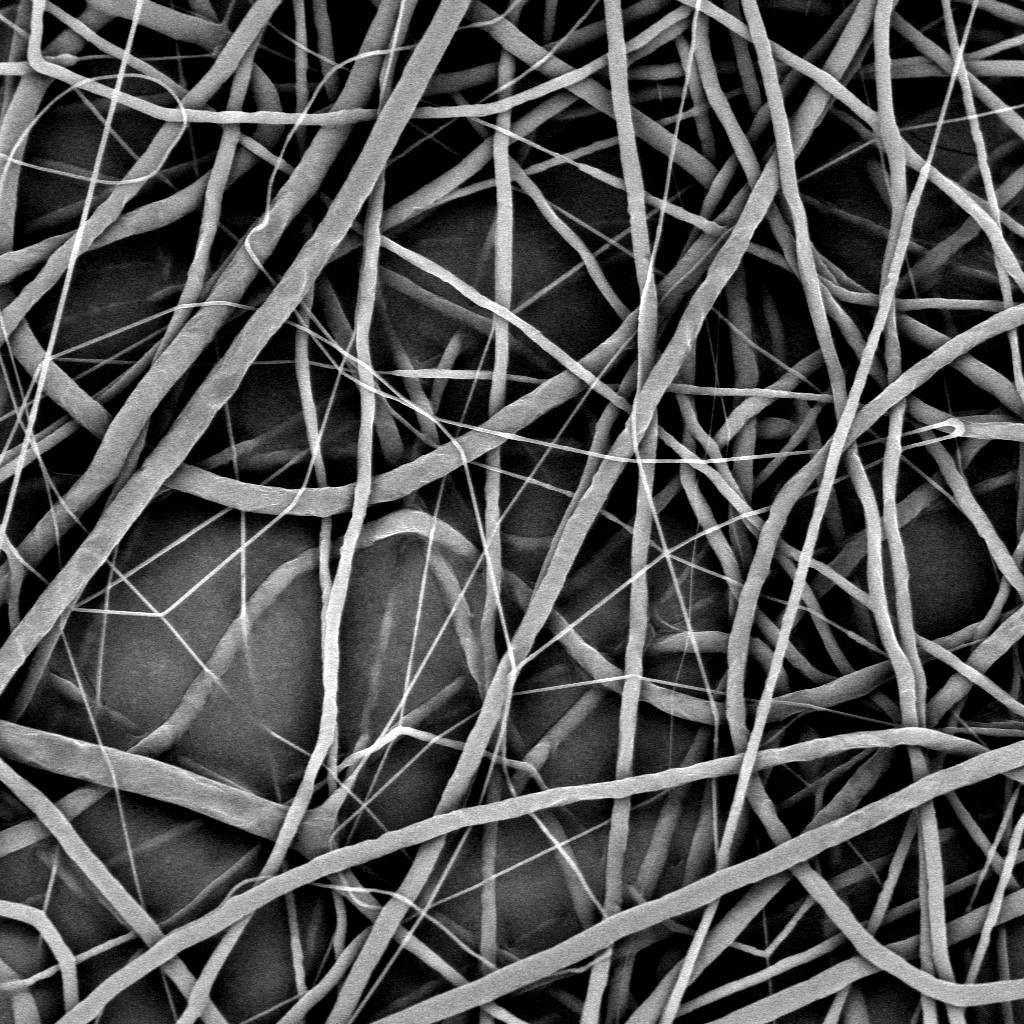

Supplement: S1 Data — (GZ) [file pone.0282903.s002.gz › data/FiberDiameter/Fiber analysis at 5,000X - Edge/Edge, 2,000rpm at 5,000X/2,000rpm, Fiber diameter_5,000X__20220127143455/Export/Fibermetric_Image0001.jpg]

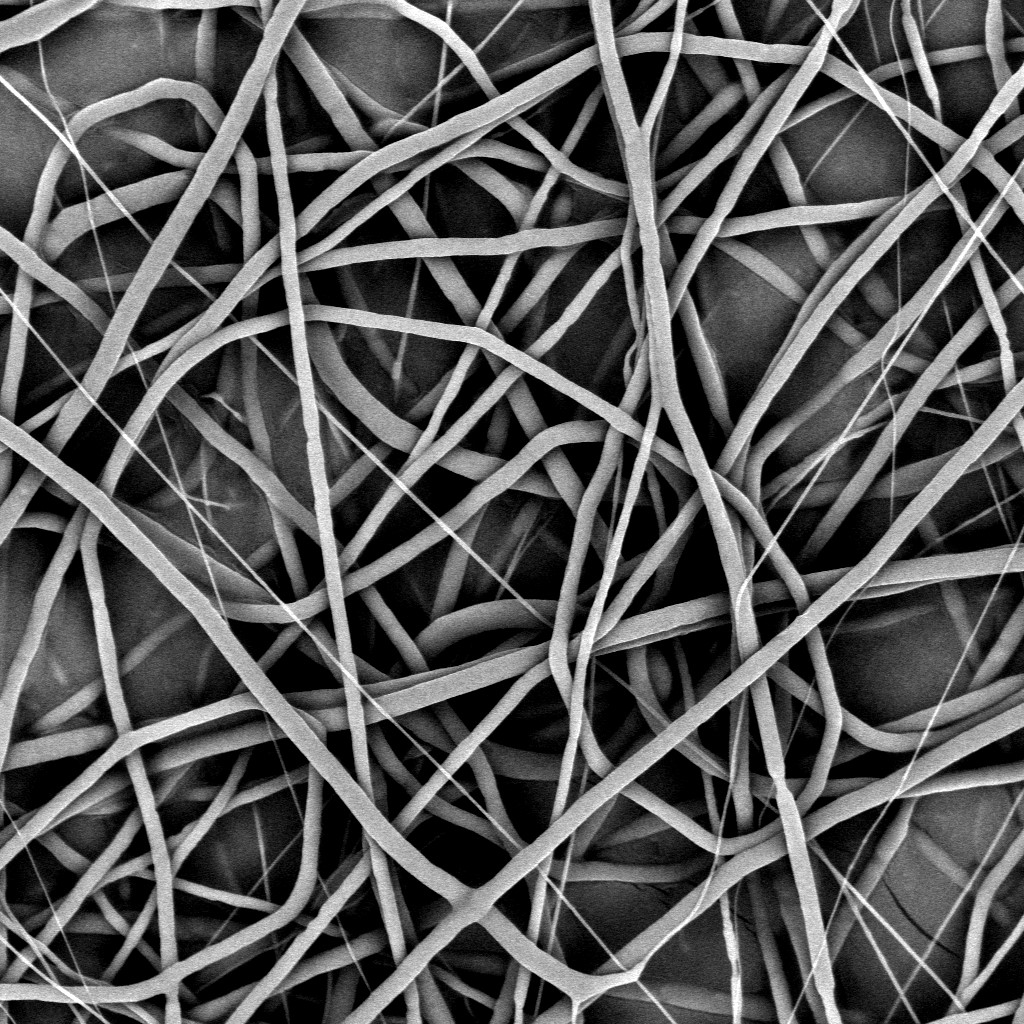

Supplement: S1 Data — (GZ) [file pone.0282903.s002.gz › data/FiberDiameter/Fiber analysis at 5,000X - Edge/Edge, 2,000rpm at 5,000X/2,000rpm, Fiber diameter_5,000X__20220127143455/Export/Fibermetric_Image0002.jpg]

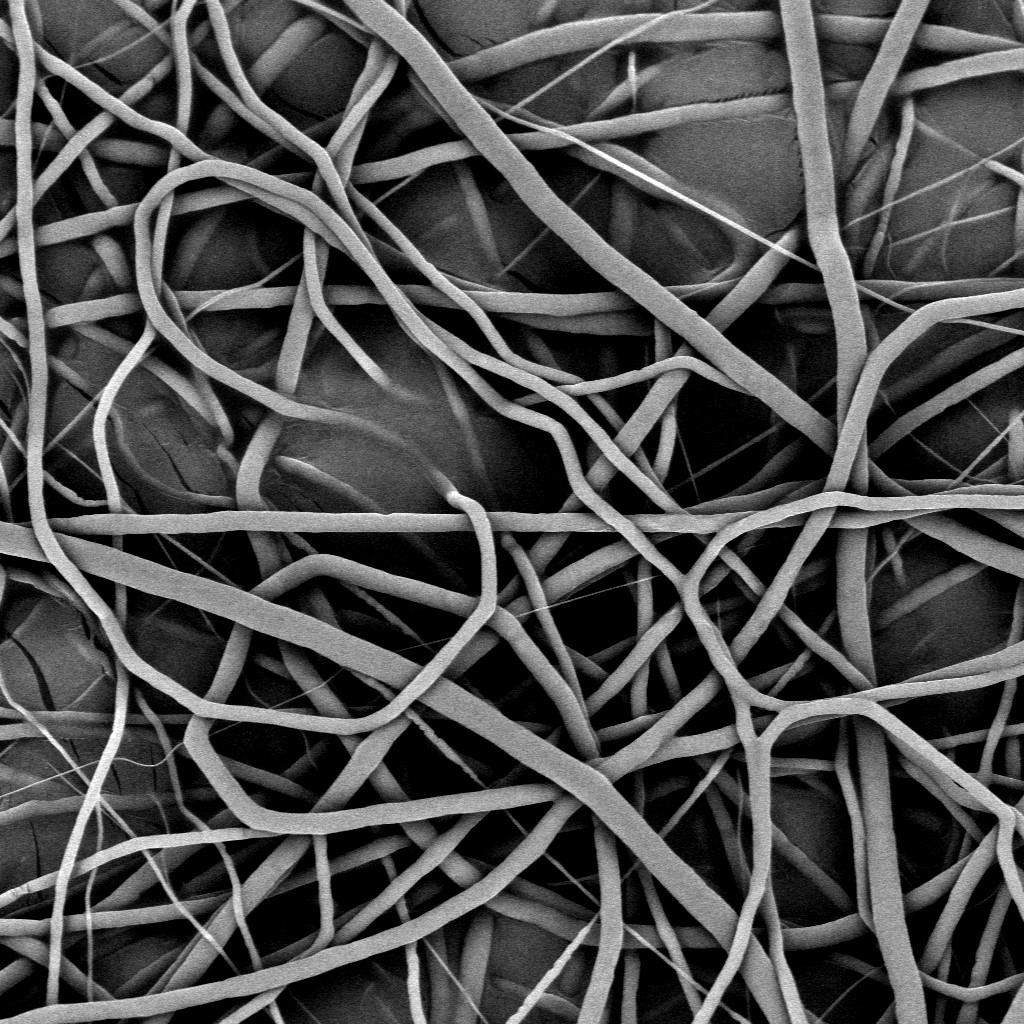

Supplement: S1 Data — (GZ) [file pone.0282903.s002.gz › data/FiberDiameter/Fiber analysis at 5,000X - Edge/Edge, 2,000rpm at 5,000X/2,000rpm, Fiber diameter_5,000X__20220127143455/Export/Fibermetric_Image0003.jpg]

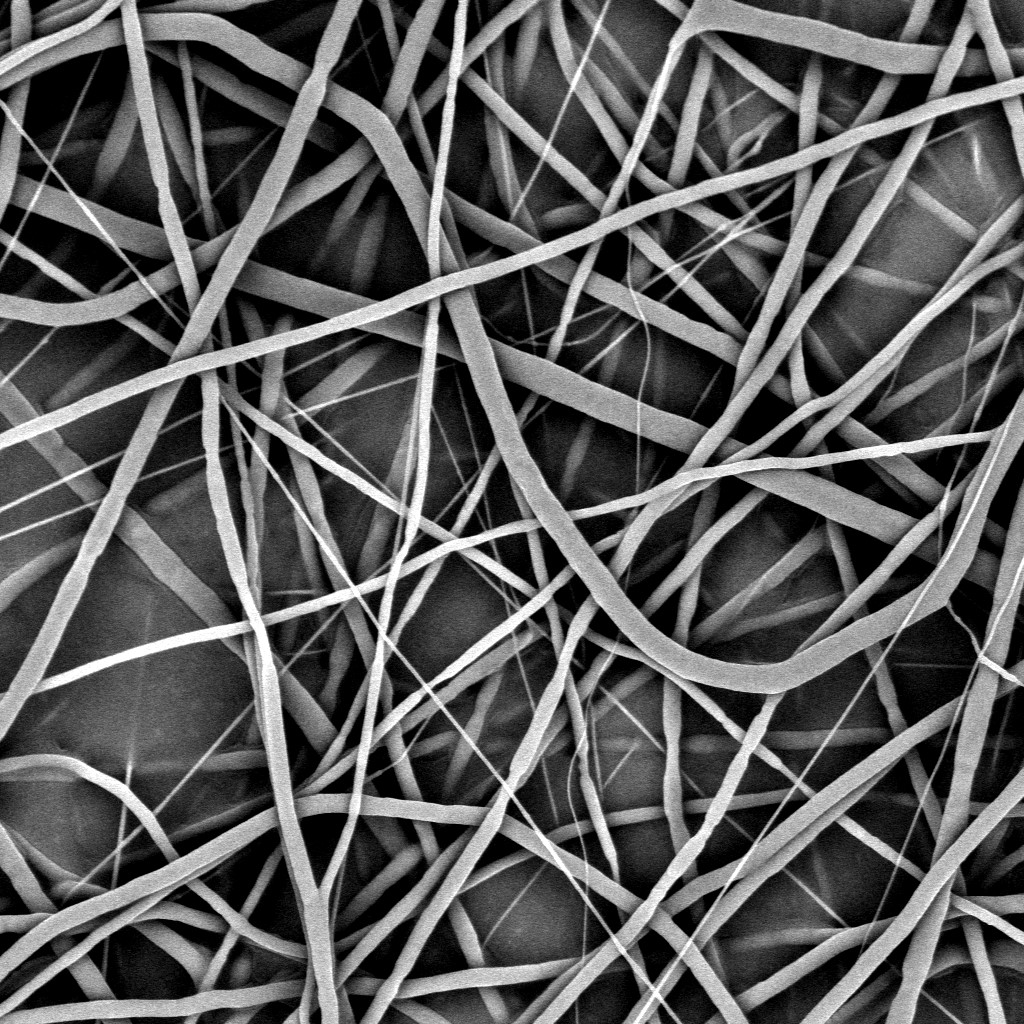

Supplement: S1 Data — (GZ) [file pone.0282903.s002.gz › data/FiberDiameter/Fiber analysis at 5,000X - Edge/Edge, 2,000rpm at 5,000X/2,000rpm, Fiber diameter_5,000X__20220127143455/Export/Fibermetric_Image0004.jpg]

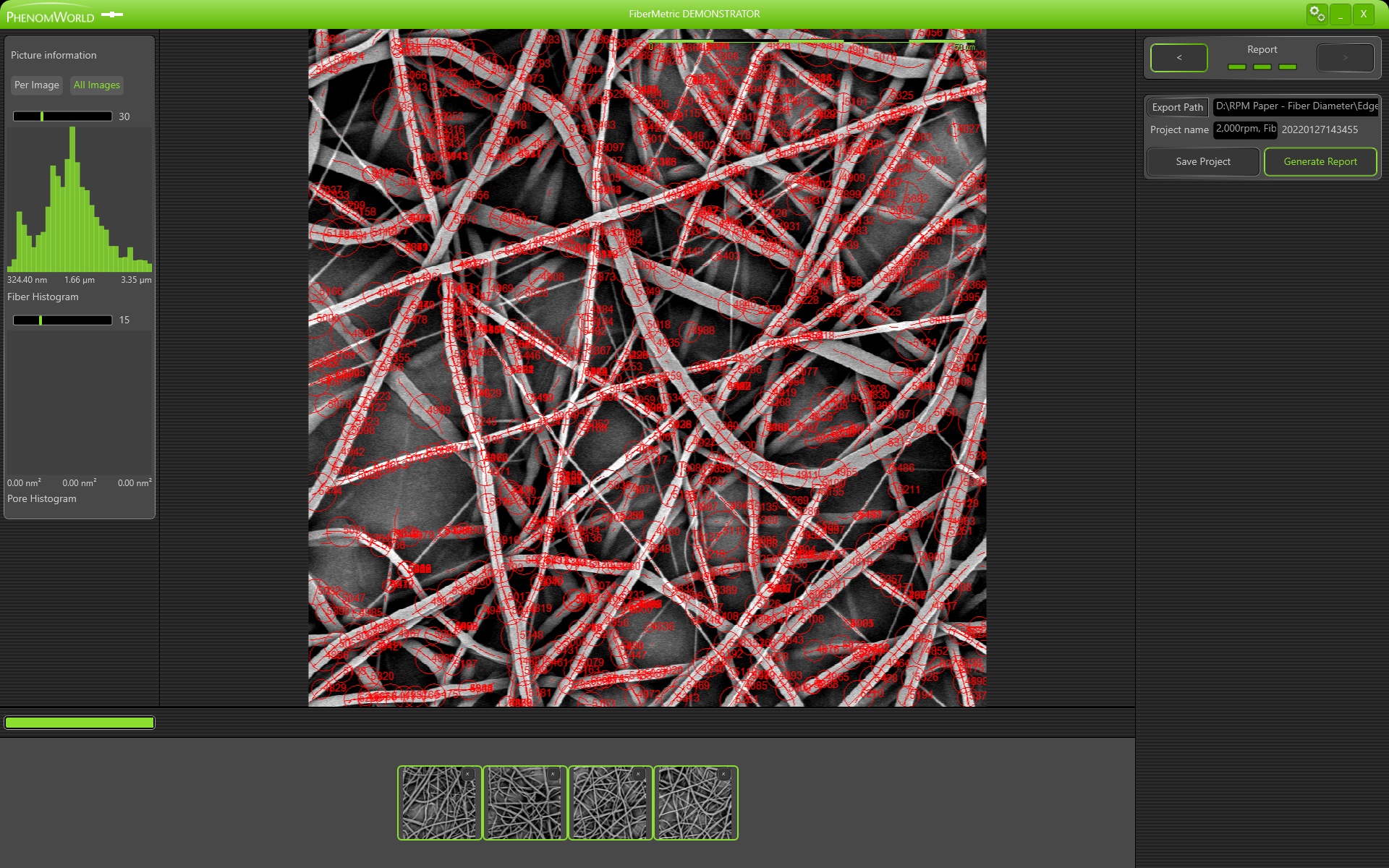

Supplement: S1 Data — (GZ) [file pone.0282903.s002.gz › data/FiberDiameter/Fiber analysis at 5,000X - Edge/Edge, 2,000rpm at 5,000X/2,000rpm, Fiber diameter_5,000X__20220127143455/Export/Screenshot.jpg]

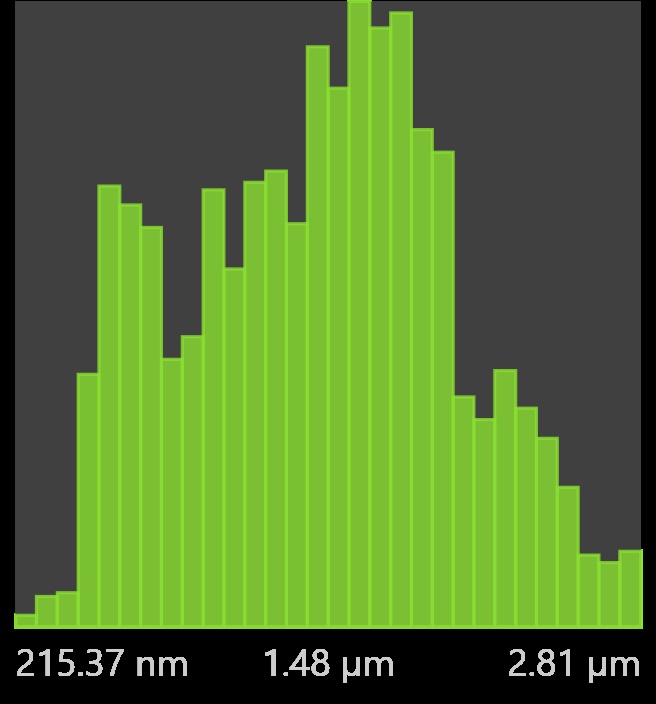

Supplement: S1 Data — (GZ) [file pone.0282903.s002.gz › data/FiberDiameter/Fiber analysis at 5,000X - Edge/Edge, 1,100rpm at 5,000X/1,100rpm, Fiber diameter_5,000X__20220127142849/Export/FiberHistogram.jpg]

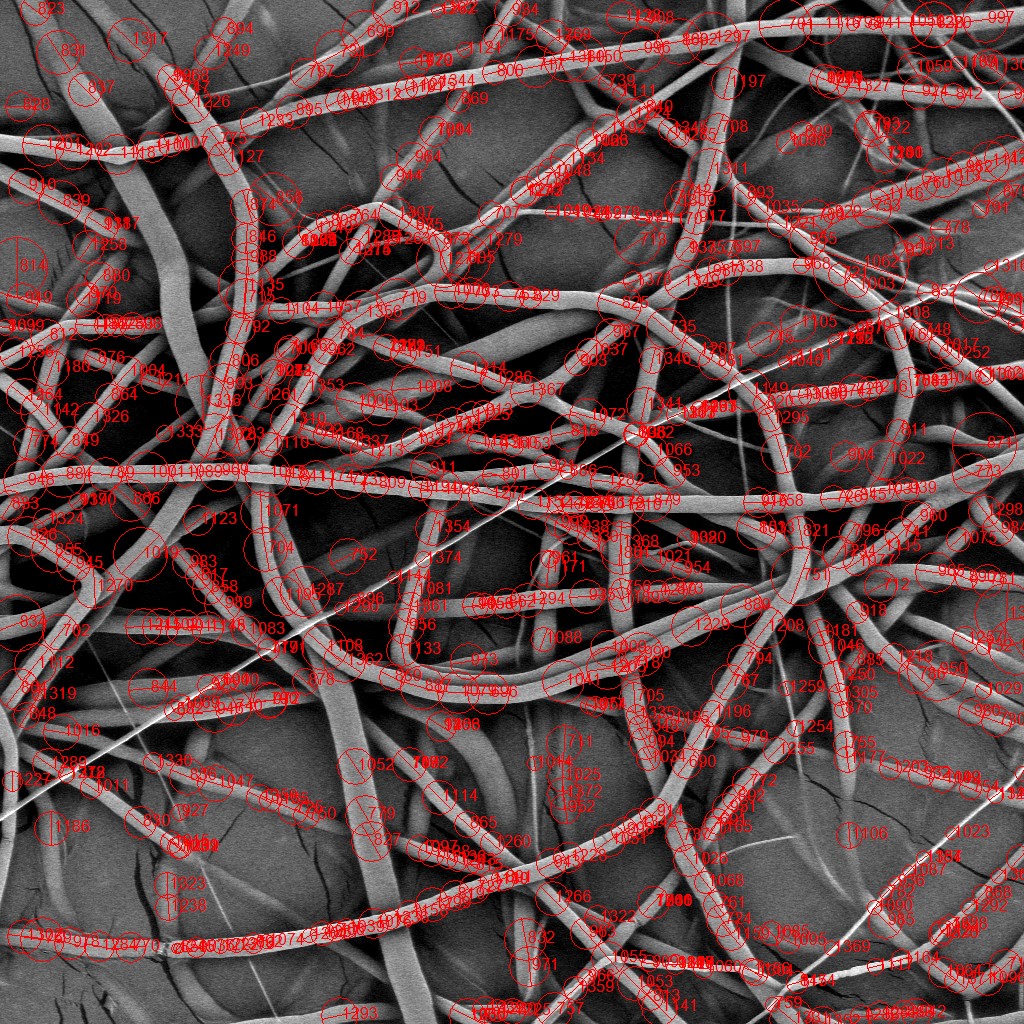

Supplement: S1 Data — (GZ) [file pone.0282903.s002.gz › data/FiberDiameter/Fiber analysis at 5,000X - Edge/Edge, 1,100rpm at 5,000X/1,100rpm, Fiber diameter_5,000X__20220127142849/Export/FibermetricImageMeasurements_23c9bca8-3f68-44c9-954b-59d1b09c1932.jpg]

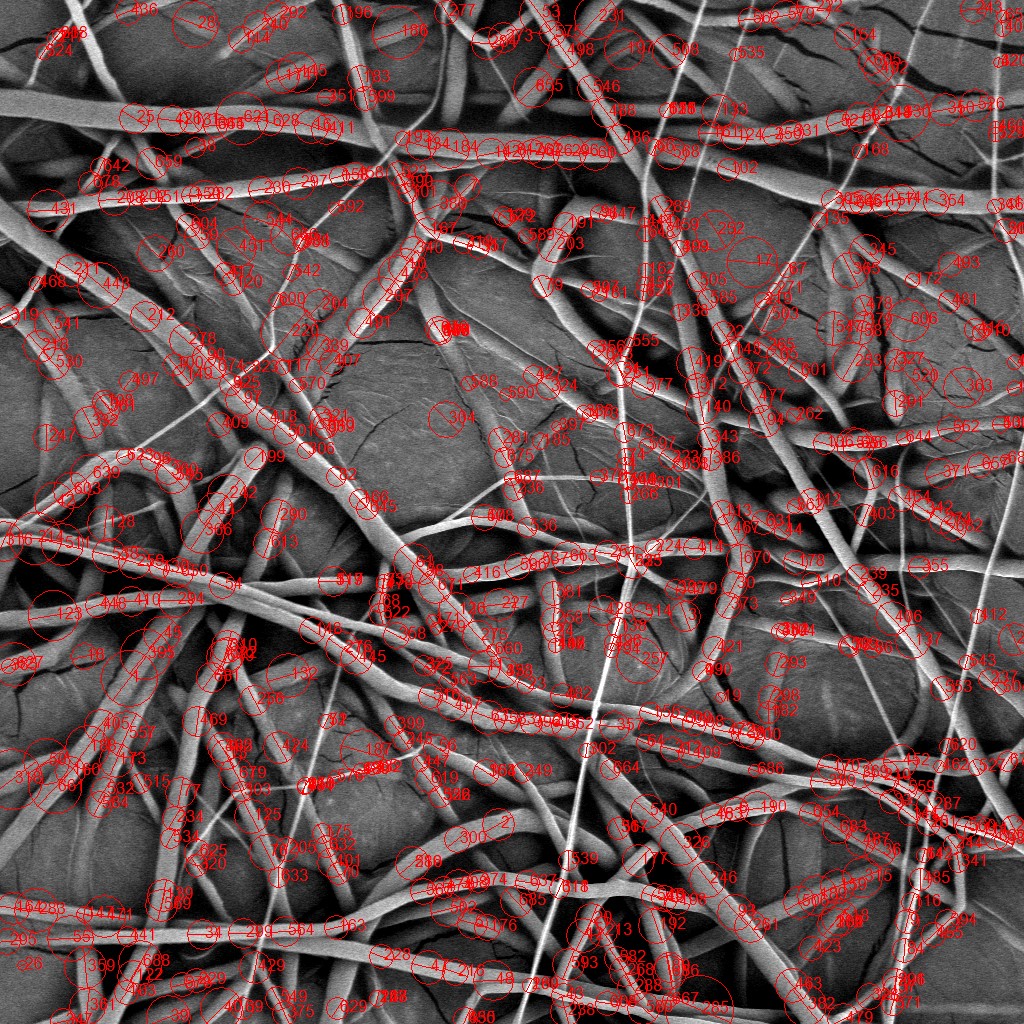

Supplement: S1 Data — (GZ) [file pone.0282903.s002.gz › data/FiberDiameter/Fiber analysis at 5,000X - Edge/Edge, 1,100rpm at 5,000X/1,100rpm, Fiber diameter_5,000X__20220127142849/Export/FibermetricImageMeasurements_860fc463-e070-4ee1-94da-37119e5b6058.jpg]

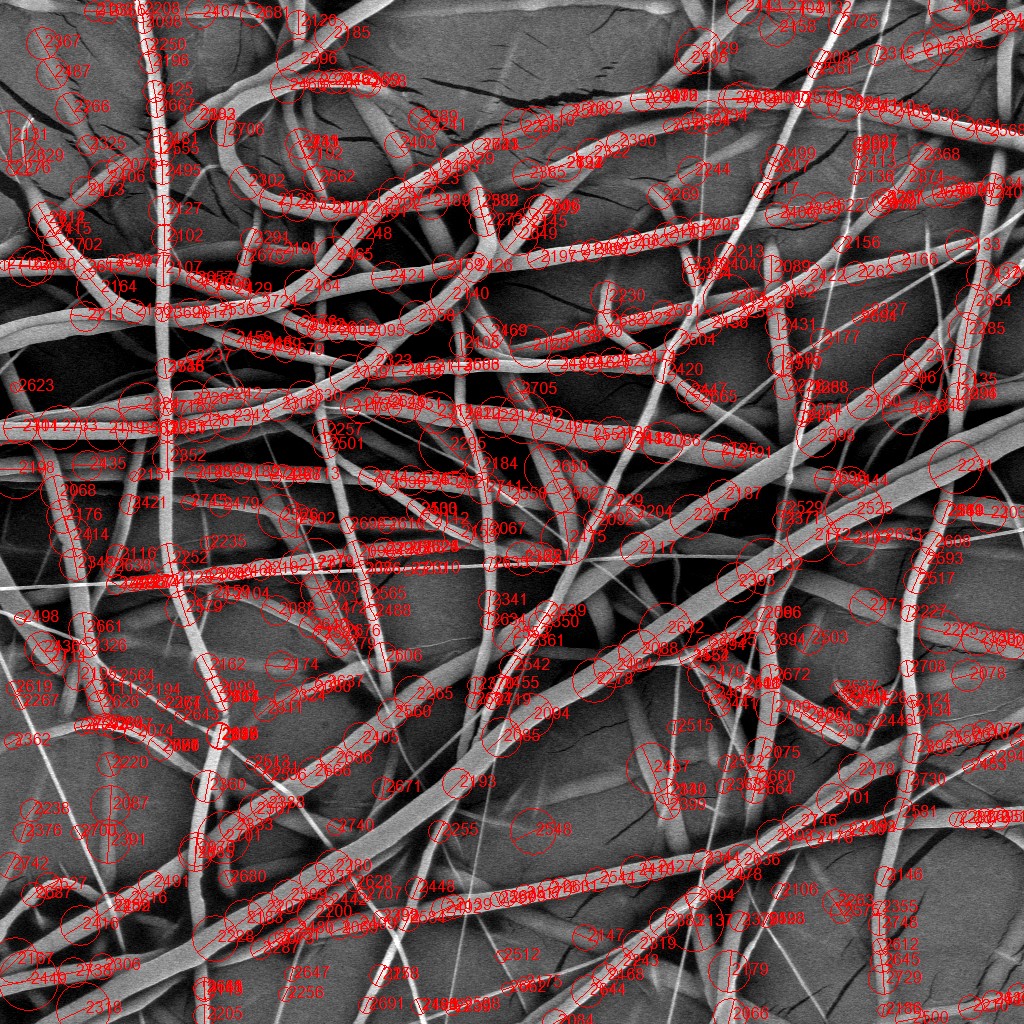

Supplement: S1 Data — (GZ) [file pone.0282903.s002.gz › data/FiberDiameter/Fiber analysis at 5,000X - Edge/Edge, 1,100rpm at 5,000X/1,100rpm, Fiber diameter_5,000X__20220127142849/Export/FibermetricImageMeasurements_883a666f-c4ae-4c8a-ae06-ef5b8b640a49.jpg]

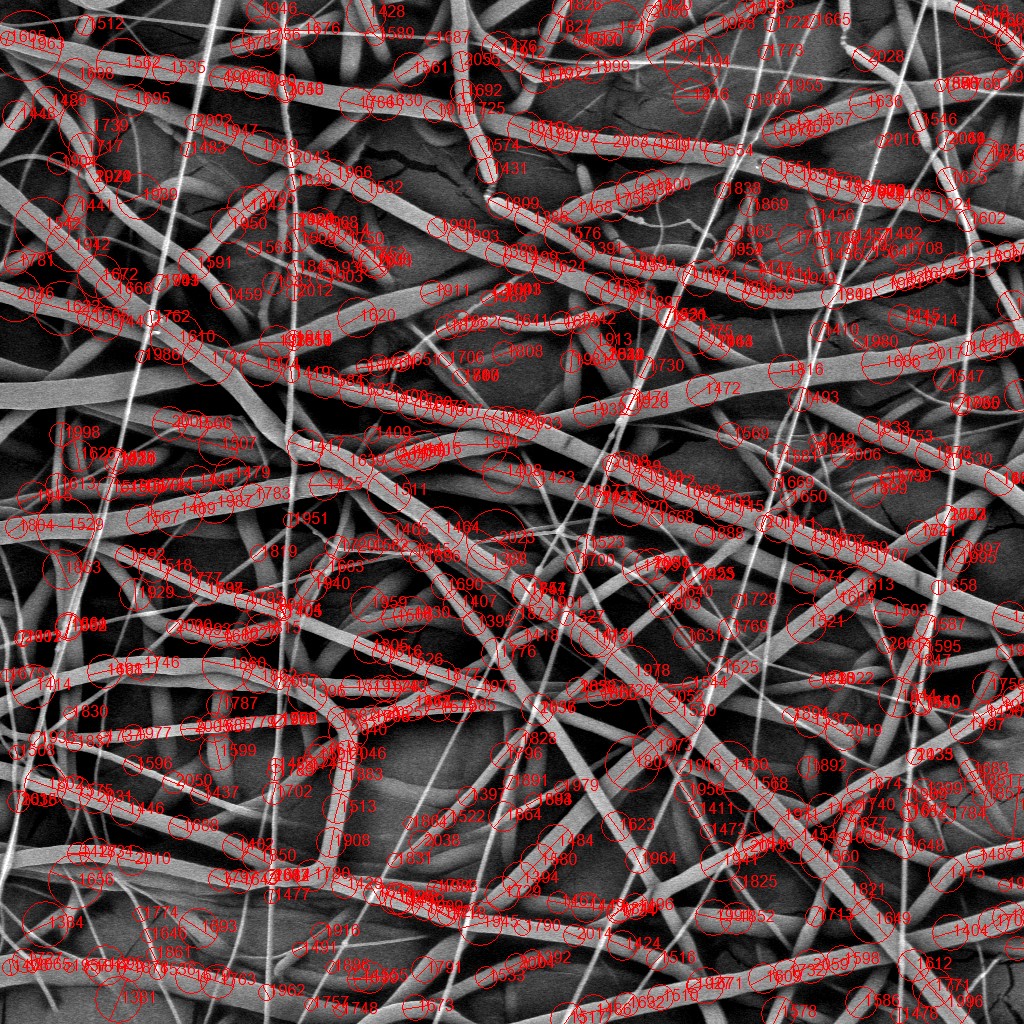

Supplement: S1 Data — (GZ) [file pone.0282903.s002.gz › data/FiberDiameter/Fiber analysis at 5,000X - Edge/Edge, 1,100rpm at 5,000X/1,100rpm, Fiber diameter_5,000X__20220127142849/Export/FibermetricImageMeasurements_e3cf3cf8-11b2-47fc-ac3b-5494df0da553.jpg]

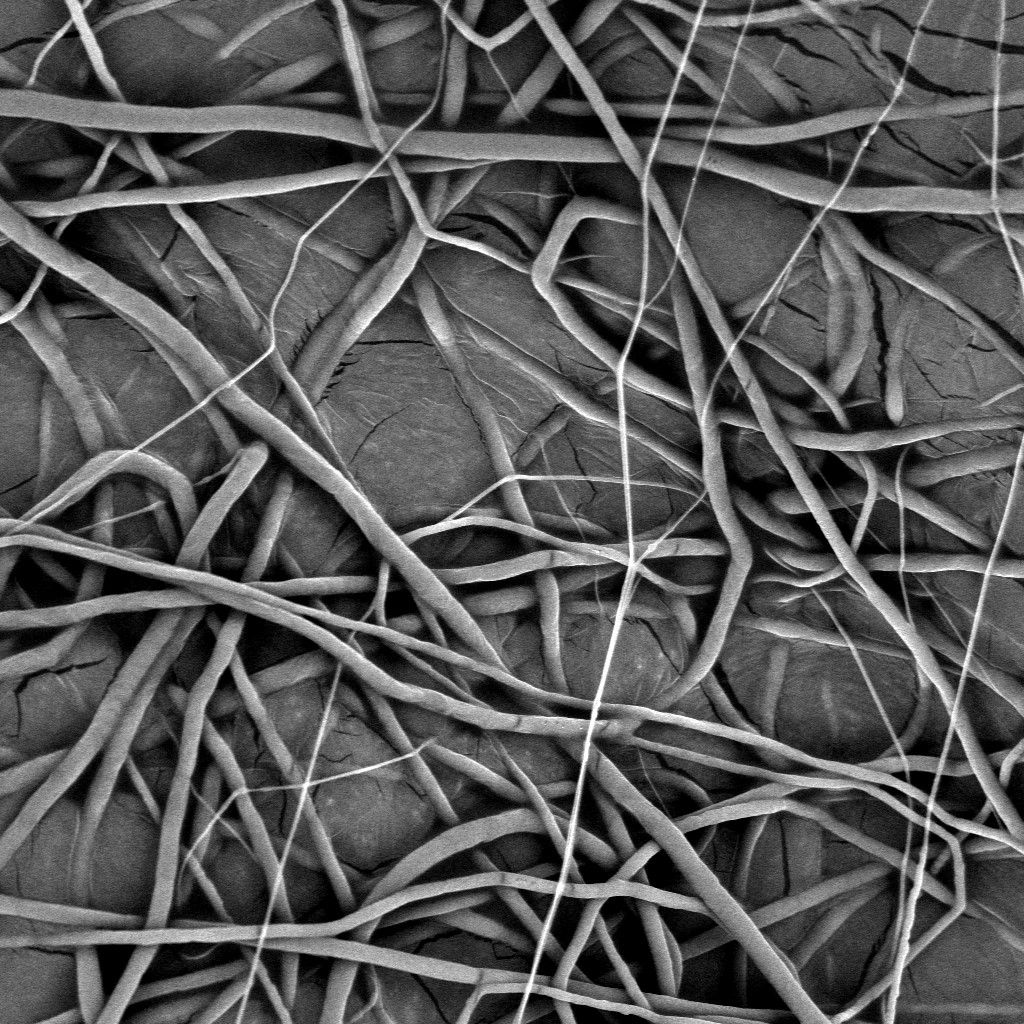

Supplement: S1 Data — (GZ) [file pone.0282903.s002.gz › data/FiberDiameter/Fiber analysis at 5,000X - Edge/Edge, 1,100rpm at 5,000X/1,100rpm, Fiber diameter_5,000X__20220127142849/Export/Fibermetric_Image0001.jpg]

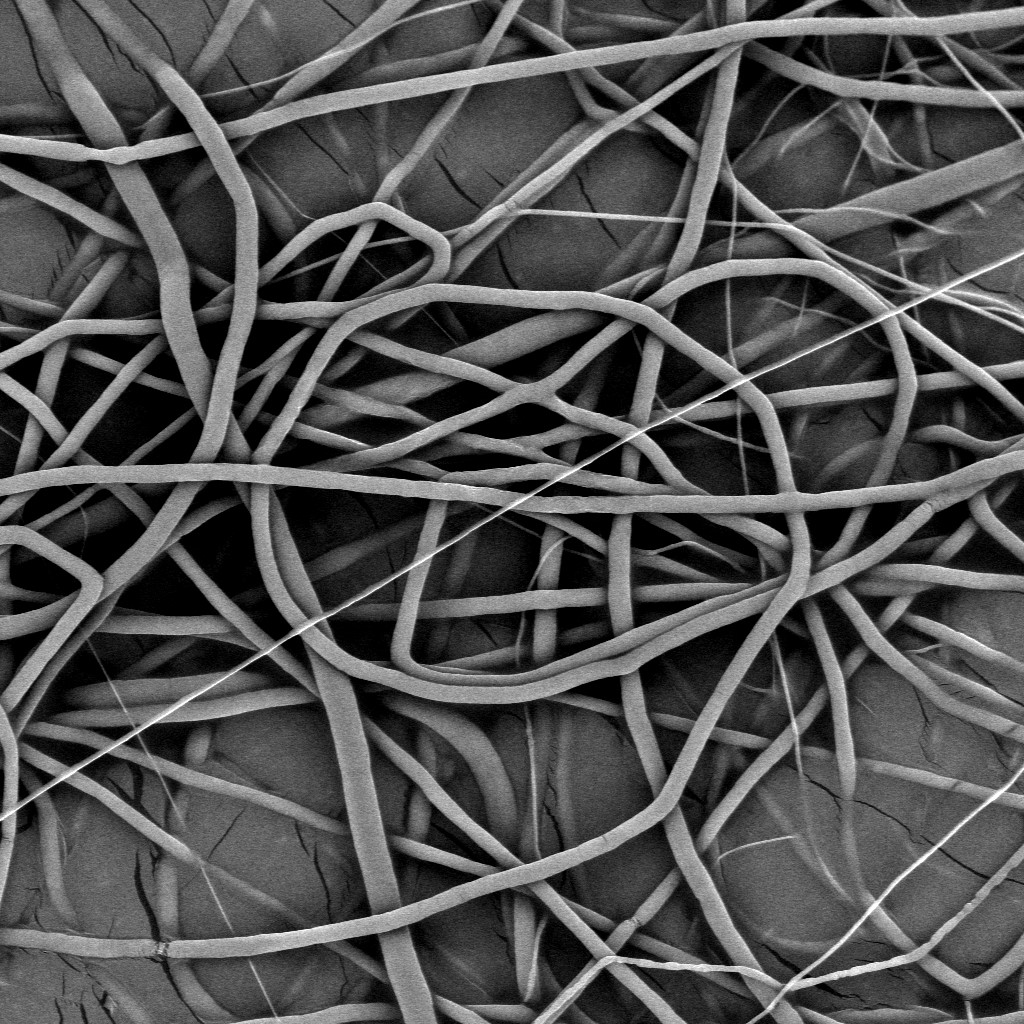

Supplement: S1 Data — (GZ) [file pone.0282903.s002.gz › data/FiberDiameter/Fiber analysis at 5,000X - Edge/Edge, 1,100rpm at 5,000X/1,100rpm, Fiber diameter_5,000X__20220127142849/Export/Fibermetric_Image0002.jpg]

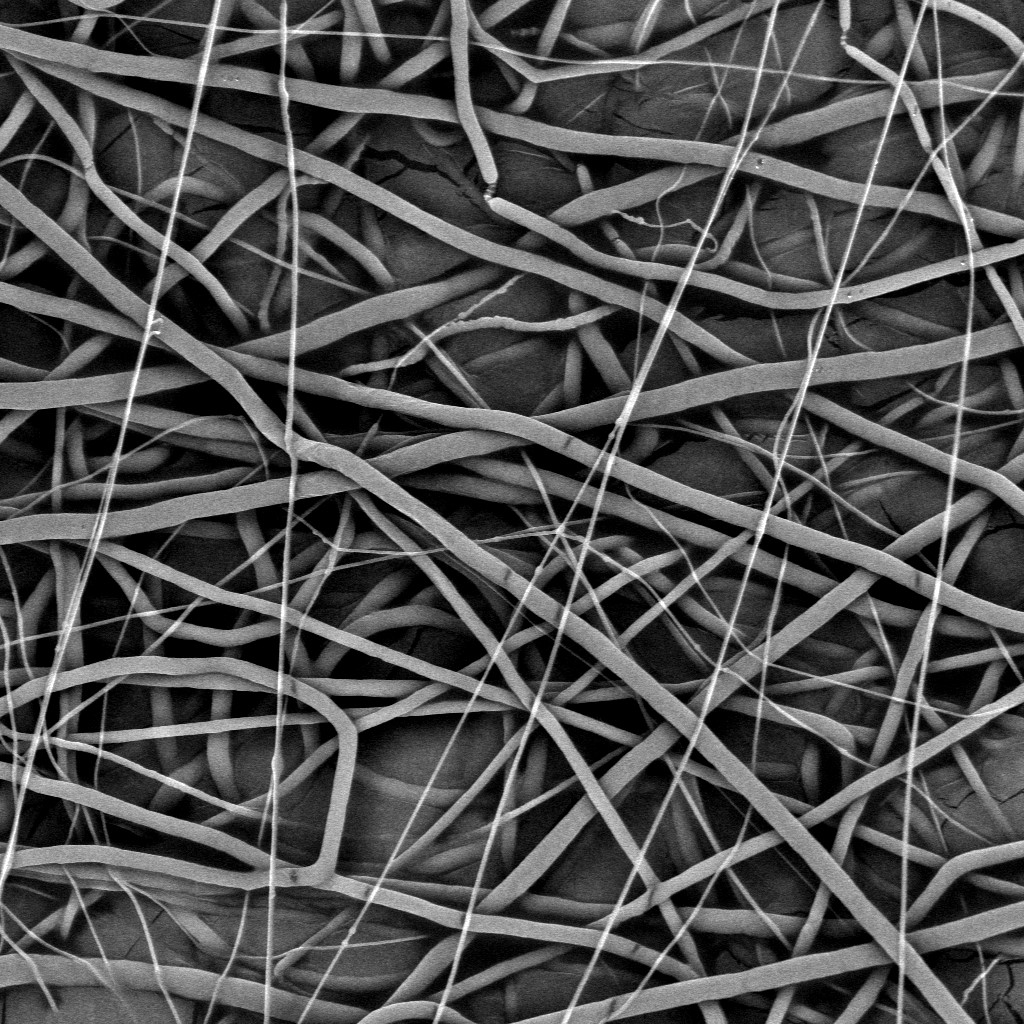

Supplement: S1 Data — (GZ) [file pone.0282903.s002.gz › data/FiberDiameter/Fiber analysis at 5,000X - Edge/Edge, 1,100rpm at 5,000X/1,100rpm, Fiber diameter_5,000X__20220127142849/Export/Fibermetric_Image0003.jpg]

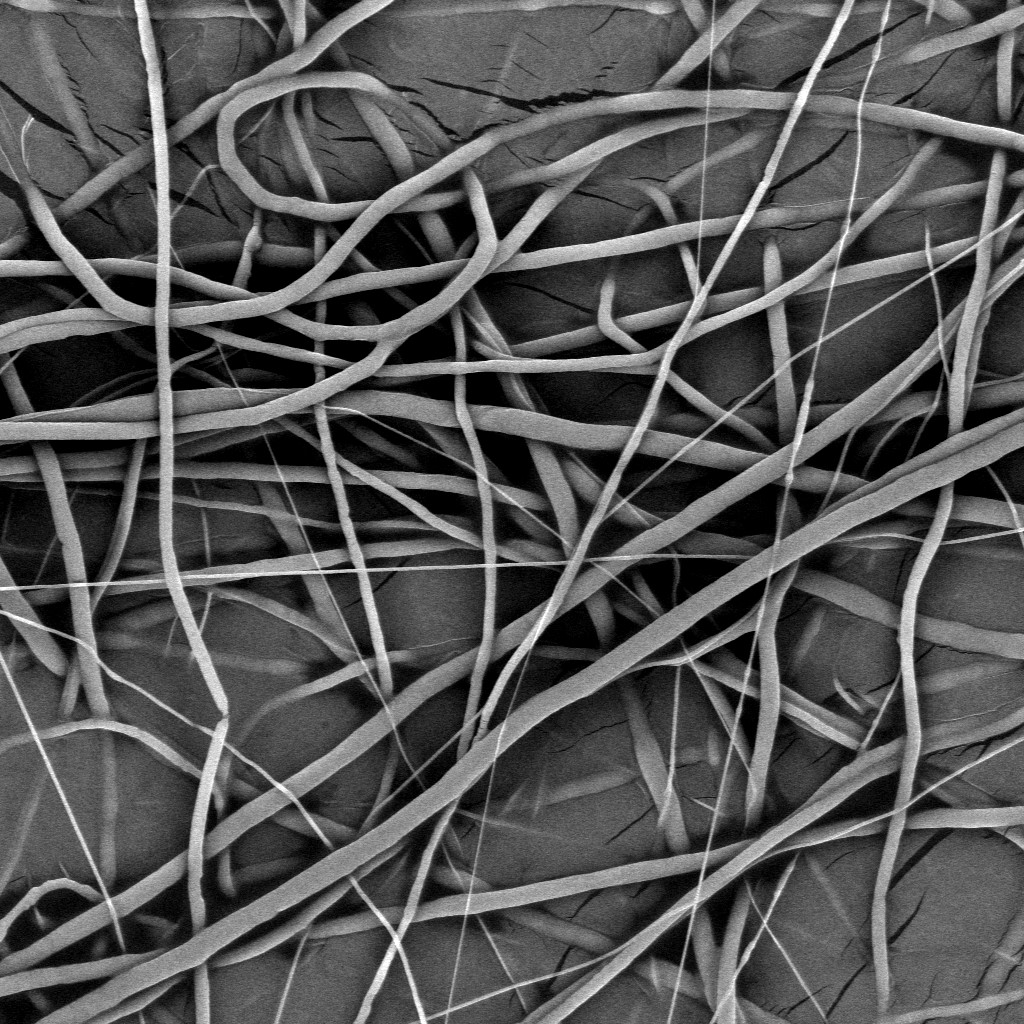

Supplement: S1 Data — (GZ) [file pone.0282903.s002.gz › data/FiberDiameter/Fiber analysis at 5,000X - Edge/Edge, 1,100rpm at 5,000X/1,100rpm, Fiber diameter_5,000X__20220127142849/Export/Fibermetric_Image0004.jpg]

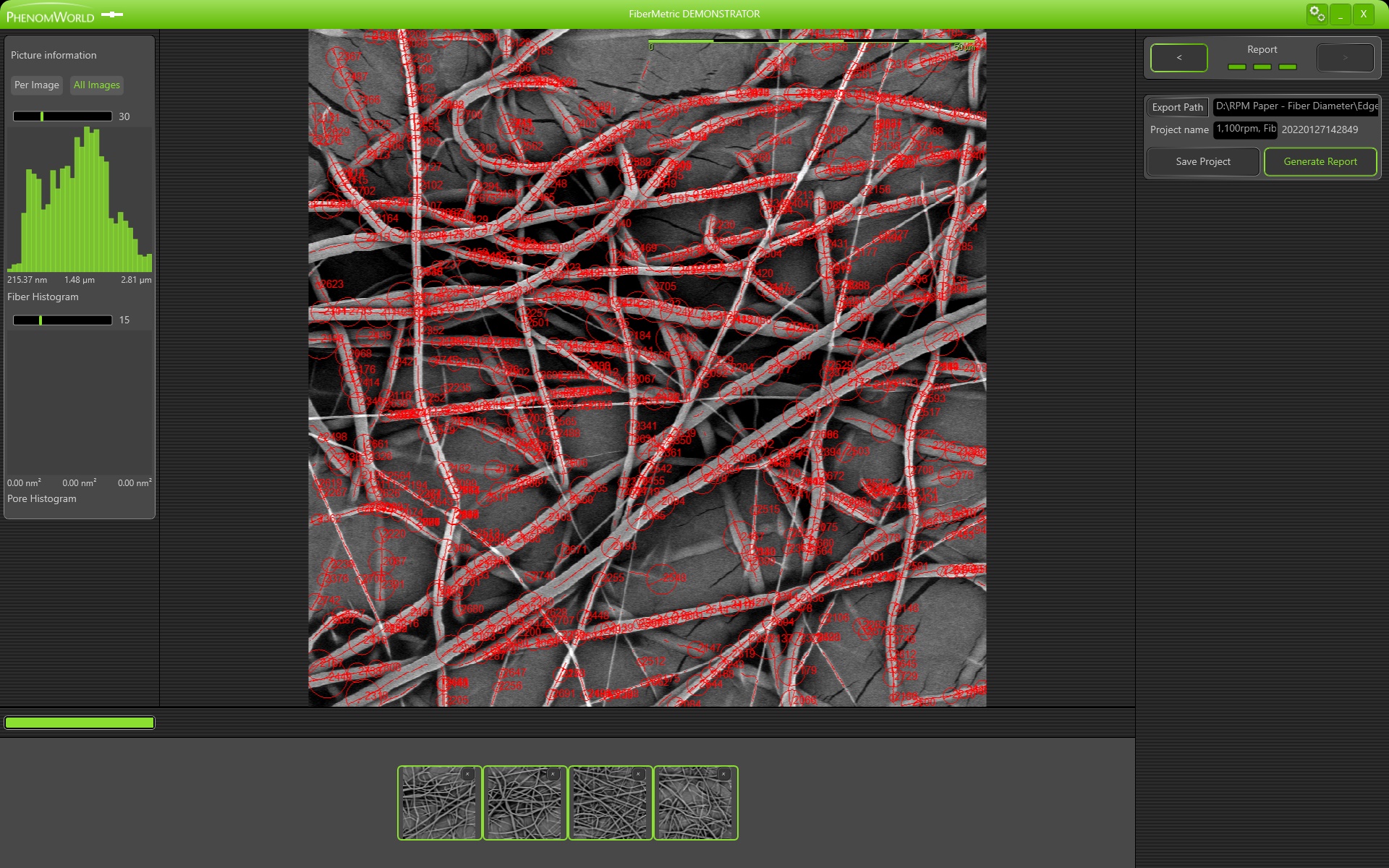

Supplement: S1 Data — (GZ) [file pone.0282903.s002.gz › data/FiberDiameter/Fiber analysis at 5,000X - Edge/Edge, 1,100rpm at 5,000X/1,100rpm, Fiber diameter_5,000X__20220127142849/Export/Screenshot.jpg]
